# Supplementary material for: Retention among transgender women treated with dolutegravir associated with tenofovir/lamivudine or emtricitabine in Argentina: TransViiV study
Source: PLoS One. 2023 Jan 20;18(1):e0279996. doi: 10.1371/journal.pone.0279996 (PMC9858466; doi:10.1371/journal.pone.0279996)
Supplement: S1 File — (PDF) [file pone.0279996.s001.pdf]

Tratamiento con Dolutegravir asociado a Tenofovir/Lamivudina o Emtricitabina en mujeres trans infectadas con HIV: estudio piloto

Investigador Principal: Dr. Omar Sued

Fundación Huésped

Domicilio del Investigador Principal: Pje. Gianantonio (Ex Ángel Peluffo) 3932 (C1202ABB)

Ciudad Autónoma de Buenos Aires, Argentina.

Teléfono del Investigador Principal: +54 11 4981-1855/7777

Fax del Investigador Principal: +54 11 4982 4024

e-mail del Investigador Principal: omar.sued@huesped.org.ar

1 Protocolo de Estudio Clínico

Tratamiento con Dolutegravir asociado a Tenofovir/Lamivudina o Emtricitabina en mujeres trans infectadas con HIV: estudio piloto

Número de Protocolo	FH-17
Producto en Investigación	Dolutegravir-Tenofovir-Emtricitabina/Lamivudina
Fecha	22/JUL/2015
Diseño de Investigación	Prospectivo, abierto, ensayo exploratorio de una sola rama
Investigadores	Omar Sued, MD
Patrocinador	Fundación Huésped*

Este estudio se llevará a cabo de conformidad con el protocolo, las Buenas Prácticas Clínicas y demás requerimientos regulatorios aplicables, incluido el archivo de documentos esenciales.

* Este estudio se realiza con un subsidio irrestricto de Laboratorio ViiV , Middlesex, Reino Unido

2 Índice

1 PROTOCOLO DE ESTUDIO CLÍNICO ... 2

2 ÍNDICE ... 3

3 INTRODUCCIÓN ... 5

4 OBJETIVOS DEL ESTUDIO ... 8

4.1 OBJETIVOS ... 8
4.1.1 Objetivo Primario ... 8
4.1.2 Objetivo Secundario ... 8
4.2 VARIABLES PRIMARIAS DE EVALUACIÓN (*PRIMARY ENDPOINT*) ... 8
4.3 VARIABLES SECUNDARIAS DE EVALUACIÓN ... 9

5 PLAN DE LA INVESTIGACIÓN ... 9

5.1 DISEÑO Y ESQUEMA GENERAL DE ESTUDIO: DESCRIPCIÓN ... 9
5.2 POBLACIÓN DE ESTUDIO ... 10
5.3 TAMAÑO DE LA MUESTRA ... 10
5.4 CRITERIOS DE INCLUSIÓN ... 10
5.5 CRITERIOS DE EXCLUSIÓN ... 11
5.6 REMOCIÓN DE PACIENTES DEL TRATAMIENTO O EVALUACIÓN ... 12
5.7 TRATAMIENTO ... 13
5.8 IDENTIDAD DEL PRODUCTO EN INVESTIGACIÓN ... 13
5.9 DEFINICIÓN DE FALLO VIROLÓGICO ... 13

6 PROCEDIMIENTOS DEL ESTUDIO ... 14

6.1 PROCEDIMIENTOS EN LA VISITA DE SELECCIÓN ... 14
6.2 PROCEDIMIENTOS EN LAS VISITA BASAL Y DE SEMANAS 24 Y 48 ... 15
6.3 PROCEDIMIENTOS EN LAS VISITAS DE SEMANAS 4, 8, 12 Y 36 ... 16
6.4 PROCEDIMIENTOS EN LA VISITA FINAL O DISCONTINUACIÓN ... 16
6.5 ANÁLISIS DE LABORATORIO ... 17
6.5.1 Tabla 1. Exámenes de Laboratorio ... 17
6.5.2 Tabla 2. Esquema de Procedimientos ... 18
6.6 TRATAMIENTO PREVIO Y CONCOMITANTE ... 18
6.7 ADHERENCIA AL TRATAMIENTO ... 19

7 VARIABLES DE SEGURIDAD ... 19

7.1 EXPERIENCIAS ADVERSAS/EVENTOS ADVERSOS ... 19
7.2 LIPODISTROFIA ... 21
7.3 REACCIÓN ALÉRGICA ... 22
7.4 EVENTO ADVERSO SERIO ... 22
7.4.1 Tabla 3: Tiempos para el reporte de EAS ... 24
7.5 IDEAS O CONDUCTAS SUICIDAS ... 24
7.6 MANEJO DE TOXICIDAD ... 25
7.6.1 Grados 1-2 ... 25
7.6.2 Grados 3-4 ... 25
7.7 REGLAS DE TOXICIDAD HEPÁTICA PARA DISCONTINUACIÓN DEL TRATAMIENTO ... 26
7.8 HIPERGLUCEMIA ... 28
7.9 PANCREATITIS/AMILASA PANCREÁTICA GRADO 3-4 ... 28
7.10 EVENTOS CLÍNICOS RELACIONADOS CON HIV ... 29

8 CONSIDERACIONES ESTADÍSTICAS ... 29

8.1 PLAN DE ANÁLISIS ESTADÍSTICO ... 29
8.1.1 Nivel de Significancia ... 31
8.1.2 Procedimiento para el Manejo de Datos Perdidos ... 31

8.1.3 Procedimiento para el Reporte de Desvíos del Plan Estadístico Original 31
8.1.4 Criterio de Selección de Pacientes que Deben ser Incluidas en el Análisis 31

9 ACCESO DIRECTO AL DATO FUENTE/DOCUMENTOS 32

10 CONDUCCIÓN ÉTICA DEL ESTUDIO 32

10.1 CONSENTIMIENTO INFORMADO 32
10.2 CONDUCCIÓN DEL ESTUDIO 32

11 MANEJO Y ALMACENAMIENTO DE DATOS OBTENIDOS 33

12 GARANTÍA DE LA CALIDAD DE LOS DATOS 33

13 ANEXOS 34

13.1 ANEXO A. SINOPSIS DEL PROTOCOLO 34
13.2 ANEXO B. TOXICIDAD CLÍNICA 36
13.3 ANEXO C. MEDICACIONES NO PERMITIDAS 55
13.4 ANEXO D. LISTADO DE MANIFESTACIONES ESPERABLES CONOCIDAS DE INFECCIÓN DE HIV. 56
13.5 ANEXO E. DOCUMENTOS NECESARIOS PREVIOS A LA INICIACIÓN DEL ESTUDIO 58
13.6 ANEXO F. RESPONSABILIDADES DEL CENTRO (FUNDACIÓN HUÉSPED) 59
13.7 ANEXO G. RESPONSABILIDADES DEL INVESTIGADOR CLÍNICO 60
13.8 ANEXO H. ELEMENTOS DEL CONSENTIMIENTO INFORMADO 61
13.9 ANEXO I. CUESTIONARIOS DEL ESTUDIO 64

14 REFERENCIAS 120

3 Introducción

Las mujeres transgénero (mujeres trans) siguen siendo una población clave dentro de la pandemia mundial del HIV. Ellas sostienen tasas excepcionalmente altas de infección por HIV, impulsadas simultáneamente por el consumo de drogas y la vía sexual (1-6). Estos riesgos se ven agravados por las diversas condiciones sociales y estructurales, como la violencia, el estigma y la discriminación (7). A nivel mundial, las estimaciones recientes sugieren que las mujeres trans tienen 49 veces más riesgo de infección por HIV en comparación con la población adulta general (1). Por lo tanto, existe una urgente necesidad de una nueva investigación que identifique e informe intervenciones operacionales a nivel individual, social y estructural con el objetivo de reducir la morbilidad y la transmisión del HIV entre las mujeres trans.

Argentina tiene una epidemia concentrada de HIV, con una prevalencia del 0,4% en la población general y una prevalencia mayor entre grupos específicos, principalmente la comunidad transgénero, que corresponde al grupo más afectado. La prevalencia de HIV en mujeres trans es del 34%, significativamente mayor que en los hombres que tienen sexo con hombres (11,4%), los usuarios de drogas (7%) y las mujeres trabajadoras sexuales (6%) (8). La vulnerabilidad es asombrosamente elevada entre mujeres trans demostrada por una esperanza de vida de aproximadamente 35 años en Buenos Aires, que contrasta con una esperanza de vida de 79 años en otras mujeres (9, 10).

El uso de drogas, la prostitución, las experiencias de violencia sexual, los implantes de siliconas no supervisados, los problemas legales, la tuberculosis y otras enfermedades infecciosas como sífilis y hepatitis, y las enfermedades mentales, también son más frecuentes entre las mujeres trans, lo que contribuye a un mayor estigma externo y mayor morbilidad y mortalidad en esta población. El estigma relacionado con el género, la discriminación y "transfobia", en particular en el sector de la salud, dificulta aún más el acceso a este servicio (11).

El alto riesgo de esta población puede ser ejemplificado en el resultado de una reciente encuesta a nivel nacional entre 452 mujeres trans. La prevalencia de HIV, por auto-reporte, fue del 27%, pero fue dos veces más alta entre las que informaron el uso inconsistente del preservativo con los clientes, una vivienda inestable y

experiencias de violencia policial, destacando la complejidad de abordar los métodos tradicionales de prevención del HIV entre esta población (12).

El aumento de la evidencia demuestra que la provisión de tratamiento para el HIV en personas con serología positiva, además de prevenir la progresión de la enfermedad y la muerte, reduce en gran medida la transmisión a las parejas sexuales (13).

El diagnóstico y el tratamiento universal para prevenir la transmisión del HIV ha ganado considerable atención debido a sus beneficios para la salud pública, sin embargo, es necesario un acceso rápido a las combinaciones de antirretrovirales sencillos y seguros, pruebas de HIV de rutina a disposición de la mayoría de la población, así como la viabilidad de un tratamiento inmediato para los que están identificados como infectados (14). Los beneficios de este enfoque —conocido como tratamiento como prevención (*TasP* por sus siglas en inglés) — serían aún mayores para poblaciones de alto riesgo, como es el caso de las mujeres trans, lo que representa uno de los mayores grupos de la epidemia en América Latina. Sin embargo, a pesar de estos beneficios potenciales, las preguntas sin respuesta acerca de su viabilidad operativa y aceptabilidad continúan generando debate.

El entorno de riesgo donde viven las mujeres trans, que involucra el consumo de alcohol y drogas, el trabajo sexual, la alta exposición a la violencia, la discriminación y el estigma, pone en peligro la expansión de la terapia antirretroviral (TAR) y hace difícil la adherencia; lo que limita la eficacia de este enfoque (15).

Además, la auto-prescripción de hormonas y otros tratamientos, una práctica común dentro de la comunidad transgénero, puede dar lugar a interacciones medicamentosas, complicando aún más la eficacia de la TAR (16). Entre 69 mujeres trans HIV positivas registradas en nuestro hospital, sólo el 46% inició TAR dentro del primer año de diagnóstico de HIV. Entre las que recibieron prescripción de TAR, sólo el 38% pudo mantener la supresión virológica a las 48 semanas de tratamiento (16% del total), destacando la necesidad de comprender mejor los factores sociales, estructurales y ambientales que afectan los resultados del HIV entre las poblaciones de alto riesgo (17).

A la luz de las consideraciones anteriores, hay una urgente necesidad de explorar los posibles riesgos y beneficios del enfoque TasP entre mujeres trans. Algunas de las preguntas puntuales de la investigación incluyen: a) ¿cúal es el régimen más

adecuado en términos de potencia, dosificación, tolerabilidad, efectos secundarios e interacciones medicamentosas?, b) ¿cuál es la aceptabilidad de la TAR entre mujeres trans?, y c) ¿cuáles son los otros factores contextuales e individuales que pueden facilitar o impedir la aceptabilidad, la adherencia y la retención de los regímenes de TAR entre las mujeres trans?

Aunque los países en desarrollo no están considerando los inhibidores de la integrasa (INIs) como tratamiento de primera línea, regímenes basados en dolutegravir (DTG) deben ser considerados como esquemas preferidos de primera línea para esta población por varias razones. DTG es un compuesto antiviral muy potente con eficacia clínica demostrada tanto en pacientes naïve (pacientes que nunca recibieron TAR previamente) como en pacientes previamente tratados, con una demostrada barrera genética más alta que otro INI (18).

El excelente perfil de seguridad y tolerancia podría facilitar una mejor adherencia que otros regímenes en esta población en particular y la ausencia de interacciones significativas entre fármacos, hace que las combinaciones basadas en DTG sean elegibles para individuos con comorbilidades importantes o que potencialmente consumen otras drogas. Además, la posibilidad de una dosis al día hace que los regímenes basados en DTG sean extremadamente atractivos para esta población.

Tenofovir disoproxil fumarato-emtricitabina (TDF-FTC) o lamivudina (TDF-3TC) son combinaciones de una dosis fija una vez al día de análogos nucleótido y nucleósido inhibidores de la transcriptasa inversa, que ha demostrado eficacia en ensayos clínicos bien diseñados con un buen seguimiento. TDF-FTC o TDF-3TC combina los beneficios de una menor toxicidad, la sencillez de dosificación y propiedades farmacocinéticas favorables (19).

Aunque en breve una combinación triple de drogas (DTG, abacavir, lamivudina) estará disponible en un futuro cercano, las dificultades para tener un acceso oportuno a la prueba HLA B5701 para descartar hipersensibilidad al abacavir, podría limitar su indicación.

Por lo tanto, se plantea la hipótesis de que un régimen basado en DTG-TDF-FTC o 3TC sería un régimen eficaz en mujeres trans, infectadas por el HIV-1, naïve de tratamiento antirretroviral, con un buen perfil de aceptabilidad, tolerancia y seguridad,

y que daría a un porcentaje significativo de personas la posibilidad de mantener este régimen durante al menos 48 semanas.

4 Objetivos del Estudio

4.1 Objetivos

4.1.1 Objetivo Primario

El **objetivo primario** de este estudio piloto es determinar la retención en la atención de salud de las mujeres trans bajo tratamiento con DTG-TDF-FTC o 3TC.

4.1.2 Objetivo Secundario

a. Evaluar la eficacia del régimen antirretroviral a las 48 semanas;
b. Describir la seguridad y tolerabilidad de este régimen;
c. Evaluar la adherencia a través de 48 semanas;
d. Determinar la satisfacción de la paciente con este régimen;
e. Identificar los factores individuales, sociales y contextuales asociados con la adherencia y la retención.

4.2 Variables Primarias de Evaluación (*Primary Endpoint*)

El objetivo principal será evaluado a través de la proporción de individuos que brinden información sobre el uso de TAR al final del estudio:

a. Retención en la atención: Proporción de personas inscriptas y tratadas que brinden información clínica hasta las 48 semanas de seguimiento.
b. Retención en el tratamiento: Proporción de personas inscriptas y tratadas que reciben los fármacos del estudio hasta las 48 semanas de seguimiento.

4.3 Variables Secundarias de Evaluación

Los objetivos secundarios se evaluarán utilizando los siguientes criterios de valoración:

a. Proporción de pacientes con niveles de ARN-HIV menores a 50 copias/mL en un análisis de intención de tratar a la semana 48 utilizando el algoritmo *Snapshot* de la FDA (dato faltante, cambio o discontinuación de tratamiento son considerados fallo) en las pacientes que al menos hayan tomado una dosis del medicamento;
b. Frecuencia, tipo y gravedad de los eventos adversos y alteraciones del laboratorio;
c. Recuento de pastillas, escala analógica visual para la adhesión en cada visita;
d. Cambios en las puntuaciones de las escalas de estigma y discriminación, calidad de vida, apoyo social, ansiedad y depresión (Berger, WBI, DUKE, CES-D, STAI por sus siglas en inglés) al inicio del estudio y en las semanas 4, 24 y 48 de seguimiento;
e. Cambios en las puntuaciones de las escalas de comportamiento sexual, uso de drogas y alcohol al inicio de estudio y en cada visita de seguimiento;
f. Asociación de características basales individuales, sociales y contextuales con el porcentaje de adherencia y retención a las 48 semanas.

5 Plan de la Investigación

5.1 Diseño y Esquema General de Estudio: Descripción

Ensayo prospectivo, abierto, de una sola rama con DTG-TDF-FTC o 3TC en pacientes HIV positivas naïve de tratamiento.

Procedimientos (ver Tabla 2): Las pacientes serán identificadas de forma activa a través de campañas de testeo en la comunidad y el acceso a los servicios de HIV serán facilitados en la Fundación Huésped.

Después de dar su consentimiento a la participación, las pacientes serán evaluadas y recibirán una nueva cita para el inicio del TAR (si es elegible).

5.2 Población de Estudio

Las pacientes serán HIV-1 positivas, ≥18 años de edad, sin tratamiento previo, auto-identificadas como mujeres trans que cumplan con todos los criterios de inclusión y ninguno de los criterios de exclusión.

5.3 Tamaño de la Muestra

Al tratarse de un estudio piloto, estudio de prueba de concepto, que eventualmente podría ser seguido por un estudio multicéntrico más grande, no se realiza cálculo formal del tamaño de la muestra, sino una muestra por conveniencia.
Una muestra de 60 mujeres trans HIV positivas, naïve de tratamiento, fue seleccionada en base a la factibilidad para incluirse en el sitio de estudio. Este estudio piloto permitirá evaluar el diseño del protocolo, obtener datos preliminares y apoyar el desarrollo y la validación de los formularios de evaluación individual que se utilizarán en otros ensayos clínicos más amplios. Los detalles adicionales se comentan en el Plan de Análisis Estadístico.

5.4 Criterios de Inclusión

Las pacientes serán elegibles para participar en el estudio si cumplen con TODOS los siguientes criterios:

a. Paciente con infección por HIV-1 documentada por al menos dos pruebas serológicas diferentes (test rápido, ELISA, Western Blot) o una carga viral superior a 3.000 copias/mL.
b. Edad ≥18 años.
c. Auto-identificadas como mujeres trans.
d. Naïve de tratamiento antirretroviral.

e. La paciente ha firmado voluntariamente y fechado el formulario de consentimiento informado aprobado por el Comité de Ética.

5.5 Criterios de Exclusión

Las pacientes NO serán elegibles para participar en este estudio si cumplen con CUALQUIERA de los siguientes criterios:

a. Evidencia de resistencia a emtricitabina, lamivudina y/o tenofovir según el Panel de Resistencia IAS- USA 2013.
b. Uso de alcohol o drogas que, en la opinión del investigador, podría comprometer la seguridad de la paciente o su adherencia al protocolo.
c. Uso concomitante de agentes hipolipemiantes, interferón, interleukina-2, quimioterapia citotóxica, dofetidilo (o pilsicainida), inmunosupresores, antiácidos que contengan Ca++ o Mg++ al momento de la visita de selección o cualquier otra medicación no permitida.
d. Infección oportunista (categoría C del CDC) o cualquier otra enfermedad o condición clínica que, en opinión del investigador, podría comprometer la seguridad de la paciente o el resultado del estudio o la adherencia a las normas del protocolo, incluyendo neoplasias, con excepción de sarcoma de Kaposi cutáneo, carcinoma basocelular, neoplasia cervical intraepitelial o carcinoma escamoso no invasivo resecado.
e. Uso de cualquiera de los siguientes agentes dentro de los 28 días de la visita de selección: quimioterapia citotóxica, radioterapia, cualquier inmunomodulador que altere la respuesta inmune, tratamiento con vacuna inmunoterapéutica para HIV-1 dentro de los 90 días de la visita de selección o exposición a drogas experimentales o vacunas experimentales dentro de los 28 días, cinco vidas medias del agente testeado, o el doble de tiempo de acción del agente testeado antes de la primer dosis de este estudio (lo que sea más prolongado en el tiempo).
f. Contraindicación para cualquiera de los fármacos del estudio (historia de enfermedades renales, anomalías de laboratorio de grado 4 o cualquier otra condición clínica previa que, en opinión del investigador, podría hacer que la

paciente no sea apta para el estudio o no pueda cumplir con los requisitos de dosificación).

g. Necesidad de tratamiento para hepatitis C (HCV) durante el período que dura el estudio.
h. Clearance de creatinina <50 mL/min según fórmula de Cockroft-Gault.
i. Pacientes con daño hepático moderado o severo (Child-Pugh clase B o mayor).

5.6 Remoción de Pacientes del Tratamiento o Evaluación

Las pacientes pueden ser retiradas del estudio si:

a. Un evento adverso serio (EAS) ocurre;
b. La paciente ya no se beneficia con el tratamiento de acuerdo a la opinión del investigador.

Las pacientes deben ser retiradas del estudio si:

a. Retira su consentimiento;
b. Presenta carga viral confirmada ≥1.000 copias/mL en la semana 24 o en la semana 36;
c. Presenta rebote confirmado de la carga viral ≥200 copias/mL una vez alcanzada la indetectabilidad (<50 copias/mL);
d. Presenta alguna de las reglas de detención debidas a daño hepático (ver Sección 7.7);
e. El investigador considera que, por razones de seguridad, lo mejor para la paciente es que sea retirada del estudio;
f. Reacción alérgica grado ≥3 que se considera posible o probablemente relacionada a las drogas de investigación;
g. Rash grado 3 o 4;
h. Cualquier rash que se asocia a un aumento de ALT ≥2.

La fecha y el motivo de la discontinuación deben registrarse en la hoja de recogida de datos (*CRF*, por sus siglas en inglés). A menos que la paciente retire su consentimiento, deberá hacer una visita de final de estudio/discontinuación.

5.7 Tratamiento

Dolutegravir 50 mg más la co-formulación de lamivudina 300 mg o emtricitabina 200 mg con tenofovir 300 mg, una vez al día con alimentos.

5.8 Identidad del Producto en Investigación

Dolutegravir:
Comprimidos recubiertos de 50 mg
Dosis y régimen: 50 mg una vez por día

Emtricitabina (FTC) o Lamivudina (3TC):
200 mg o 300 mg respectivamente, co-formulado con comprimido de TDF

Tenofovir (TDF):
300 mg co-formulado con comprimido de FTC o 3TC
Dosis y régimen: TDF-FTC o 3TC comprimido co-formulado una vez por día.
Dolutegravir será provisto por ViiV. TDF-FTC o 3TC será provisto por el Ministerio de Salud o el proveedor regular.

5.9 Definición de Fallo Virológico

Se define fallo virológico como:

a. Rebote de la carga viral confirmado ≥200 copias luego de haber alcanzado <50 copias/mL;
b. Carga viral confirmada ≥1.000 copias/mL en la semana 24;
c. Carga viral confirmada ≥50 copias/mL en la semana 48.

En el caso que una paciente llegue a una de estas situaciones de fallo virológico, se deberá repetir la carga viral con una nueva muestra obtenida no menos de 7 y no más allá de los 30 días. Si el fallo se confirma, se deberá realizar test de resistencia para evaluar el número y tipo de mutaciones de resistencia.

6 Procedimientos del Estudio

Luego que la paciente haya firmado y fechado el consentimiento informado específico del estudio, se le realizarán los siguientes procedimientos:

6.1 Procedimientos en la Visita de Selección

a. Antecedentes médicos: durante la visita de selección, se hará un repaso de todos los antecedentes médicos de la paciente, incluyendo uso de tabaco, drogas y alcohol, enfermedades marcadoras y eventos adversos relacionados a drogas.
b. Examen físico completo, incluyendo medición de peso y altura, y determinación de los signos vitales en posición sentada (presión arterial, pulso y temperatura).
c. Determinaciones de laboratorio:
    - Carga viral plasmática ARN-HIV, medida utilizando un método validado (*Abbott real time PCR*).
    - Recuento de células CD4 y CD8.
    - Exámenes clínicos de laboratorio: hematología, química completa, orina y serologías según figuran en la Tabla 1 y 2.
    - Genotipo para HIV (test de resistencia).
d. Se obtendrá un electrocardiograma (ECG) de 12 derivaciones con la paciente en reposo en la visita de selección y según resulte clínicamente necesario a lo largo del estudio. Un médico calificado del centro interpretará, firmará y fechará todos los ECG. La interpretación del ECG se hará en forma de comentario escrito utilizando las siguientes categorías:
    - ECG normal

- ECG anormal: no clínicamente significativo
- ECG anormal: clínicamente significativo

e. Relevamiento de medicación concomitante.

Los resultados de todas las evaluaciones clínicas durante el período de selección deben encontrarse dentro de los límites clínicamente aceptables, definidos por los exámenes de laboratorio y revisados por el investigador.

6.2 Procedimientos en las Visita Basal y de Semanas 24 y 48

a. Antecedentes médicos: se actualizarán los antecedentes médicos antes de administrar la droga del estudio y a medida que sea necesario a lo largo del estudio. También se registrará el uso de medicación (tanto por prescripción como de venta libre) durante la visita basal.
b. Examen físico completo: se realizará un examen físico completo en la visita basal, en la visita de semana 24, 48. El examen físico de la visita basal servirá como examen físico basal para el resto del estudio.
c. Medición de peso.
d. Determinación de los signos vitales en posición sentada: presión arterial, pulso y temperatura.
e. Determinaciones de laboratorio:
    - Carga viral plasmática ARN-HIV, medida utilizando un método validado (*Abbott real time PCR*).
    - Recuento de células CD4 y CD8
    - Exámenes Clínicos de Laboratorio: hematología, química completa y orina según figuran en las Tablas 1 y 2
    - Almacenamiento de plasma durante 10 años para estudios posteriores sobre marcadores serológicos de inflamación y otras serologías. No se utilizarán para estudios genéticos ni otros estudios no relacionados con el HIV o infecciones de transmisión sexual sin solicitar una nueva autorización.
f. Relevamiento de medicación concomitante.

g. Evaluación de adherencia mediante conteo de comprimidos y encuesta de adherencia (ACTG Modificado) en la semana 24 y 48.
h. Evaluación de eventos adversos.
i. Cuestionarios: comportamiento sexual, uso de drogas y alcohol, estigma y discriminación (Berger HIV Stigma Scale), calidad de vida (*Well-Being Index, WBI*), soporte social (*Social Support Scale Duke-UNC*), depresión e ideación suicida (Center for Epidemiological Studies – Depression scale (CES-D)+ 4-item suicidal-ideation screener), ansiedad (Inventario de ansiedad-rasgo STAI) y personalidad (Inventario de personalidad para el DSM-5, PID-5-BF)

6.3 Procedimientos en las Visitas de Semanas 4, 8, 12 y 36

a. Examen físico dirigido según síntomas:
   - Medición de peso
   - Determinación de los signos vitales en posición sentada: presión arterial, pulso y temperatura.
b. Determinaciones de laboratorio:
   - Carga viral plasmática ARN-HIV, medida utilizando un método validado (*Abbott real time PCR*)
   - Recuento de células CD4 y CD8 (solo en semanas 12 y 36)
   - Exámenes Clínicos de Laboratorio: hematología, química básica según esquema de procedimientos (Tabla 2).
   - Almacenamiento de plasma
c. Relevamiento de medicación concomitante
d. Evaluación de adherencia mediante conteo de comprimidos y encuesta de adherencia (cuestionario ACTG Modificado).
e. Cuestionarios: comportamiento sexual, uso de drogas y alcohol.
f. Evaluación de eventos adversos.

6.4 Procedimientos en la Visita Final o Discontinuación

a. Examen Físico completo.

b. Medición de peso.
c. Determinación de los signos vitales en posición sentada: presión arterial, pulso y temperatura.
d. Evaluación de eventos adversos.

6.5 Análisis de Laboratorio

Se obtendrán muestras para los análisis de laboratorio (Tabla 1) en las visitas especificadas en la Tabla 2.

Las pacientes deberán presentar un ayuno de al menos 10 horas (se permite beber agua) en las visitas basal, de semanas 24 y 48; para el resto de las visitas bastará con un ayuno de 8 horas. Si los resultados cumplen criterios para ser considerados toxicidades de Grado 3 o 4, o son considerados clínicamente significativos, se les hará un seguimiento hasta su resolución. El investigador debe revisar, firmar y fechar todos los reportes de laboratorio.

6.5.1 Tabla 1. Exámenes de Laboratorio

**Hematología**	**Química**	**Análisis de orina**	**Otras pruebas**
Hematocrito<br>Hemoglobina<br>Glóbulos rojos<br>Glóbulos blancos<br>Neutrófilos en cayado<br>Linfocitos<br>Monocitos<br>Basófilos<br>Eosinófilos<br>Plaquetas	Urea<br>Creatinina<br>Creatinina fosfoquinasa<br>Bilirrubina total<br>Bilirrubina directa<br>Bilirrubina indirecta<br>Transaminasa glutámico-pirúvica sérica (SGPT/ALT)<br>Transaminasa glutámico-oxaloacética sérica (SGOT/AST)<br>Fosfatasa alcalina<br>Colesterol total<br>Triglicéridos<br>HDL<br>LDL<br>Proteínas totales<br>Glucosa (en ayunas)<br>Albúmina<br>Amilasa sérica<br>Gama–GT<br>Bicarbonato<br>LDH<br>Sodio	Densidad urinaria<br>Cuerpos cetónicos<br>pH<br>Proteínas<br>Sangre<br>Glucosa<br>Urobilinógeno<br>Evaluación microscópica	HBsAg, anticore<br>Serología HCV<br>Carga viral HIV-1<br>Test de resistencia genotípica<br>CD4+<br>CD8+<br>Guardado de plasma<br>17 B estradiol, testosterona libre y total, dímero-D, IL6, sCD14, sCD163 y niveles hormonales

	Potasio<br>Calcio<br>Cloro		

6.5.2 Tabla 2. Esquema de Procedimientos

Procedimientos	Selección	Basal	Sem 4	Sem 8	Sem 12	Sem 24	Sem 36	Sem 48	Final
Consentimiento informado	X								
Historia médica	X								
Examen físico	X	X	X	X	X	X	X	X	X
Signos Vitales	X	X	X	X	X	X	X	X	X
ECG	X								
Medicación concomitante	X	X	X	X	X	X	X	X	
Escalas[1]		X				X		X	
Escalas [2]		X	X	X	X	X	X	X	
Cuestionarios psicosociales		X				X		X	
Eventos adversos			X	X	X	X	X	X	X
Adherencia[3]			X	X	X	X	X	X	
HIV Genotipo*	X								
Serologías[4]	X								
HIV-1 RNA	X	X	X	X	X	X	X	X	
Citometría de flujo (CD4 y CD8)	X	X			X	X	X	X	
Hematología[5]	X	X	X	X	X	X	X	X	
Química básica[6]			X	X	X		X		
Química completa[7]	X	X				X		X	
Otros marcadores		X				X		X	
Almacenamiento de plasma		X	X	X	X	X	X	X	

1) Berger HIV Stigma Scale, WBI, DUKE, CES-D, STAI, PID-5-BF
2) Comportamiento sexual, uso de drogas y alcohol
3) Conteo de comprimidos y cuestionario ACTG Modificado
4) Serologías: HBV anti core, HBsAg, HCV IgG, VDRL
5) Hematología: hemoglobina, hematocrito, recuento de eritrocitos (RBC), recuento de glóbulos blancos (WBC), plaquetas, neutrófilos, basófilos, linfocitos, monocitos, reticulocitos. Coagulograma.
6) Química básica: TGO, TGP, bilirrubina total, creatinina
7) Química completa: TGO, TGP, fosfatasa alcalina, amilasa, gama-GT, Na, K, Ca, Cloro, bicarbonato, bilirrubina total, bilirrubina directa, bilirrubina indirecta, urea, creatinina, CPK, colesterol total, HDL, LDL, triglicéridos, LDH, proteínas totales, albúmina, glucemia. Orina
8) Otros marcadores :17 B estradiol, testosterona libre y total, dímero-D, IL6, sCD14, sCD163 y niveles hormonales

6.6 Tratamiento Previo y Concomitante

Se les proveerá a las pacientes una lista de medicamentos que no podrán tomar durante el estudio (ver Anexo C). Serán avisadas de la prohibición de tomar cualquier medicación contraindicada.

Cualquier medicación (incluyendo medicación de venta libre como aspirina, antiácidos, vitaminas, suplementos minerales, preparaciones con hierbas) que la paciente esté recibiendo al momento del enrolamiento, o que reciba durante el estudio, deberá registrarse junto con los datos de administración y dosis.

6.7 Adherencia al Tratamiento

Todas las pacientes recibirán consejo acerca de la importancia de cumplir con la medicación. Las pacientes serán instruidas en cuanto a que deberán retornar los frascos al centro, tanto si están vacíos o con droga, desde la visita basal. Se contarán los comprimidos en cada visita para constatar la adherencia al tratamiento. Se realizará el recuento de medicación en cada visita para ser utilizado como indicador de adherencia. La adherencia será además medida mediante el cuestionario de adherencia de ACTG Modificado en cada visita según corresponda (ver Tabla 2).

7 Variables de Seguridad

La seguridad será evaluada por los eventos adversos, examen físico, signos vitales y datos de laboratorio.

7.1 Experiencias Adversas/Eventos Adversos

Durante todo el curso del estudio (que comienza desde la firma del Consentimiento Informado), el investigador vigilará cada paciente ante el eventual desarrollo de evento adverso (EA) clínico o de laboratorio. Un evento adverso se define como cualquier ocurrencia médica indeseable en la persona que participa en un estudio y que incluye aquellos eventos/experiencias que no necesariamente tienen relación causal con la droga del estudio. Previo a la administración de la droga, sólo los eventos adversos que coinciden con la definición de serios (Sección 7.4) y eventos adversos que el investigador considere que esté relacionado con el diseño del estudio y/o procedimientos deben registrarse.

Un evento adverso puede ser un síntoma, signo, o un hallazgo anormal en el laboratorio. Cualquier empeoramiento de una condición pre-existente o enfermedad intercurrente debe reportarse como evento/experiencia adverso. Un resultado de laboratorio anormal debe reportarse como un evento adverso si requiere tomar una conducta (por ejemplo, interrumpir o discontinuar la droga del estudio; o es necesario un tratamiento). La naturaleza del signo o síntoma adverso, la fecha y hora de inicio, duración y severidad, tratamiento indicado (si existiera) y la opinión del investigador de la causalidad acerca de la droga de estudio con una etiología alternativa, si fuera apropiado, debe documentarse. Para considerar un evento adverso intermitente o continuo, el mismo debe tener una naturaleza y severidad similar.

El investigador debe seguir todos los eventos adversos hasta su resolución clínica satisfactoria o el establecimiento de un estadio crónico estable luego de la finalización del estudio.

El investigador caracterizará la severidad del evento adverso de acuerdo con la siguiente definición:

Leve:	El evento adverso es transitorio y fácilmente tolerado por la paciente.
Moderado:	El evento adverso causa malestar en la paciente e interrumpe su actividad normal.
Severo:	El evento adverso causa una interferencia considerable con su actividad normal, y puede ser incapacitante o tener riesgo de vida.

El investigador deberá utilizar las siguientes definiciones para evaluar la relación del evento adverso con la droga del estudio:

Probablemente relacionado:	Un evento adverso que tiene una fuerte relación temporal con el uso de la droga del estudio o reaparece al reutilizar la droga y otra etiología resulta poco probable o significativamente menos probable.
Posiblemente relacionado:	Un evento adverso tiene una fuerte relación temporal con la droga de estudio y una etiología alternativa es igual o menos probable que la potencial relación con la droga.
Probablemente no relacionado:	Un evento adverso tiene poca o ninguna relación temporal con la droga de estudio y/o existe una etiología alternativa más probable.
No relacionado:	Un evento adverso se debe a una enfermedad o efecto de otra droga subyacente o concurrente y no se relaciona con la droga del estudio (no tiene relación temporal con la droga del estudio o existe una etiología alternativa mucho más probable).

7.2 Lipodistrofia

Redistribución de la grasa y apariencia cushingoide sin enfermedad de Cushing ha sido reportada como efecto observado en pacientes infectados con HIV/SIDA que reciben tratamiento antirretroviral incluyendo los inhibidores de la proteasa disponibles. Hasta hoy no se conocen los mecanismos ni las consecuencias a largo plazo de estos efectos. Esta condición fue agrupada bajo el título de “Lipodistrofia”. Sin embargo, hasta el presente no existe un consenso para su definición. Por ello, a fines de reportar los eventos adversos dentro del estudio, las siguientes condiciones deben ser reportadas como eventos discretos más que bajo el término de “lipodistrofia”:

a. Adelgazamiento de la grasa periférica (incluyendo cara, glúteos y caderas).
b. Adiposidad Central.
c. Ginecomastia.
d. Panículo adiposo dorsal (giba de búfalo).
e. Lipomas múltiples.

f. Apariencia cushingoide sin Enfermedad de Cushing.

Las anomalías metabólicas como hiperlipidemia o hiperglucemia no fueron observadas en forma consistente con anomalías de la distribución de grasa y no pueden ser etiquetadas como lipodistrofia a los efectos de reporte de evento adverso.

7.3 Reacción Alérgica

Las pacientes con reacción alérgica grado 1 o 2 pueden continuar en el Estudio, según decisión del Investigador. Se debe aconsejar a la paciente a que contacte inmediatamente al Investigador en caso de empeoramiento o si aparecen signos o síntomas sistémicos. Pueden indicarse antihistamínicos, corticosteroides tópicos o agentes anti pruriginosos.

Las pacientes que presenten reacción alérgica grado ≥3, que sea considerada posible o probablemente relacionada a las drogas de Estudio deben discontinuar permanentemente la medicación y ser retirados del Estudio. Las mismas serán medicadas según corresponda y serán apropiadamente seguidas hasta la resolución del EA.

7.4 Evento Adverso Serio

Los investigadores informarán al Sponsor, su Comités de Ética y la Agencia Regulatoria local, dentro de las 24 horas, de cualquier evento adverso serio reportado en el estudio. Un Evento Adverso Serio (*SAE*, de las siglas en inglés) es una experiencia adversa de la droga que resulta en cualquiera de las siguientes variables:

a. **Muerte.**
b. **Situación de riesgo de vida:** la paciente estuvo en riesgo de morir al momento del evento/experiencia adverso. Ello no se refiere a los riesgos de muerte hipotéticos si el EA fue más severo o progresó.

c. **Paciente hospitalizado o prolongación de la hospitalización si la misma existe.**
d. **Incapacidad/Discapacidad persistente o significativa** — Cualquier EA que tenga como resultado una interrupción sustancial de alguna habilidad para continuar con funciones de la vida normal, incluyendo capacidad de trabajar. Esto no intenta incluir interrupciones transitorias de las actividades diarias.
e. **Eventos/Experiencias médicas importantes** que no resulten en muerte, tengan riesgo de vida, o requieran hospitalización pueden considerarse eventos adversos serios, basados en el criterio médico, **podrían poner en riesgo a la paciente o requerir intervención médica o quirúrgica para prevenir algunos de los resultados listados abajo,** por ejemplo, muerte, evento con riesgo de vida, hospitalización o prolongación de hospitalización previa, incapacidad/discapacidad persistente o significativa. Ejemplos de tales eventos/experiencias médicos/as incluyen: broncoespasmo alérgico que requiera tratamiento intensivo en emergencias o en su hogar, discrasias sanguíneas o convulsiones que no resulten en hospitalización de la paciente, o desarrollen dependencia a droga o abuso de drogas.

Nota: Evento/Experiencia adverso severo no necesariamente es serio; el término severo es una medida de intensidad mientras, que un evento adverso serio está basado en los criterios regulatorios mencionados con anterioridad.

El período de observación para el reporte de eventos adversos (AE) y eventos adversos serios (EAS) para una paciente individual comienza en la Visita de Selección (después que la paciente haya firmado el formulario de consentimiento informado) y se extiende hasta la Visita Final (visita de seguimiento a las 4 semanas post-tratamiento) o hasta 4 semanas posteriores a la última dosis de tratamiento, lo que ocurra más tarde, para documentar eventos adversos tardíos.
Si el Investigador toma conocimiento de un Evento Adverso Serio relacionado a la medicación del Estudio luego de la Visita Final, deberá completar un formulario de reporte de EAS. Si el sitio de Investigación ya fue cerrado, se deberá contactar al Monitor Médico del Sponsor o a la persona que él designe.

7.4.1 Tabla 3: Tiempos para el reporte de EAS

	**Reporte Inicial**		**Reporte de Seguimiento**	
**Tipo de Evento**	**Tiempo de Reporte**	**Documentos**	**Tiempo de Reporte**	**Documentos**
Todos los EAS	24 horas	Formulario de reporte inicial de EAS	24 horas	Formulario de reporte de seguimiento de EAS
ALT ≥3 x ULN más bilirrubina ≥2 x ULN (35% directa)	24 horas	Formulario de reporte inicial de EAS	24 horas	Formulario de reporte de seguimiento de EAS
ALT ≥5 x ULN que persiste ≥2 semanas	24 horas	Formulario de reporte inicial de EAS	24 horas	Formulario de reporte de seguimiento de EAS
ALT ≥8 x ULN	24 horas	Formulario de reporte inicial de EAS	24 horas	Formulario de reporte de seguimiento de EAS
ALT ≥3 x ULN o ≥3 veces de incremento comparado al basal con aparición o empeoramiento de síntomas de hepatitis o hipersensibilidad	24 horas	Formulario de reporte inicial de EAS	24 horas	Formulario de reporte de seguimiento de EAS

7.5 Ideas o Conductas Suicidas

Si una paciente experimenta un evento adverso posiblemente relacionado a suicidalidad (*PSRAE* por sus siglas en inglés), mientras participa del Estudio, y el investigador considera que cumple con la definición de serio de acuerdo con la Conferencia Internacional de Armonización (*ICH* por sus siglas en inglés)-EA 2, el investigador debe completar un formulario de EAS correspondiente. Un PSRAE puede incluir, pero no se limita sólo a estos eventos: eventos que involucran ideación suicida, preparación de un acto inminentemente suicida, intento de suicidio o suicidio. El investigador debe utilizar su juicio para definir si el evento es posiblemente relacionado a intento de suicidio.

7.6 Manejo de Toxicidad

Con el propósito de manejo médico, todos los eventos adversos y anomalías en el laboratorio que ocurran durante el estudio deberán ser evaluados por el investigador. El Anexo B contiene “Grados de Toxicidad Clínica”. Esta tabla debe utilizarse para graduar los eventos adversos. Todos los eventos adversos y alteraciones del laboratorio se seguirán hasta su resolución clínica satisfactoria o el establecimiento de un estadio crónico estable luego de la finalización del estudio. Las siguientes guías deben utilizarse para el manejo de la toxicidad relacionada a la droga del estudio en todas las pacientes.

7.6.1 Grados 1-2

Para elevaciones de ALT, AST y bilirrubina ver Sección 7.7.
Las pacientes que presenten un evento adverso o anormalidad de laboratorio de Grado 1 o 2 relacionado con la droga en estudio podrán continuar con las medicaciones del estudio.

7.6.2 Grados 3-4

Las pacientes que presenten un evento adverso o anormalidad de laboratorio de Grado 3 ó 4 deberán interrumpir toda medicación del estudio. Una vez resuelto el evento adverso o anormalidad de laboratorio hasta ubicarse a 1 Grado del nivel basal de la paciente (sin exceder el Grado 2), la paciente podrá reanudar el régimen bajo la supervisión del investigador. Si posteriormente la paciente presentara recurrencia de un evento adverso o anormalidad de laboratorio de Grado 3 ó 4 relacionado con el tratamiento, deberá interrumpir nuevamente el régimen asignado y reanudarlo cuando el evento adverso o anormalidad de laboratorio se haya ubicado a 1 Grado del valor basal de la paciente (sin exceder el Grado 2).
Si el evento adverso o anormalidad de laboratorio manifestado por la paciente no se ubicara a 1 Grado de su nivel basal (sin exceder el Grado 2) dentro de las ocho

semanas de la interrupción de la medicación en estudio, la paciente deberá ser retirada del estudio.

Para elevaciones de ALT, AST y bilirrubina ver Sección 7.7., para hiperglucemia ver Sección 7.8., para amilasa pancreática ver Sección 7.9.

7.7 Reglas de Toxicidad Hepática para Discontinuación del Tratamiento

Se han diseñado las siguientes reglas para mantener la seguridad de las pacientes y para evaluar la posibilidad de daño hepático, durante la administración de las drogas en estudio y el seguimiento posterior a su interrupción.

El tratamiento en estudio debe ser interrumpido en las siguientes situaciones:

a. ALT ≥3 x ULN y bilirrubina ≥2 x ULN (35 % bilirrubina directa; se requiere bilirrubina fraccionada);
b. ALT ≥8 x ULN;
c. ALT ≥3 x ULN (si la ALT basal es menor ULN) con signos o empeoramiento de hepatitis aguda o hipersensibilidad como fatiga, náuseas, vómitos, dolor en cuadrante superior derecho, fiebre, rash o eosinofilia o;
d. ALT ≥3 x ULN en la visita basal con signos o empeoramiento de hepatitis aguda o hipersensibilidad como fatiga, náuseas, vómitos, dolor en cuadrante superior derecho o fiebre, rash o eosinofilia o;
e. ALT ≥5 ULN y <8 x ULN que persiste por más de 2 semanas (con bilirrubina <2 x ULN y sin signos ni síntomas de hepatitis aguda o de hipersensibilidad);
f. ALT ≥5 x ULN pero <8 x ULN y que no puede ser monitoreada semanalmente por dos semanas.

Las pacientes que presenten ALT ≥5 x ULN deben ser seguidas semanalmente hasta la resolución o estabilización (ALT <5 x ULN en dos evaluaciones consecutivas).

Cuando se cumpla una regla de detención de tratamiento por toxicidad hepática, la paciente deberá ser discontinuada inmediatamente del estudio.

No se debe restaurar DTG por el riesgo de hipersensibilidad.

a. El evento debe ser reportado al Sponsor dentro de las 24 horas de conocido el mismo;
b. Se debe completar el CRF o el formulario de reporte de EAS según aplique. Se debe reportar si se han realizado estudios de imágenes o biopsia hepática;
c. El evento debe ser seguido hasta que los valores se resuelvan, se estabilicen o vuelvan al nivel basal;
d. Se deben realizar todos los esfuerzos necesarios para que la paciente retorne al sitio de investigación para repetir la química hepática dentro de las 24 horas, realizar el seguimiento del evento y el monitoreo cercano;
e. Se recomienda la evaluación por un especialista o un hepatólogo;
f. Se deben realizar controles dos veces por semana hasta que la química hepática (ALT, AST, fosfatasa alcalina, bilirrubina) se resuelva, se estabilice, o retornen los valores al nivel basal.

Se deben considerar también los siguientes tests para evaluar la etiología del evento:

a. Serologías virales para hepatitis incluyendo:
   - Anticuerpo IgM para Hepatitis A;
   - HBsAg y anticore IgM para Hepatitis B;
   - Hepatitis C RNA;
   - Anticuerpo IgM para Hepatitis E;
b. Anticuerpo IgM para Citomegalovirus;
c. Anticuerpo IgM para la cápside viral de Epstein Barr (si no está disponible se pueden dosar anticuerpos heterófilos o monospot);
d. Screening de sífilis;
e. Drogas de abuso incluyendo alcohol;
f. Test de acetaminofeno (APAP test);
g. CPK y LDH;
h. Bilirrubina fraccionada si la bilirrubina total es mayor de 1,5 x ULN;
i. Hemograma completo para evaluar recuento de eosinófilos;
j. Anticuerpo antinúcleo, antimúsculo liso y anticuerpos hígado-riñón microsomal tipo 1;

k. Diagnóstico por imágenes hepáticas (ecografía, RMN, TAC) para evaluar daño hepático agudo.

Reportar empeoramiento de hepatitis aguda o hipersensibilidad como fatiga, disminución del apetito, náuseas, vómitos, dolor en cuadrante superior derecho, ictericia, fiebre o rash en la hoja de EA.
Reportar el uso de medicaciones concomitantes, acetaminofeno, remedios herbáticos, otros de venta libre o probables hepatotóxicos, en la hoja de reporte de medicaciones concomitantes.

7.8 Hiperglucemia

Pacientes que experimenten elevación de la glucemia relacionada a la droga del estudio de Grado 3 o 4 podrán continuar con la medicación del estudio, teniendo en cuenta la provisión de un manejo adecuado de la hiperglucemia en el momento apropiado. Se deberá obtener un valor de glucemia en ayunas dentro de las 2 semanas del primer valor de hiperglucemia Grado 3 o 4. La hiperglucemia se podrá manejar con agentes hipoglucemiantes orales o insulina, de acuerdo a lo que considere apropiado el investigador.

7.9 Pancreatitis/Amilasa Pancreática Grado 3-4

El diagnóstico de pancreatitis debe considerarse si los síntomas clínicos de náuseas, vómitos o dolor abdominal están presentes. Las pacientes con estos signos y síntomas deben ser evaluadas en forma apropiada ante la presencia de pancreatitis utilizando los métodos diagnósticos que incluyan amilasa y lipasa sérica y/o diagnóstico por imágenes abdominales por ultrasonido o tomografía computada. Si se confirma el diagnóstico de pancreatitis, todas las drogas del régimen deben interrumpirse inmediatamente.
Si durante el curso del estudio se evidencia una elevación de la amilasa pancreática Grado 3-4 y se sospecha pancreatitis (pacientes HIV-1 pueden presentar elevaciones de amilasa sin ser necesariamente de origen pancreático), deberá interrumpirse la

medicación del estudio en forma inmediata. La paciente deberá ser evaluada en busca de signos y síntomas de pancreatitis y deberán realizarse evaluaciones diagnósticas adicionales, según sea lo clínicamente indicado.

Si se confirma el diagnóstico de pancreatitis, debe iniciarse un tratamiento apropiado. El investigador no deberá reiniciar el tratamiento antirretroviral hasta que los signos y síntomas de la pancreatitis se hayan resuelto y los valores de la amilasa hayan alcanzado valores normales.

7.10 Eventos Clínicos Relacionados con HIV

Las pacientes con HIV-1 que participan en estudios clínicos pueden desarrollar enfermedades típicamente asociadas con el SIDA. Dentro de estas afecciones se pueden citar ciertos carcinomas, infecciones oportunistas, disfunción neurológica y algunos síntomas constitucionales generalizados. El Anexo D contiene una lista de las manifestaciones esperables relacionadas con la infección por HIV. Los eventos que se enumeran en el Anexo D son Eventos Relacionados al HIV, y no se considerarán Eventos Adversos.

Se utilizará el mismo formulario para registrar los eventos adversos y los eventos relacionados con el HIV. Los eventos relacionados con el HIV que sean de naturaleza seria, según definición en la sección 7.4, deberán registrarse de la misma manera que los eventos adversos serios.

8 Consideraciones Estadísticas

8.1 Plan de Análisis Estadístico

La retención en la atención de salud, en el tratamiento y la supresión virológica se mostrará como proporción de las pacientes incluidas en el estudio que recibieron al menos una dosis de la medicación del estudio (análisis por intención de tratar modificado). El análisis preliminar incluirá la evaluación de los resultados en semana 24 y el análisis final de los resultados en la semana 48 para las 60 pacientes.

La estadística descriptiva será utilizada para evaluar los resultados de los objetivos primarios y secundarios. Los resultados serán informados en valores de mediana, media, desvío estándar y rangos interquartiles (IQR) o frecuencias expresadas en porcentajes (%) y por intervalo de confianza 95% según corresponda. La significancia global de los cambios observados en cada visita serán evaluados mediante un ANOVA de medidas repetidas, una prueba no paramétrica de Friedman o un test de Chi-cuadrado para tendencias, según corresponda. Si se obtuvieran resultados significativos, en cada punto o semana de evaluación las diferencias longitudinales entre la visita basal y la visita de medición serán determinadas usando la prueba de t de student o el test de Wilcoxon rank-sum para las muestras pareadas, según sea necesario. Se utilizará corrección por Bonferroni para los análisis post-hoc. Un valor de *p* con dos colas de <0,05 será considerado estadísticamente significativo. Las caracteristicas del basal y las visitas de seguimiento serán comparadas usando los tests de Wilcoxon rank-sum o test de Fisher/Chi cuadrado.

Los cambios en los valores de CD4 se medirán como la ganancia en números de células, tomando como punto de partida los valores de la visita basal. Para la resistencia, la estadística será descriptiva, determinada por la presencia de mutaciones como la M184V y otras mutaciones para los INTI en cualquier momento. La emergencia de cepas resistentes se describirá solamente para aquellas pacientes que experimenten fallo en el tratamiento. La presencia de mutaciones resistentes se describirá como porcentaje de pacientes con fallo en el tratamiento que presenten la mutación de resistencia de HIV.

Todo evento adverso será evaluado, resumido y descrito como la proporción de pacientes que presenten el evento, y el número total de eventos. La tabla de eventos adversos DAIDS AE (Anexo B) será utilizada para clasificar el grado y también será usada para los valores de laboratorio.

La seguridad se evaluará por incidencia de eventos. Se utilizará un intervalo de confianza del 95% para evaluar significancia estadística y precisión del riesgo relativo estimado. Todos los eventos se describirán utilizando el MedDRA o Sistema de clasificación de la Organización Mundial de la Salud (OMS).

Cambios en la media de los valores de laboratorio clínico y signos vitales obtenidos en el transcurso del tiempo se evaluarán utilizando el test de Student o test de Mann – Whitney para muestras independientes, segun corresponda.

8.1.1 Nivel de Significancia

Todos los test estadísticos se considerarán significativos si el nivel alfa es menor al 5%. Sin embargo, niveles alfa menores a 10% se los considerará indicativos de tendencias.

8.1.2 Procedimiento para el Manejo de Datos Perdidos

No se imputarán o reemplazarán datos perdidos. Se realizarán observaciones en el momento del último seguimiento.

8.1.3 Procedimiento para el Reporte de Desvíos del Plan Estadístico Original

Cualquier desvío será reportado como enmienda al plan de análisis estadístico.

8.1.4 Criterio de Selección de Pacientes que Deben ser Incluidas en el Análisis

El primer análisis se realizará acorde a Intención de Tratar (del inglés, *Intent to Treat-exposed, ITT-e*), cuyo principio está basado en que todas las pacientes enroladas en el estudio, y que hayan tomado al menos una dosis de la medicación del estudio serán analizadas**.**

9 Acceso Directo al Dato Fuente/Documentos

Cuando sea requerido por las autoridades regulatorias, el Comité de Ética Independiente, o los auditores, los investigadores permitirán el acceso directo a todos los documentos relacionados con el estudio que se hayan solicitado.

10 Conducción Ética del Estudio

10.1 Consentimiento Informado

Es responsabilidad del investigador asegurarse de explicar en forma adecuada los objetivos, métodos, beneficios anticipados y riesgos potenciales de este estudio y obtener, con firma y fecha y voluntariamente, el consentimiento informado, aprobado por el Comité de Ética Independiente, en forma prioritaria para poder participar del protocolo. El investigador también explicará a la paciente que tiene el derecho de rehusarse a participar del estudio o que puede abandonar el mismo en cualquier momento por cualquier motivo.
El investigador documentará en la historia clínica de la paciente que el consentimiento informado se obtuvo previo a la realización de cualquier procedimiento relacionado con el estudio y se archivará un original del consentimiento informado con los documentos del estudio. Un original firmado y fechado en el mismo momento será entregado a la paciente. El Anexo H contiene elementos del Consentimiento Informado.

10.2 Conducción del Estudio

El estudio será conducido de acuerdo con el protocolo y las normas de buenas prácticas clínicas, aplicando las regulaciones y guías de investigaciones clínicas, y todas las regulaciones locales aplicables.

11 Manejo y Almacenamiento de Datos Obtenidos

Formulario de Registro Clínico Individual (de las siglas en inglés, *CRF*) será utilizado para almacenar toda la información recogida durante el estudio. El CRF se completará para cada paciente enrolada en este estudio. Toda la información escrita en el CRF reflejará el documento fuente de la paciente.

Cada paciente enrolada en este estudio tendrá una historia clínica propia que recopilará toda la información relacionada con la salud de la paciente. Cualquier corrección necesaria se realizará trazando una línea simple sobre el dato incorrecto y escribiendo en la revisión, y firmando y fechando el investigador o la persona que esté delegada. Los datos no podrán ser tachados o borrados, ni se utilizarán líquidos correctores. Si la razón de la corrección no es obvia, se deberá acompañar el cambio con una breve explicación (por ejemplo, error en la transcripción). Toda la información escrita en el CRF también se verá reflejada en los documentos de origen de la paciente.

12 Garantía de la Calidad de los Datos

En orden de mantener la integridad de los datos del estudio, la información recogida en los CRF y resultados de laboratorio serán verificados por el investigador. Esto será documentado con la firma del investigador.

El investigador llevará un formulario de selección/enrolamiento y una completa identificación de cada paciente con la finalidad de hacer el seguimiento a largo plazo si fuera necesario.

13 Anexos

13.1 Anexo A. Sinopsis del Protocolo

**Título del Protocolo**	Tratamiento con Dolutegravir asociado a Tenofovir/Lamivudina o Emtricitabina en mujeres trans infectadas con HIV: estudio piloto
**Objetivos del Estudio**	El objetivo primario de este estudio piloto es determinar la retención en la atención de salud de las mujeres trans bajo tratamiento con DTG-TDF-FTC o 3TC.<br>Los objetivos secundarios:<br>a. Evaluar la eficacia del régimen antirretroviral a las 48 semanas;<br>b. Describir la seguridad y tolerabilidad de este régimen;<br>c. Evaluar la adherencia a través de 48 semanas;<br>d. Determinar la satisfacción de la paciente con este régimen;<br>e. Identificar los factores individuales, sociales y contextuales asociados con la adherencia y retención.
**Variables Primarias de Evaluación**	El objetivo principal será evaluado a través de la proporción de individuos que proporcionen información sobre el uso de TAR al final del estudio:<br>a. Retención en la atención: Proporción de personas inscriptas y tratadas que brinden información clínica hasta las 48 semanas de seguimiento.<br>b. Retención en el tratamiento: Proporción de personas inscriptas y tratadas que reciben los fármacos del estudio hasta 48 semanas de seguimiento.
**Variables Secundarias de Evaluación**	Los objetivos secundarios se evaluarán utilizando los siguientes criterios de valoración:<br>a. Proporción de pacientes con niveles de ARN-HIV menores a 50 copias/mL en un análisis de intención de tratar a la

semana 48 utilizando el algoritmo *Snapshot* de la FDA (dato faltante, cambio o discontinuación de tratamiento son considerados fallo) en las pacientes que al menos hayan tomado una dosis del medicamento;

b. Frecuencia, tipo y gravedad de los eventos adversos y alteraciones del laboratorio;
c. Recuento de pastillas, escala analógica visual para la adhesión en cada visita;
d. Cambios en las puntuaciones de las escalas de estigma y discriminación, calidad de vida, apoyo social, ansiedad y depresión (Berger, WBI, DUKE, CES-D, STAI por sus siglas en inglés) al inicio del estudio y en las semanas 4, 24 y 48 de seguimiento;
e. Cambios en las puntuaciones de las escalas de comportamiento sexual, uso de drogas y alcohol al inicio de estudio y en cada visita de seguimiento;
f. Asociación de características basales individuales, sociales y contextuales con el porcentaje de adherencia y retención a las 48 semanas.

**Población**	Pacientes HIV-1 positivas, ≥18 años de edad, sin tratamiento previo, auto-identificadas como mujeres trans que cumplan con todos los criterios de inclusión y ninguno de los criterios de exclusión.
**Diseño del Estudio**	Ensayo prospectivo, abierto, de una sola rama con DTG-TDF-FTC o 3TC en pacientes HIV positivas naïve de tratamiento.
**Regímenes**	Dolutegravir 50 mg más la co-formulación lamivudina 300 mg o emtricitabina 200 mg/ tenofovir 300 mg una vez al día con alimentos.
**Duración**	48 semanas
**Tamaño de la muestra**	60 pacientes

13.2 Anexo B. Toxicidad Clínica

DAIDS AE Grading Table Version 2.0- November 2014

PARÁMETRO	TOXICIDAD GRADO 1 LEVE	TOXICIDAD GRADO 2 MODERADO	TOXICIDAD GRADO 3 SEVERA	TOXICIDAD GRADO 4 CON RIESGO DE VIDA
Evento clínico no identificado en ningún otro lugar de estas tablas	Síntomas leves que no interfieren o causan leve interferencia con las actividades sociales y funcionales habituales sin necesidad de intervención alguna	Síntomas moderados que causan más que una mínima interferencia con las actividades sociales y funcionales habituales y requieren alguna intervención	Síntomas severos que provocan incapacidad para realizar las actividades sociales y funcionales habituales y requieren alguna intervención u hospitalización	Síntomas **con riesgo de vida** que provocan incapacidad para realizar las actividades básicas diarias y requieren alguna intervención para prevenir daño permanente , secuela o muerte

CONDICIONES CLÍNICAS MAYORES

CARDIOVASCULAR

PARÁMETRO	TOXICIDAD GRADO 1 LEVE	TOXICIDAD GRADO 2 MODERADO	TOXICIDAD GRADO 3 SEVERA	TOXICIDAD GRADO 4 CON RIESGO DE VIDA
ARRITMIA	Asintomática. No requiere intervención-	Asintomático, requiere intervención no urgente	Sin síntomas de riesgo de vida y requiere intervención no urgente	Arritmia con riesgo de vida o que requiere intervención urgente
Trastornos de TA [1]<br><br>HIPERTENSIÓN (la menor de varias lecturas tomadas durante la visita)<br><br>(≥ 18 años)	140 a <160 mmHg de TA sistólica<br><br>o<br><br>90 a <100 mmHg de diastólica	≥160 a <180 mmHg de TA sistólica<br><br>o<br><br>≥100 a <110 mmHg de diastólica	≥180 mmHg de TA sistólica<br><br>o<br><br>≥110 mmHg de diastólica	Riesgo de vida por evento hipertensivo en paciente sin diagnóstico previo de HTA (ej. : HTA maligna) o que requiere hospitalización
HIPERTENSIÓN (< 18 años)	>120/80mmHg	≥95 a <99 percentil + 5 mmHg ajustado por edad, altura y género (sistólica y/o diastólica)	≥99 percentil + 5 mmHg ajustado por edad, altura y género (sistólica y/o diastólica)	Riesgo de vida en un participante sin diagnóstico previo de hipertensión (ej.: hipertensión maligna) o que requiere hospitalización
HIPOTENSION	asintomática	Sintomática , corregible con líquidos por vía oral	Sintomática requiere fluidos IV	Shock que requiere uso de vasopresores o asistencia mecánica para mantener los niveles de TA
	NA	NA	Nuevos síntomas	Angina inestable

ISQUEMIA CARDÍACA O INFARTO **(** reportar uno solo **)**			(angina estable) o tests nuevos que diagnostiquen isquemia	o Infarto Agudo de Miocardio
INSUFICIENCIA CARDÍACA	Asintomática y anormalidades de laboratorio o imágenes	Síntomas con actividad o esfuerzo leves a moderados	Síntomas en reposo o con actividad mínima (ej. : hipoxemia) o que requieran intervención (ej.: oxígeno)	Riesgo de vida o indicación de intervención urgente (ej. : medicaciones vasoactivas, dispositivos cardíacos o trasplante)
HEMORRAGIA, (con pérdida aguda significativa)	NA	Sintomática , Y sin requerimiento de transfusión	Sintomática Y requiere transfusión < 2 unid.	Hipotensión con riesgo de vida o que requiere transfusión >2 unid (para niños > 10 cc/ Kg)
PROLONGACIÓN DEL INTREVALO PR O BLOQUEO AV **(** reportar uno solo **)** *> 16 años de edad*	intervalo PR 0.21 a < 0.25 segundos	intervalo PR ≥ 0.25 segundos o Bloqueo AV de segundo grado Tipo I	Bloqueo AV de segundo grado Tipo II o pausa ventricular ≥ 3.0 segundos	Bloqueo AV completo
<16 años	Bloqueo AV de primer grado (intervalo PR > que el normal para edad y frecuencia cardíaca)	Bloqueo AV de segundo grado Tipo I	Bloqueo AV de segundo grado Tipo II o pausa ventricular ≥ 3.0 segundos	Bloqueo AV completo
PROLONGACIÓN DEL INTREVALO QTC	0.45 a 0.47 segundos	> 0.47a 0.50 segundos	> 0.50 segundos o ≥ 0.06 segundos que el basal	Riesgo de vida (ej.: Torsada de punta u otras arritmias ventriculares graves)
TROMBOSIS O EMBOLISMO **(**reportar uno solo**)**	NA	Síntomas que no requieren intervención	Síntomas que requieren intervención	Evento embólico con riesgo de vida (ej.: tromboembolismo pulmonar)

1- Valores de referencia de TA en < 18 años ver : Expert Panel on Integrated Guidelines for Cardiovascular Health and Risk Reduction in Children and Adolescents. *Pediatrics* 2011;128;S213; originally published online November 14, 2011; DOI: 10.1542/peds.2009-2107C
2- Según fórmula de Bazett's.

Dermatológicas

PARÁMETRO	**TOXICIDAD GRADO 1 LEVE**	**TOXICIDAD GRADO 2 MODERADO**	**TOXICIDAD GRADO 3 SEVERA**	**TOXICIDAD GRADO 4 CON RIESGO DE VIDA**
ALOPECÍA (solamente cuero cabelludo)	Detectado por el participante o el cuidador o el médico Y no interfieren o causa leve interferencia con las actividades sociales y funcionales habituales	Obvio en inspección visual Y causando más que una mínima interferencia con las actividades sociales y funcionales habituales	NA	NA
HEMATOMA	Localizado en una sola área	Localizado en más de una sola área	Generalizado	NA
CELULITIS	NA	Tratamiento no parenteral (ej.: antibióticos, antifúngicos o antivirales orales)	Indicación de tratamientos endovenosos	Consecuencias con riesgo de vida (ej. : sepsis, necrosis tisular)
HIPERPIGMENTACIÓN	Leve o localizada ,no interfiere o causa leve interferencia con las actividades sociales y funcionales habituales	Marcada o generalizada causando una interferencia más que mínima con las actividades sociales y funcionales habituales	NA	NA
HIPOPIGMENTACIÓN	Leve o localizada, no interfiere o causa leve interferencia con las actividades sociales y funcionales habituales	Marcada o generalizada causando una interferencia más que mínima con las actividades sociales y funcionales habituales	NA	NA
PETEQUIAS	Localizado en una sola área	Localizado en más de una sola área	Generalizado	NA
PRURITO (sin lesiones cutáneas) [3]	Picazón no interfiere o causa leve interferencia con las actividades sociales y funcionales habituales	Picazón causando más que una mínima interferencia con las actividades sociales y funcionales habituales	Picazón causando incapacidad para realizar las actividades sociales y funcionales habituales	NA
	Localizado	Difuso O	Difuso Y	Extenso o bullas

RASH<br>*Especificar tipo, si aplica*		lesiones diana	vesículas o número limitado de bullas o ulceraciones superficiales en las mucosas limitado a un sitio)	generalizadas O ulceraciones en dos o más mucosas O Stevens-Johnson O epidermonecrosis tóxica

3-Para prurito asociado a sitios de inyección o infusión ver Reacciones en sitios de inyección o infusión

Endocrinológicas

PARÁMETRO	TOXICIDAD GRADO 1 LEVE	TOXICIDAD GRADO 2 MODERADO	TOXICIDAD GRADO 3 SEVERA	TOXICIDAD GRADO 4 CON RIESGO DE VIDA
DIABETES MELLITUS	Controlado sin medicación	Controlado con medicación O modificación de régimen actual	No controlada a pesar de modificación del tratamiento O internación para control urgente de la glucemia	Complicaciones con riesgo de vida (ej.: cetoacidosis, coma hiperosmolar y falla orgánica múltiple)
GINECOMASTIA	Detectada por el paciente, cuidador o el médico Y que causa ninguna o una mínima interferencia con sus actividades sociales y funcionales habituales	Evidente al examen visual físico Y que causa dolor con más que mínima interferencia con sus actividades sociales y funcionales habituales	Cambios desfigurantes Y síntomas que requieren una intervención o que causan discapacidad para realizar actividades funcionales y sociales habituales	NA
HIPERTIROIDISMO	Asimtomatico Y Valores de laboratorio anormales	Síntomas que causan mínima a moderada interferencia con las actividades sociales y funcionales habituales O indicación de tratamiento de supresión tiroidea	Síntomas que causan incapacidad de realizar las actividades sociales y funcionales habituales O enfermedad no controlada a pesar de la modificación de tratamiento	Complicaciones con riesgo de vida (ej.: tormenta tiroidea)
HIPOTIROIDISMO	Asintomático Y valores de laboratorio anormales	Síntomas que causan mínima a moderada interferencia con las actividades sociales y funcionales habituales O	Síntomas que causan incapacidad de realizar las actividades sociales y funcionales habituales O	Complicaciones con riesgo de vida(ej.: mixedema, coma)

		indicación de tratamiento de supresión tiroidea	enfermedad no controlada a pesar de la modificación de tratamiento	
LIPOATROFIA[4]	Detectada por el paciente, cuidador o el médico Y que causa ninguna o una mínima interferencia con sus actividades sociales y funcionales habituales	Evidente al examen visual físico Y que causa dolor con mínima interferencia con sus actividades sociales y funcionales habituales	Cambios desfigurantes	NA
LIPOHIPERTROFIA[5]	Detectada por el paciente, cuidador o el médico Y que causa ninguna o una mínima interferencia con sus actividades sociales y funcionales habituales	Evidente al examen visual físico Y que causa dolor con mínima interferencia con sus actividades sociales y funcionales habituales	Cambios desfigurantes	NA

4-Definición: Trastorno que consiste en la pérdida de tejido adiposo en el rostro, las extremidades y los glúteos.
5- Definición: Trastorno que consiste en la acumulación anormal de tejido adiposo en el cuello, el pecho y el abdomen.

Gastrointestinal

PARÁMETRO	**TOXICIDAD GRADO 1 LEVE**	**TOXICIDAD GRADO 2 MODERADO**	**TOXICIDAD GRADO 3 SEVERA**	**TOXICIDAD GRADO 4 CON RIESGO DE VIDA**
ANOREXIA	Pérdida de apetito sin disminución de alimentación	Pérdida de apetito asociado a disminución de la ingesta oral sin pérdida de peso	Pérdida de apetito asociado a pérdida significativa de peso	Complicaciones con riesgo de vida O necesidad de una intervención (alimentación enteral, parenteral, etc.)
ASCITIS	Sintomático	Síntomas E indicación de una intervención (ej.: diuréticos, paracentesis)	Síntomas persisten o recidivan a pesar de una intervención	Complicaciones con riesgo de vida
HINCHAZÓN O DISTENSIÓN *Reportar solo una*	Síntomas que causan ninguna o una mínima interferencia con sus actividades sociales y funcionales habituales	Síntomas que causan moderada interferencia con sus actividades sociales y funcionales habituales	Síntomas que causan incapacidad de realizar sus actividades sociales y funcionales habituales	NA
COLECISTITIS	NA	Síntomas E intervención médica	Intervención endoscópica o quirúrgica indicada	Complicaciones con riesgo de vida (ej. sepsis,

		indicada		perforación)
CONSTIPACIÓN	NA	Constipación persistente que requiere uso regular de modificaciones de dieta , laxantes o enemas	Constipación que requiere evacuación manual	Complicaciones con riesgo de vida (ej. obstrucción)
DIARREA<br>*≥ 1 año de edad*	Episodios intermitentes o transitorios de heces no formadas O aumento de ≤ 3 deposiciones en un período de 24 hs.	Episodio persistente de heces no formadas o líquidas<br>O<br>aumento de 4 a 6 deposiciones en un período de 24 hs.	Aumento de ≥ 7 deposiciones en un período de 24horas O indicación de reposición intravenosa de fluidos	Complicaciones con riesgo de vida (ej. shock hipovolémico).
< 1 año de edad	Heces líquidas (menos formadas de lo habitual) pero en cantidad habitual	Heces líquidas en mayor cantidad de lo habitual O deshidratación leve	Heces líquidas con deshidratación moderada	Complicaciones con riesgo de vida (ej. heces líquidas que resultan en deshidratación severa, shock hipovolémico)
Disfagia u Odinofagia<br>*Reportar una sola y especificar su ubicación*	Síntomas que permiten mantener la dieta habitual	Síntomas que provocan alteración dietaria sin indicación de intervención	Síntomas que provocan alteración dietaria severa con indicación de intervención	Reducción de la ingesta de alimentos que resulta en riesgo de vida
SANGRADO GASTROINTESTINAL	Que requiere suplemento de hierro como única intervención	Indicación de intervención endoscópica	Indicación de transfusión	Complicaciones con riesgo de vida (ej.: shock hipovolémico)
MUCOSITIS O ESTOMATITIS<br>*Reportar una sola y especificar su ubicación*	Eritema mucoso	Pseudomembranas y úlceras en parches	Pseudomembranas o úlceras confluentes O sangrado de la mucosa con trauma menor	Complicaciones con riesgo de vida (ej. aspiración, asfixia) O necrosis tisular O sangrado extenso espontáneo de la mucosa
NÁUSEA	Transitoria (<24 hs.) o intermitente Y que provoca ninguna o una mínima interferencia con la ingesta oral	Náusea persistente que resulta en una disminución de la ingesta oral durante entre 24 y 48 horas	Náusea persistente que resulta en una ingesta oral mínima por > 48 hs. O indicación de rehidratación (ej. fluidos intravenosos)	Complicaciones con riesgo de vida (ej.: shock hipovolémico)
PANCREATITIS	NA	Sintomática, sin indicación de internación	Sintomática, con indicación de internación	Complicaciones con riesgo de vida (ej. insuficiencia circulatoria, hemorragia, sepsis)
PERFORACIÓN (colon o recto)	NA	NA	Indicación de intervención	Complicaciones con riesgo de vida
PROCTITIS	Malestar rectal sin indicación de intervención	Síntomas que provocan una interferencia más que mínima con sus actividades sociales y	Síntomas que impiden realizar sus actividades sociales y funcionales habituales O	Complicaciones con riesgo de vida (ej. perforación)

		funcionales habituales O indicación de intervención médica	indicación de intervención quirúrgica	
DESCARGA ANAL	Descarga visible	Descarga que requiere el uso de toallitas	NA	NA
VÓMITOS	Transitorios o intermitentes Y que provocan ninguna o una mínima interferencia con la ingesta oral	Episodios frecuentes que no provocan deshidratación o provocan deshidratación leve	Vómitos persistentes que resultan en hipotensión ortostática O indicación de rehidratación (ej. fluidos intravenosos)	Complicaciones con riesgo de vida (ej.: shock hipovolémico)

Musculoesquelético

PARÁMETRO	**TOXICIDAD GRADO 1 LEVE**	**TOXICIDAD GRADO 2 MODERADO**	**TOXICIDAD GRADO 3 SEVERA**	**TOXICIDAD GRADO 4 CON RIESGO DE VIDA**
ARTRALGIA	Dolor articular que provoca ninguna o una mínima interferencia con sus actividades sociales y funcionales habituales	Dolor articular que provoca una interferencia más que mínima con sus actividades sociales y funcionales habituales	Dolor articular que impide realizar sus actividades sociales y funcionales habituales	Dolor articular incapacitante que impide realizar las actividades diarias básicas
ARTRITIS	Rigidez o inflamación articular que provoca ninguna o una mínima interferencia con sus actividades sociales y funcionales habituales	Rigidez o inflamación articular que provoca una interferencia más que mínima con sus actividades sociales y funcionales habituales	Rigidez o inflamación articular que impide realizar sus actividades sociales y funcionales habituales	Rigidez o inflamación articular incapacitante que impide realizar las actividades diarias básicas
MIALGIA (generalizada)	Dolor muscular que provoca ninguna o una mínima interferencia con sus actividades sociales y funcionales habituales	Dolor muscular que provoca una interferencia más que mínima con sus actividades sociales y funcionales habituales	Dolor muscular que impide realizar sus actividades sociales y funcionales habituales	Dolor muscular incapacitante que impide realizar las actividades diarias básicas
OSTEONECROSIS	NA	Asintomática pero con hallazgo por radiografía Y sin indicación de intervención quirúrgica	Dolor óseo con hallazgo por radiografía O indicación de intervención quirúrgica	Dolor óseo incapacitante con hallazgo por radiografía que impide realizar las actividades diarias básicas
Osteopenia[6] *≥ 30 años*	DMO calificación T entre 2.5 y -1	NA	NA	NA
< 30 años	DMO calificación Z	NA	NA	NA

	entre -2 y -1			
Osteoporosis[6]<br>*≥ 30 años*	NA	DMO calificación T < -2.5	Fractura patológica (ej. fractura por compresión que provoca pérdida de altura vertebral)	Fractura patológica con consecuencias de riesgo de vida
< 30 years of age	NA	DMO calificación Z < -2	Fractura patológica (ej. fractura por compresión que provoca pérdida de altura vertebral)	Fractura patológica con consecuencias de riesgo de vida

6- Las calificaciones T y Z de DMO se encuentran en: Kanis JA on behalf of the World Health Organization Scientific Group (2007). Assessment of osteoporosis at the primary health-care level. Technical Report. World Health Organization Collaborating Centre for Metabolic Bone Diseases, University of Sheffield, UK. 2007: Printed by the University of Sheffield

Neurológico

PARÁMETRO	**TOXICIDAD GRADO 1 LEVE**	**TOXICIDAD GRADO 2 MODERADO**	**TOXICIDAD GRADO 3 SEVERA**	**TOXICIDAD GRADO 4 CON RIESGO DE VIDA**
Isquemia del SNC aguda	NA	NA	Ataque isquémico transitorio	Accidente cerebro vascular (ej. derrame cerebral con déficit neurológico)
ESTADO MENTAL ALTERADO (Demencia se encuentra en *Trastorno cognitivo, de conducta o de atención* más abajo)	Cambios que provocan ninguna o una mínima interferencia con sus actividades sociales y funcionales habituales	Leve letargo o somnolencia que provoca una interferencia más que mínima con sus actividades sociales y funcionales habituales	Confusión, deterioro de la memoria, letargo o somnolencia que impiden realizar las actividades diarias básicas	Delirium U Obnubilación O Coma
ATAXIA	Cambios que provocan ninguna o una mínima interferencia con sus actividades sociales y funcionales habituales O carencia de síntomas, la ataxia se detecta durante la consulta médica	Síntomas que provocan una interferencia más que mínima con sus actividades sociales y funcionales habituales	Síntomas que impiden realizar sus actividades sociales y funcionales habituales a actividad social y funcional	Síntomas incapacitantes que impiden realizar las actividades diarias básicas
TRASTORNO COGNITIVO, DE CONDUCTA O DE ATENCIÓN (incluye demencia trastorno por déficit de atención) *Especificar el tipo de trastorno, si aplica*	Discapacidad que provoca ninguna o una mínima interferencia con sus actividades sociales y funcionales habituales O sin indicación de recursos especiales	Discapacidad que provoca una interferencia más que mínima con sus actividades sociales y funcionales habituales O indicación de recursos especiales a tiempo parcial	Discapacidad que impide realizar sus actividades sociales y funcionales habituales O indicación de recursos especiales a tiempo completo	Discapacidad que impide realizar las actividades diarias básicas O indicación de institucionalización
RETRASO EN EL DESARROLLO	Retraso leve, ya sea de tipo motriz o	Retraso moderado, ya sea de tipo	Retraso severo, ya sea de tipo motriz o	Regresión en el desarrollo, ya sea

< 18 años<br>*Especificar el tipo de retraso, si aplica*	cognitivo, utilizando herramientas de diagnóstico apropiadas	motriz o cognitivo, utilizando herramientas de diagnóstico apropiadas	cognitivo, utilizando herramientas de diagnóstico apropiadas	de tipo motriz o cognitivo, utilizando herramientas de diagnóstico apropiadas
DOLOR DE CABEZA	Síntomas que provocan ninguna o una mínima interferencia con sus actividades sociales y funcionales habituales	Síntomas que provocan una interferencia más que mínima con sus actividades sociales y funcionales habituales	Síntomas que impiden realizar sus actividades sociales y funcionales habituales	Síntomas que impiden realizar las actividades diarias básicas O indicación de hospitalización O dolor de cabeza que afecta significativamente el nivel de alerta u otra función neurológica
DEBILIDAD NEUROMUSCULAR (incluye miopatía y neuropatía)<br>*Especificar tipo, si aplica*	Debilidad muscular mínima que provoca ninguna o una mínima interferencia con sus actividades sociales y funcionales habituales O ningún síntoma con falta de fuerza detectada durante la consulta médica	Debilidad muscular que provoca una interferencia más que mínima con sus actividades sociales y funcionales habituales la actividad social y funcional	Debilidad muscular que impide realizar sus actividades sociales y funcionales habituales	Debilidad muscular incapacitante que impide realizar las actividades diarias básicas O debilidad de los músculos respiratorios que afecta la ventilación
ALTERACIONES NEUROSENSORIALES (incluye parestesia neuropatía dolorosa)<br>*Especificar tipo, si aplica*	Parestesia mínima que provoca ninguna o una mínima interferencia con sus actividades sociales y funcionales habituales O ningún síntoma con alteración sensorial detectada durante la consulta médica	Alteración sensorial o parestesia que provoca una interferencia más que mínima con sus actividades sociales y funcionales habituales	Alteración sensorial o parestesia que impide realizar sus actividades sociales y funcionales habituales	Alteración sensorial o parestesia incapacitante que impide realizar las actividades diarias básicas
CONVULSIONES<br>*Convulsiones de primera vez*<br>*≥ 18 años*	NA	NA	De 1 a 3 convulsiones	Convulsiones prolongadas y reiteradas (ej. status epilepticus) O difíciles de controlar (ej. epilepsia refractaria)
< 18 años<br>*(incluye convulsiones febriles nuevas o preexistentes)*	Convulsiones que duren < 5minutos con < 24 horas de estado postictal	Convulsiones que duren entre 5 y < 20 minutos con < 24 horas de estado postictal	Convulsiones que duren ≥ 20 minutos O > 24 horas de estado postictal	Convulsiones prolongadas y reiteradas (ej. status epilepticus) O difíciles de controlar (ej. epilepsia refractaria)
Convulsiones preexistentes	NA	Mayor frecuencia respecto al anterior nivel de control sin cambios en el tipo	Cambio en el tipo de convulsiones, sea en su duración o en su naturaleza	Convulsiones prolongadas y reiteradas (ej. status epilepticus)

		de convulsiones	(ej. severidad o focalidad)	O difíciles de controlar (ej. epilepsia refractaria)
SÍNCOPE	Casi síncope sin pérdida del conocimiento (ej. presíncope)	Pérdida del conocimiento sin indicación de intervención	Pérdida de conocimiento Y que requiere hospitalización o intervención	NA

Embarazo, puerperio y perinatal

PARÁMETRO	**TOXICIDAD GRADO 1 LEVE**	**TOXICIDAD GRADO 2 MODERADO**	**TOXICIDAD GRADO 3 SEVERA**	**TOXICIDAD GRADO 4 CON RIESGO DE VIDA**
MUERTE FETAL (reportar usando la identificación de la madre participante)	NA	NA	Pérdida del feto a ≥ 20 semanas de gestación	NA
Parto prematuro[7] (reportar usando la identificación de la madre participante)	Parto entre la semana 34 a < 37 de gestación	Parto entre la semana 28 a < 34 de gestación	Parto entre la semana 24 a < 28 de gestación	Parto a < 24 semanas de gestación
ABORTO ESPONTÁNEO O ABORTO INVOLUNTARIO[8] (reportar usando la identificación de la madre participante) *Reportar solo uno*	Embarazo químico	Aborto espontáneo o involuntario sin complicaciones	Aborto espontáneo o involuntario con complicaciones	NA

7- Definición: Parto de un neonato vivo entre las semanas ≥ 20 a < 37 de gestación.
8- Definición: Diagnóstico clínico de embarazo que ocurre en < 20 semanas de gestación.

Psiquiátricos

PARÁMETRO	**TOXICIDAD GRADO 1 LEVE**	**TOXICIDAD GRADO 2 MODERADO**	**TOXICIDAD GRADO 3 SEVERA**	**TOXICIDAD GRADO 4 CON RIESGO DE VIDA**
INSOMNIO	Dificultad leve para conciliar el sueño o para permanecer dormido; o insomnio tardío	Dificultad moderada para conciliar el sueño o para permanecer dormido; o insomnio tardío	Dificultad severa para conciliar el sueño o para permanecer dormido; o insomnio tardío	NA
TRASTORNOS PSIQUIÁTRICOS (incluye ansiedad, depresión, manía, y psicosis) *Especificar el trastorno*	Síntomas sin indicación de intervención O comportamiento que provoca ninguna o una mínima interferencia con sus actividades sociales y	Síntomas con indicación de intervención O comportamiento que provoca una más que mínima interferencia con sus actividades sociales y funcionales	Síntomas con indicación de hospitalización O comportamiento que impide realizar sus actividades sociales y funcionales habituales	Amenaza de daño a sí mismo o a otros O psicosis aguda O comportamiento que impide realizar las actividades diarias básicas

	funcionales habituales a actividad social y funcional habitual	habituales		
IDEACIÓN SUICIDA O INTENTO DE SUICIDIO *Reportar solo uno*	Pensamiento recurrente en la muerte Y sin deseo de cometer suicidio	Pensamiento recurrente en la muerte Y con deseo de cometer suicidio sin un plan específico	Idea de suicidarse con planes parciales o completos de cómo hacerlo pero sin haberlo intentado O indicación de hospitalización	Intento de suicidio

Respiratorio

PARÁMETRO	**TOXICIDAD GRADO 1 LEVE**	**TOXICIDAD GRADO 2 MODERADO**	**TOXICIDAD GRADO 3 SEVERA**	**TOXICIDAD GRADO 4 CON RIESGO DE VIDA**
BRONCOESPASMO AGUDO	Volumen espiratorio forzado en 1 segundo o flujo máximo reducido de ≥ 70 a < 80% O síntomas leves sin indicación de intervención	Volumen espiratorio forzado en 1 segundo o flujo máximo de 50 a < 70% O síntomas con indicación de intervención O síntomas que provocan una interferencia más que mínima con sus actividades sociales y funcionales habituales	Volumen espiratorio forzado en 1 segundo o flujo máximo de 25 a < 50% O síntomas que impiden realizar sus actividades sociales y funcionales habituales	Volumen espiratorio forzado en 1 segundo o flujo máximo de < 25% O compromiso respiratorio o hemodinámico O intubación
DISNEA O DIFICULTAD RESPIRATORIA *Reportar solo una*	Disnea al hacer esfuerzo con ninguna o una mínima interferencia con las actividades sociales y funcionales habituales O sibilancia O aumento mínimo en la frecuencia respiratoria para su edad	Disnea al hacer esfuerzo con una más que mínima interferencia con las actividades sociales y funcionales habituales O aleteo nasal O retracciones intercostales U oximetría de pulso de 90 a < 95%	Disnea en reposo que impide realizar sus actividades sociales y funcionales habituales U oximetría de pulso de < 90%	Insuficiencia respiratoria con indicación de asistencia respiratoria mecánica (ej. CPAP, BiPAP, intubación)

Sentidos

PARÁMETRO	**TOXICIDAD GRADO 1 LEVE**	**TOXICIDAD GRADO 2 MODERADO**	**TOXICIDAD GRADO 3 SEVERA**	**TOXICIDAD GRADO 4 CON RIESGO DE VIDA**
PÉRDIDA DE LA AUDICIÓN *< 12 años (basado en un audiograma de 1,*	NA	Sin indicación de audífono ni intervención	Con indicación de audífono o intervención	Hipoacusia bilateral severa (> 80 dB a 2 kHz o más) O pérdida de la audición útil (ej.

2, 3, 4, 6 y 8 kHz)				audiograma de > 50 dB y < 50% de discriminación del habla)
≥ 12 años	Pérdida auditiva de > 20 dB a ≤ 4 kHz	Pérdida auditiva de > 20 dB a > 4 kHz	Pérdida auditiva de > 20 dB a ≥ 3 kHz en un oído con indicación de servicios adicionales relacionados al habla y lenguaje (donde estén disponibles) O pérdida auditiva suficiente para indicar intervención terapéutica y/o audífonos	Indicación audiológica de implante coclear y servicios adicionales relacionados al habla y lenguaje (donde estén disponibles)
TINNITUS	Síntomas que provocan ninguna o una mínima interferencia con sus actividades sociales y funcionales habituales sin indicación de intervención	Síntomas que provocan una más que mínima interferencia con sus actividades sociales y funcionales habituales con indicación de intervención	Síntomas que impiden realizar sus actividades sociales y funcionales habituales	NA
UVEÍTIS	Asintomática Y detectable por examen	Uveítis anterior con síntomas O indicación de intervención (Medicamylasal)	Uveítis posterior o panuveítis O indicación de intervención quirúrgica	Pérdida de visión incapacitante
VÉRTIGO	Vértigo que provoca ninguna o una mínima interferencia con sus actividades sociales y funcionales habituales	Vértigo que provoca una más que mínima interferencia con sus actividades sociales y funcionales habituales	Vértigo que impide realizar sus actividades sociales y funcionales habituales	Vértigo incapacitante que impide realizar las actividades diarias básicas
CAMBIOS EN LA VISIÓN (desde la visita basal)	Cambios en la visión que provocan ninguna o una mínima interferencia con sus actividades sociales y funcionales habituales	Cambios en la visión que provocan una más que mínima interferencia con sus actividades sociales y funcionales habituales	Cambios en la visión que impiden realizar sus actividades sociales y funcionales habituales	Pérdida de visión incapacitante

Sistémico

PARÁMETRO	**TOXICIDAD GRADO 1 LEVE**	**TOXICIDAD GRADO 2 MODERADO**	**TOXICIDAD GRADO 3 SEVERA**	**TOXICIDAD GRADO 4 CON RIESGO DE VIDA**
REACCIÓN ALÉRGICA AGUDA	Urticaria localizada (ronchas) sin indicación de intervención	Urticaria localizada con indicación de intervención O angioedema leve	Urticaria generalizada O angioedema con indicación de	Anafilaxia aguda O broncoespasmo con riesgo de vida O edema laríngeo

	médica	sin indicación de intervención	intervención O síntomas de broncoespasmo leve	
ESCALOFRÍOS	Síntomas que provocan ninguna o una mínima interferencia con sus actividades sociales y funcionales habituales	Síntomas que provocan una más que mínima interferencia con sus actividades sociales y funcionales habituales	Síntomas que impiden realizar sus actividades sociales y funcionales habituales	NA
SÍNDROME DE LIBERACIÓN DE CITOQUINAS[9]	Signos y síntomas leves Y sin indicación de interrumpir el tratamiento (ej. infusión de anticuerpos)	Indicación de interrumpir el tratamiento (ej. infusión de anticuerpos) Y responde de inmediato al tratamiento de los síntomas O indicación de tratamiento profiláctico < 24 horas	Signos y síntomas severos y prolongados O reaparición de síntomas luego de una mejora inicial	Consecuencias con riesgo de vida (ej. que requiere vasopresores o asistencia respiratoria mecánica
FATIGA O MALESTAR *Reportar solo uno*	Síntomas que provocan ninguna o una mínima interferencia con sus actividades sociales y funcionales habituales	Síntomas que provocan una más que mínima interferencia con sus actividades sociales y funcionales habituales	Síntomas que impiden realizar sus actividades sociales y funcionales habituales	Síntomas incapacitantes de fatiga o malestar que impide realizar las actividades diarias básicas
FIEBRE (solo temperatura no axilar)	38.0 a < 38.6°C o 100.4 a < 101.5°F	≥ 38.6 a < 39.3°C o ≥ 101.5 a < 102.7°F	≥ 39.3 a < 40.0°C o ≥ 102.7 a < 104.0°F	≥ 40.0°C o ≥ 104.0°F
DOLOR[10] (no asociado a la droga de estudio y no especificado en otra sección) *Especificar ubicación*	Dolor que provoca ninguna o una mínima interferencia con sus actividades sociales y funcionales habituales	Dolor que provoca una más que mínima interferencia con sus actividades sociales y funcionales habituales	Dolor que impide realizar sus actividades sociales y funcionales habituales	Dolor incapacitante que impide realizar las actividades diarias básicas O indicación de hospitalización
ENFERMEDAD DEL SUERO[11]	Signos y síntomas leves	Signos y síntomas leves E indicación de intervención (ej. antihistamínicos)	Signos y síntomas severos E indicación de intervención mayor (ej. esteroides o fluidos intravenosos)	Consecuencias con riesgo de vida (ej. que requiere vasopresores o asistencia respiratoria mecánica
BAJO PESO[12] *> 5 a 19 años*	NA	IMC de la OMS calificación Z < -2 a ≤ -3	IMC de la OMS calificación Z < -3	IMC de la OMS calificación Z < -3 con consecuencias de riesgo de vida
De 2 a 5 años	NA	OMS peso para la estatura calificación Z < -2 a ≤ -3	OMS peso para la estatura calificación Z < -3	OMS peso para la estatura calificación Z < -3 con consecuencias de riesgo de vida
< 2 años	NA	OMS peso para la longitud calificación	OMS peso para la longitud calificación	OMS peso para la longitud calificación

		Z < -2 a ≤ -3	Z < -3	Z < -3 con consecuencias de riesgo de vida
PÉRDIDA DE PESO (no incluye la pérdida de peso postparto)	NA	Pérdida de 5 a < 9% del peso basal	Pérdida de ≥ 9 a < 20% del peso basal	Pérdida de ≥ 20% del peso basal O indicación de intervención agresiva (ej. alimentación por sonda, nutrición parenteral)

9- Definición: Trastorno que consiste en náusea, dolor de cabeza, taquicardia, hipotensión, rash, y/o falta de aire.
10 Ver dolor asociado a inyecciones o infusiones en la sección *Reacciones locales en el punto de inyección o infusión* (pág. 23).
11 Definición: Trastorno que consiste en fiebre, artralgia, mialgia, erupciones cutáneas, linfoadenopatías, malestar marcado, y/o disnea.
12 Se puede accede a las tablas de la OMS hacienda clic sobre el rango etario deseado o en las siguientes direcciones URL: http://www.who.int/growthref/who2007_bmi_for_age/en/ para los participantes de > 5 a 19 años de edad y http://www.who.int/childgrowth/standards/chart_catalogue/en/ para los de ≤ 5 años.

Urinario

PARÁMETRO	**TOXICIDAD GRADO 1 LEVE**	**TOXICIDAD GRADO 2 MODERADO**	**TOXICIDAD GRADO 3 SEVERA**	**TOXICIDAD GRADO 4 CON RIESGO DE VIDA**
OBSTRUCCIÓN DEL TRACTO URINARIO	NA	Signos o síntomas de obstrucción del tracto urinario sin hidronefrosis ni insuficiencia renal	Signos o síntomas de obstrucción del tracto urinario con hidronefrosis o insuficiencia renal	Obstrucción con consecuencias de riesgo de vida

Reacciones locales en el punto de inyección o infusión

PARÁMETRO	**TOXICIDAD GRADO 1 LEVE**	**TOXICIDAD GRADO 2 MODERADO**	**TOXICIDAD GRADO 3 SEVERA**	**TOXICIDAD GRADO 4 CON RIESGO DE VIDA**
DOLOR O SENSIBILIDAD EN EL PUNTO DE INYECCIÓN *Reportar solo uno*	Dolor o sensibilidad que provoca ninguna o una mínima limitación de movimiento en la extremidad	Dolor o sensibilidad que provoca una más que mínima limitación de movimiento en la extremidad	Dolor o sensibilidad que impide realizar las actividades sociales y funcionales habituales	Dolor sensibilidad que impide realizar las actividades diarias básicas O indicación de hospitalización
ERITEMA O ENROJECIMIENTO EN EL PUNTO DE INYECCIÓN[13] *Reportar solo uno* *> 15 años*	De 2.5 a < 5 cm de diámetro O área de superficie de 6.25 a < 25 cm^2 Y síntomas que provocan ninguna o una mínima interferencia con las actividades sociales y funcionales habituales	De ≥ 5 a < 10 cm de diámetro O ≥ área de superficie de 25 a < 100 cm^2 O síntomas que provocan una más que mínima interferencia con las actividades sociales y funcionales habituales	De ≥ 10 cm de diámetro O área de superficie de ≥ 100 cm^2 O ulceración O infección secundaria O flebitis O absceso estéril O drenaje O síntomas que impiden realizar las actividades sociales y funcionales habituales	Consecuencias potencialmente con riesgo de vida (ej. absceso, dermatitis exfoliativa, necrosis cutánea o subcutánea)
≤ 15 años	≤ 2.5 cm de diámetro	> 2.5 cm de diámetro con un área de superficie < 50% del segmento	Área de superficie ≥ 50% del segmento de la extremidad (ej. parte superior	Consecuencias potencialmente con riesgo de vida (ej. absceso, dermatitis

		de la extremidad (ej. parte superior del brazo o muslo)	del brazo o muslo) O ulceración O infección secundaria O flebitis O absceso estéril O drenaje	exfoliativa, necrosis cutánea o subcutánea)
INDURACIÓN O HINCHAZÓN EN EL PUNTO DE INYECCIÓN *Reportar solo uno* *> 15 años*	Igual que ERITEMA O ENROJECIMIENTO EN EL PUNTO DE INYECCIÓN *> 15 años*	Igual que ERITEMA O ENROJECIMIENTO EN EL PUNTO DE INYECCIÓN *> 15 años*	Igual que ERITEMA O ENROJECIMIENTO EN EL PUNTO DE INYECCIÓN *> 15 años*	Igual que ERITEMA O ENROJECIMIENTO EN EL PUNTO DE INYECCIÓN *> 15 años*
≤ 15 años	Igual que ERITEMA O ENROJECIMIENTO EN EL PUNTO DE INYECCIÓN, *≤ 15 años*	Igual que ERITEMA O ENROJECIMIENTO EN EL PUNTO DE INYECCIÓN, *≤ 15 años*	Igual que ERITEMA O ENROJECIMIENTO EN EL PUNTO DE INYECCIÓN, *≤ 15 años*	Igual que ERITEMA O ENROJECIMIENTO EN EL PUNTO DE INYECCIÓN, *≤ 15 años*
PRURITO EN EL PUNTO DE INYECCIÓN	Prurito localizado en el punto de inyección que se resuelve espontáneamente o con < 48 horas de tratamiento	Prurito que excede el punto de inyección pero no generalizado O prurito localizado en el punto de inyección que requiere ≥ 48 horas de tratamiento	Prurito generalizado que impide realizar las actividades sociales y funcionales habituales	NA

13- Los eritemas o enrojecimientos en el punto de inyección deben evaluarse y calificarse utilizando el diámetro o el área de superficie más grande.

Valores de laboratorio

Química

PARÁMETRO	**TOXICIDAD GRADO 1 LEVE**	**TOXICIDAD GRADO 2 MODERADO**	**TOXICIDAD GRADO 3 SEVERA**	**TOXICIDAD GRADO 4 CON RIESGO DE VIDA**
ACIDOSIS	NA	pH ≥ 7.3 a < límite inferior normal (LIN)	pH < 7.3 sin consecuencias con riesgo de vida	pH < 7.3 con consecuencias con riesgo de vida
ALBÚMINA SÉRICA BAJA (g/dL; *g/L*)	3.0 a < LIN *30 a* < LIN	≥ 2.0 a < 3.0 *≥ 20 a < 30*	< 2.0 *< 20*	NA
FOSFATASA ALCALINA ELEVADA	1.25 a < 2.5 x Límite superior Normal (LSN)	2.5 a < 5.0 x LSN	5.0 a < 10.0 x LSN	≥ 10.0 x LSN
ALCALOSIS	NA	pH > LSN a ≤ 7.5	pH > 7.5 sin consecuencias con riesgo de vida	pH > 7.5 con consecuencias con riesgo de vida
ELEVACIÓN DE ALT o SGPT *(reportar solo una)*	1.25 a < 2.5 x LSN	2.5 a < 5.0 x LSN	5.0 a < 10.0 x LSN	≥ 10.0 x LSN
AMILASA (Pancreática) o AMILASA (Total), *(reportar solo una)*	1.1 a < 1.5 x LSN	1.5 a < 3.0 x LSN	3.0 a < 5.0 x LSN	≥ 5.0 x LSN
ELEVACIÓN DE AST o SGOT, *(reportar solo una)*	1.25 a < 2.5 x LSN	2.5 a < 5.0 x LSN	5.0 a < 10.0 x LSN	≥ 10.0 x LSN
DISMINUCIÓN DEL BICARBONATO (mEq/L;	16.0 a < LIN *16.0 a* < LIN	11.0 a < 16.0 *11.0 a < 16.0*	8.0 a < 11.0 *8.0 a < 11.0*	< 8.0 *< 8.0*

mmol/L)				
BILIRRUBINA HIPERBILIRRUBINEMIA DIRECTA [14]<br>*> 28 días de edad*	NA	NA	> LSN	> LSN con consecuencias con riesgo de vida (ej. signos y síntomas de falla hepática)
≤ 28 días de edad	LSN a ≤ 1 mg/dL	> 1 a ≤ 1.5 mg/dL	> 1.5 a ≤ 2 mg/dL	> 2 mg/dL
HIPERBILIRRUBINEMIA TOTAL<br>*> 28 días de edad*	1.1 a < 1.6 x LSN	1.6 a < 2.6 x LSN	2.6 a < 5.0 x LSN	≥ 5.0 x LSN
≤ 28 días de edad	Ver Apéndice A : Bilirrubina Total para neonatos de término y pretérmino	Ver Apéndice A : Bilirrubina Total para neonatos de término y pretérmino	Ver Apéndice A : Bilirrubina Total para neonatos de término y pretérmino	Ver Apéndice A : Bilirrubina Total para neonatos de término y pretérmino
HIPERCALCEMIA (mg/dL; *mmol/L*)<br>*≥ 7 días de edad*	10.6 a < 11.5<br>*2.65 a < 2.88*	11.5 a < 12.5<br>*2.88 a < 3.13*	12.5 a < 13.5<br>*3.13* to *< 3.38*	≥ 13.5 ≥ *3.38*
< 7 días de edad	11.5 a < 12.4<br>*2.88* a *< 3.10*	12.4 a < 12.9<br>*3.10* a *< 3.23*	12.9 a < 13.5 *3.23* a *< 3.38*	≥ 13.5 ≥ *3.38*
ELEVACIÓN DEL CALCIO IÓNICO<br>(mg/dL; *mmol/L*)	> LSN a < 6.0<br>*> LSN a < 1.5*	6.0 a < 6.4<br>*1.5 a < 1.6*	6.4 a < 7.2<br>*1.6 a < 1.8*	≥ 7.2 ≥ *1.8*
HIPOCALCEMIA<br>(mg/dL; *mmol/L*)<br>*≥ 7 días de edad*	7.8 a < 8.4<br>*1.95* a *< 2.10*	7.0 a < 7.8<br>*1.75* a *< 1.95*	6.1 a < 7.0<br>*1.53* a *< 1.75*	< 6.1 < *1.53*
< 7 días de edad	6.5 a < 7.5<br>*1.63* a *< 1.88*	6.0 a < 6.5<br>*1.50* a *< 1.63*	5.50 a < 6.0<br>*1.38* a *< 1.50*	< 5.50 < *1.38*
DISMINUCIÓN DEL CALCIO IÓNICO<br>(mg/dL; *mmol/L*)	< LIN a 4.0<br>*< LIN a 1.0*	3.6 a < 4.0<br>*0.9 a < 1.0*	3.2 a < 3.6<br>*0.8 a < 0.9*	< 3.2 < *0.8*
ELEVACIÓN DE TROPONINA CARDÍACA	NA	NA	NA	Niveles consistentes con angina inestable o IAM según laboratorio local
ELEVACIÓN DE CPK	3 a < 6 x LSN	6 a < 10 x LSN	10 a < 20 x LSN	≥ 20 x LSN
ELEVACIÓN DE CREATININA	1.1 a 1.3 x LSN	> 1.3 a 1.8 x LSN O aumento > 0.3 mg/dL del basal	> 1.8 a < 3.5 x LSN O aumento de 1.5 a < 2.0 x encima del valor basal	≥ 3.5 x LSN O aumento de ≥ 2.0 x encima del valor basal
DISMINUCIÓN DE CREATININA O DEL eGFR [15]<br>*(reportar solo una)*	NA	< 90 a 60 ml/min o ml/min/1.73 m2 O 10 a < 30% de descenso con respecto al basal	< 60 a 30 ml/min o ml/min/1.73 m2 O ≥ 30 a < 50% de descenso con respecto al basal	< 30 ml/min o ml/min/1.73 m2 O ≥ 50% de descenso con respecto al basal o necesidad de diálisis
HIPERGLUCEMIA EN AYUNAS<br>(mg/dL; *mmol/L*)	110 a 125<br>*6.11 a < 6.95*	> 125 a 250<br>*6.95 a < 13.89*	> 250 a 500<br>*13.89 a < 27.75*	> 500<br>*≥ 27.75*
HIPERGLUCEMIA SIN AYUNO	116 a 160<br>*6.44 a < 8.89*	> 160 a 250<br>*8.89 a < 13.89*	> 250 a 500<br>*13.89 a < 27.75*	> 500<br>*≥ 27.75*
HIPOGLUCEMIA<br>(mg/dL; *mmol/L*)<br>*≥ 1 mes de edad*	55 a 64<br>*3.05 a 3.55*	40 a < 55<br>*2.22 a < 3.05*	30 a < 40<br>*1.67 a < 2.22*	< 30<br>*< 1.67*
< 1 mes de edad	50 a 54	40 a < 50	30 a < 40	< 30

	2.78 a 3.00	*2.22 a < 2.78*	*1.67 a < 2.22*	*< 1.67*
ELEVACIÓN DE LACTATO	LSN a < 2.0 x LSN sin acidosis	≥ 2.0 x LSN sin acidosis	Elevación de lactato con pH < 7.3 sin consecuencias con riesgo de vida	Elevación de lactato con pH < 7.3 con consecuencias con riesgo de vida
ELEVACIÓN DE LIPASA	1.1 a < 1.5 x LSN	1.5 a < 3.0 x LSN	3.0 a < 5.0 x LSN	≥ 5.0 x LSN
DISLIPIDEMIA (mg/dL; *mmol/L*) *HIPERCOLESTEROLEMIA EN AYUNAS ≥ 18 años de edad*	200 a < 240 *5.18 a < 6.19*	240 a < 300 *6.19 a < 7.77*	≥ 300 ≥ *7.77*	NA
< 18 años de edad	170 a < 200 *4.40 a < 5.15*	200 a < 300 *5.15 a < 7.77*	≥ 300 *≥ 7.77*	NA
ELEVACIÓN DE LDL, EN AYUNAS ≥ 18 años de edad	130 a < 160 *3.37 a < 4.12*	160 a < 190 *4.12 a < 4.90*	≥ 190 *≥ 4.90*	NA
> 2 a < 18 años de edad	110 a < 130 *2.85 a < 3.34*	130 a < 190 *3.34 a < 4.90*	≥ 190 *≥ 4.90*	NA
HIPERTRIGLICERIDEMIA EN AYUNAS	150 a 300 *1.71 a 3.42*	>300 a 500 *>3.42 a 5.7*	>500 a < 1,000 *>5.7 a 11.4*	> 1,000 *> 11.4*
HIPOMAGNESEMIA[16] (mEq/L; *mmol/L*)	1.2 a < 1.4 *0.60 a < 0.70*	0.9 a < 1.2 *0.45 a < 0.60*	0.6 a < 0.9 *0.30 a < 0.45*	< 0.6 *< 0.30*
HIPOFOSFATEMIA (mg/dL; *mmol/L*) *> 14 años de edad*	2.0 a < LIN *0.81 a < LIN*	1.4 a < 2.0 *0.65 a < 0.81*	1.0 a < 1.4 *0.32 a < 0.65*	< 1.0 *< 0.32*
1 a 14 años de edad	3.0 a < 3.5 *0.97 a < 1.13*	2.5 a < 3.0 *0.81 a < 0.97*	1.5 a < 2.5 *0.48 a < 0.81*	< 1.5 *< 0.48*
< 1 año de edad	3.5 a < 4.5 *1.13 a < 1.45*	2.5 a < 3.5 *0.81 a < 1.13*	1.5 a < 2.5 *0.48 a < 0.81*	< 1.5 *< 0.48*
HIPERKALEMIA (mEq/L; *mmol/L*)	5.6 a < 6.0 *5.6 a < 6.0*	6.0 a < 6.5 *6.0 a < 6.5*	6.5 a < 7.0 *6.5 a < 7.0*	≥ 7.0 *≥ 7.0*
HIPOKALEMIA (mEq/L; *mmol/L*)	3.0 a < 3.4 *3.0 a < 3.4*	2.5 a < 3.0 *2.5 a < 3.0*	2.0 a < 2.5 *2.0 a < 2.5*	< 2.0 *< 2.0*
HIPERNATREMIA (mEq/L; *mmol/L*)	146 a < 150 *146 a < 150*	150 a < 154 *150 a < 154*	154 a < 160 *154 a < 160*	≥ 160 *≥ 160*
HIPONATREMIA (mEq/L; *mmol/L*)	130 a < 135 *130 a < 135*	125 a < 130 *125 a < 135*	121 a < 125 *121 a < 125*	≤ 120 *≤ 120*
HIPERURICEMIA (mg/dL; *mmol/L*)	7.5 a < 10.0 *0.45 a < 0.59*	10.0 a < 12.0 *0.59 a < 0.71*	12.0 a < 15.0 *0.71 a < 0.89*	≥ 15.0 *≥ 0.89*

14 Bilirrubina directa > 1.5 mg/dL en un participante < 28 días de edad debe graduarse como grado 2 si es < 10% de la bilirrubina total.
15 Usar fórmula: (ej. : Cockroft-Gault in mL/min o Schwartz in mL/min/1.73m2).
16 Para convertir el magnesio en mg/dL a mmol/L, se debe multiplicar por 0.4114.

Hematología

PARÁMETRO	AXICIDAD GRADO 1 LEVE	AXICIDAD GRADO 2 MODERADO	AXICIDAD GRADO 3 SEVERA	AXICIDAD GRADO 4 CON RIESGO DE VIDA
DISMINUCIÓN DEL RECUENTO DE CD4+ (cel./mm3; *cel./L*) *> 5años de edad (no infectado con HIV)*	300 a < 400 *300 a < 400*	200 a < 300 *200 a < 300*	100 a < 200 *100 a < 200*	< 100 *< 100*
LINFOPENIAS (cell/mm3; *cells/L*) (cel./mm3; *cel./L*)	600 a < 650 *0.600 x 109 a < 0.650 x 109*	500 a < 600 *0.500 x 109 a < 0.600 x 109*	350 a < 500 *0.350 x 109 a < 0.500 x 109*	< 350 *< 0.350 x 109*

> 5años de edad (no infectado con HIV)				
NEUTROPENIA<br>(cells/mm3; *cells/L*)<br>*> 7 días de edad*	800 a 1,000<br>*0.800 x 109 a 1.000 x 109*	600 a 799<br>*0.600 x 109 a 0.799 x 109*	400 a 599<br>*0.400 x 109 a 0.599 x 109*	< 400<br>*< 0.400 x 109*
2 a 7 días de edad	1,250 a 1,500<br>*1.250 x 109 a 1.500 x 109*	1,000 a 1,249<br>*1.000 x 109 a 1.249 x 109*	750 a 999<br>*0.750 x 109 a 0.999 x 109*	< 750<br>*< 0.750 x 109*
≤ 1 días de edad	4,000 a 5,000<br>*4.000 x 109 a 5.000 x 109*	3,000 a 3,999<br>*3.000 x 109 a 3.999 x 109*	1,500 a 2,999<br>*1.500 x 109 a 2.999 x 109*	< 1,500<br>*< 1.500 x 109*
DISMINUCIÓN DE FIBRINÓGENO (mg/dL; *g/L*)	100 a < 200<br>*1.00 a < 2.00* O 0.75 a < 1.00 x LIN	75 a < 100<br>*0.75 a < 1.00* O ≥ 0.50 a < 0.75 x LIN	50 a < 75<br>*0.50 a < 0.75* O 0.25 a < 0.50 x LIN	< 50 *< 0.50*<br>O < 0.25 x LIN O asociado a sangrado importante
DISMINUCIÓN DE HEMOGLOBINA 17, (g/dL; *mmol/L*)18<br>*≥ 13 años de edad sexo masculino)*	10.0 a 10.9<br>*6.19 a 6.76*	9.0 a < 10.0<br>*5.57 a < 6.19*	7.0 a < 9.0<br>*4.34 a < 5.57*	< 7.0<br>*< 4.34*
≥ 13 años de edad sexo femenino)	9.5 a 10.4<br>*5.88 a 6.48*	8.5 a < 9.5<br>*5.25 a < 5.88*	6.5 a < 8.5<br>*4.03 a < 5.25*	< 6.5<br>*< 4.03*
57 días de edad a < 13años de edad ambos sexos)	9.5 a 10.4<br>*5.88 a 6.48*	8.5 a < 9.5<br>*5.25 a < 5.88*	6.5 a < 8.5<br>*4.03 a < 5.25*	< 6.5<br>*< 4.03*
36 a 56 días de edad (ambos sexos)	8.5 a 9.6<br>*5.26 a 5.99*	7.0 a < 8.5<br>*4.32 a < 5.26*	6.0 a < 7.0<br>*3.72 a < 4.32*	< 6.0<br>*< 3.72*
22 a 35 días de edad (ambos sexos)	9.5 a 11.0<br>*5.88 a 6.86*	8.0 a < 9.5<br>*4.94 a < 5.88*	6.7 a < 8.0<br>*4.15 a < 4.94*	< 6.7<br>*< 4.15*
8 a ≤ 21 días de edad (ambos sexos)	11.0 a 13.0<br>*6.81 a 8.10*	9.0 a < 11.0<br>*5.57 a < 6.81*	8.0 a < 9.0<br>*4.96 a < 5.57*	< 8.0<br>*< 4.96*
≤ 7 días de edad (ambos sexos))	13.0 a 14.0<br>*8.05 a 8.72*	10.0 a < 13.0<br>*6.19 a < 8.05*	9.0 a < 10.0<br>*5.59 a < 6.19*	< 9.0<br>*< 5.59*
ELEVACIÓN DEL RIN (en pacientes que NO reciben anticoagulantes)	1.1 a < 1.5 x LSN	1.5 a < 2.0 x LSN	2.0 a < 3.0 x LSN	≥ 3.0 x LSN
METAHEMOGLOBINEMIA (% hemoglobina)	5.0 a < 10.0%	10.0 a < 15.0%	15.0 a < 20.0%	≥ 20.0%
AUMENTO DE PTT,<br>(en pacientes que NO reciben anticoagulantes)	1.1 a < 1.66 x LSN	1.66 a < 2.33 x LSN	2.33 a < 3.00 x LSN	≥ 3.00 x LSN
PLAQUETOPENIAS,<br>(cel./mm3; *cel./L*)	100,000 a < 124,999 *100.000 x 109 a < 124.999 x 109*	50,000 a < 100,000 *50.000 x 109 a < 100.000 x 109*	25,000 a < 50,000<br>*25.000 x 109 a < 50.000 x 109*	< 25,000 *< 25.000 x 109*
AUMENTO DEL PT,<br>(en pacientes que NO reciben anticoagulantes)	1.1 a < 1.25 x LSN	1.25 a < 1.50 x LSN	1.50 a < 3.00 x LSN	≥ 3.00 x LSN
LEUCOPENIA<br>(cel./mm3; *cel./L*)<br>*> 7 días de edad*	2,000 a 2,499<br>*2.000 x 109 a 2.499 x 109*	1,500 a 1,999<br>*1.500 x 109 a 1.999 x 109*	1,000 a 1,499<br>*1.000 x 109 a 1.499 x 109*	< 1,000<br>*< 1.000 x 109*
≤ 7 días de edad	5,500 a 6,999<br>*5.500 x 109 a 6.999 x 109*	4,000 a 5,499<br>*4.000 x 109 a 5.499 x 109*	2,500 a 3,999<br>*2.500 x 109 a 3.999 x 109*	< 2,500<br>*< 2.500 x 109*

17 Sexo masculino o femenino definido al nacimiento.
18 Para la conversión de g/dL a mmol/L se utiliza 0.6206, que es el factor más comúnmente utilizado. Para graduar la hemoglobina con otro factor de corrección que no sea 0.6206, el resultado debe convertirse a g/dL utilizando el factor de corrección que corresponda al laboratorio utilizado.

Orina

PARÁMETRO	**TOXICIDAD GRADO 1 LEVE**	**TOXICIDAD GRADO 2 MODERADO**	**TOXICIDAD GRADO 3 SEVERA**	**TOXICIDAD GRADO 4 CON RIESGO DE VIDA**
GLUCOSURIA (al azar por tirilla reactiva)	Trazos a 1+ o ≤ 250 mg	2+ o > 250 a ≤ 500 mg	> 2+ o > 500 mg	NA
HEMATURIA (no debe reportarse según tirilla reactiva ni si la sangre proviene de sangrado menstrual)	6 a < 10 GR por campo	≥ 10 gr por campo	Macroscópico con o sin coágulos o con cilindros o que requiere una intervención	Con riesgo de vida
PROTEINURIA (al azar por tirilla reactiva)	1+	2+	3+ o más	NA

13.3 Anexo C. Medicaciones no Permitidas

a. Barbitúricos
b. Carbamacepina
c. Oxacarbacepima
d. Fenobarbital
e. Fenitoína
f. Dofetilide
g. Rifampicina
h. Metformina (las concentraciones de metformina pueden aumentar por efecto del dolutegravir; las pacientes deben ser monitoreadas y se debe ajustar la dosis de metformina).
i. Productos que contengan Hierba de San Juan
j. Todas las medicaciones que figuren como no permitidas en el prospecto de las drogas de estudio
k. Metadona (debido a probable disminución de los niveles plasmáticos, las pacientes deben ser monitoreadas por la probabilidad de signos de abstinencia y necesidad de ajuste de la dosis de metadona)
l. Inmunomoduladores: corticosteroides sistémicos, interleuquinas, o interferones
m. Agentes quimioterápicos
n. Vacunas terapéuticas para HIV
o. Todas las drogas en investigación

El uso crónico (oral o parenteral) de glucocorticoides debe ser evitado; sin embargo, el investigador podrá autorizar cursos cortos de tratamiento (por ejemplo 10 días o menos) o el uso tópico, inhalado o intranasal.

13.4 Anexo D. Listado de Manifestaciones Esperables Conocidas de Infección de HIV.

a. Candidiasis:
   - *Bronquial
   - *Esófago
   - *Pulmones
   - Orofaríngea (Aftas)
   - *Tráquea
   - Vulvovaginal (Persistente, Frecuente, o Pobre Respuesta a la Terapia)
   - Otras Candidiasis

b. Citomegalovirus:
   - *Retinitis
   - *Enfermedad por Citomegalovirus (diferentes al hígado, bazo o nódulos linfáticos)

c. Herpes Simplex Virus:
   - *Bronquitis
   - *Esofagitis
   - *Neumonitis
   - *Úlceras Crónicas (>1 mes de duración)
   - Otros Sitios

d. Linfoma (Relacionado con HIV):
   - *Burkitt
   - *Immunoblástico
   - *Primario de Cerebro

e. Enfermedad por Micobacterias:
   - **Mycobacterium avium – intracellulare*
   - **Mycobacterium kansasii*
   - **Mycobacterium tuberculosis*
   - *Otras Micobacteriosis

f. Otros:

- *Cáncer Cervical, invasivo
- Cáncer Cervical, *in situ*
- Displasia Cervical
- *Coccidioidomicosis
- *Criptococcosis
- *Criptosporidiosis
- *Encefalopatía Relacionada con HIV
- Leucoplasia Oral Vellosa
- Herpes zoster
- *Histoplasmosis
- *Isosporidiasis
- *Sarcoma de Kaposi
- Listeriosis
- *Neumonía por *Pneumocystis jirovesi* (PCP)
- *Neumonía, recurrente
- *Leucoencefalopatía Multifocal Progresiva (LMP)
- *Salmonelosis
- *Septicemia, recurrente
- *Toxoplasmosis Cerebral
- *Síndrome de Desgaste, relacionado con HIV

*Eventos que definen SIDA tal como es definido por CDC Surveillance Case Definition of 1993

13.5 Anexo E. Documentos Necesarios Previos a la Iniciación del Estudio

Previo al inicio del estudio clínico, los investigadores deberán proveer la siguiente documentación:

- Original del Acuerdo de Protocolo firmado por el investigador.
- Un *curriculum vitae* actualizado del investigador. Si participaran sub-investigadores en el estudio, se requerirá *curriculum vitae* por cada uno de los individuos adicionales.
- Una copia firmada y fechada de la carta de aprobación del Comité de Ética, en lo que respecta al protocolo, consentimiento informado y cualquier otra información.
- Una lista de los miembros del Comité de Ética, incluyendo sus ocupaciones y sus afiliaciones institucionales.
- Una copia del Consentimiento Informado aprobado por el Comité de Ética que será utilizada en el estudio.
- Una lista de valores y rangos de referencia normales de exámenes de laboratorio especificados en el protocolo para todos los laboratorios a ser utilizados durante el estudio.
- Una copia actualizada de la(s) certificación(es) del laboratorio o del número de certificación, el nombre de la autoridad certificada, período de certificación, y el *curriculum vitae* del Director del Laboratorio.

13.6 Anexo F. Responsabilidades del Centro (Fundación Huésped)

El centro del presente Estudio Clínico es responsable ante las Autoridades de Salud de tomar todos los pasos necesarios para asegurar la realización correcta del Protocolo de Estudio Clínico por lo que respecta a ética, cumplimiento con el Protocolo de Estudio Clínico, e integridad y validez de datos registrados en las Formas para Reporte de Caso. De este modo, el principal deber del Equipo de Monitoreo es ayudar al Investigador y al Patrocinador a mantener un alto nivel de calidad ética, científica, técnica y regulatoria en todos los aspectos del Estudio Clínico.

A intervalos regulares durante el Estudio Clínico, el centro será contactado a través de visitas de monitoreo, cartas o llamadas telefónicas por un representante del Equipo de Monitoreo para revisar el progreso del estudio, el cumplimiento del Investigador y las pacientes con los requisitos del Protocolo de Estudio Clínico y cualquier problema emergente. Durante estas visitas de monitoreo, se revisará, aunque no de forma limitativa, la siguiente lista de puntos: consentimiento informado de la paciente, reclutamiento y seguimiento de la voluntaria, documentación y reporte de Eventos Adversos Serios, reporte de documentación de EA, asignación de Producto de Investigación, cumplimiento de la paciente con el Protocolo de Estudio Clínico y el régimen de Producto de Investigación, contabilidad de Producto de Investigación, uso de terapia concomitante y calidad de datos.

13.7 Anexo G. Responsabilidades del Investigador Clínico

a. Asegurar la aprobación previa del estudio por el Comité de Ética Independiente conforme a las guías ICH.
b. Obtener consentimiento informado válido de la paciente que participa en el estudio.
c. Preparar y mantener en forma adecuada las historias clínicas de todas las personas que están en el estudio, incluyendo CRF, registros de hospitales, resultados de laboratorio, etc., y mantener estos datos en el tiempo requerido por la institución.
d. Identificar todos los sub-investigadores que también supervisen la administración de la droga.
e. Reportar eventos adversos al investigador principal. En el caso de eventos adversos serios o eventos adversos inesperados, notificar al investigador principal en forma inmediata por teléfono.

13.8 Anexo H. Elementos del Consentimiento Informado

El consentimiento firmado debe obtenerse previo a cualquier actividad específica y debe incluir los siguientes ítems:

a. Una declaración que el estudio comprende una investigación, explicación de los objetivos de la investigación y la duración esperable de la participación de las pacientes, descripción de los procedimientos, y la identificación de cualquier procedimiento experimental y/o invasivo.
b. Una descripción del estudio, incluyendo tratamientos y la probabilidad de asignar en forma aleatoria el tratamiento.
c. Una descripción de cualquier riesgo esperable, inconveniencias o discomfort para la paciente.
d. Una descripción de las responsabilidades de la paciente.
e. Una descripción de cualquier beneficio de la paciente o de otras que sería razonablemente esperable de la investigación. Si la paciente recibiera una compensación por su participación, el consentimiento debería describir en qué consiste la compensación (para asegurar que no existe ni coerción ni influencia).
f. Una explicación de procedimientos o tratamientos alternativos, si existe, explicar ventajas, beneficios y riesgos potenciales.
g. Una declaración en la que el investigador y el Comité de Ética garantizarán acceso directo a la historia clínica para la verificación de los procedimientos y datos del estudio, sin violar la confidencialidad de la paciente, en la medida permitida por las leyes y reglamentos aplicables y que, al firmar un formulario de consentimiento informado por escrito, la paciente o representante legalmente aceptable de la paciente, está autorizando tal acceso.
h. Una declaración en la que los registros que identifiquen el tema se mantendrán confidenciales y, en la medida permitida por la legislación aplicable y/o reglamentos, no se harán públicos. Si se publican los resultados de la prueba, la identidad de la paciente se mantendrá confidencial.
i. Una explicación de a quién contactar para obtener respuestas a preguntas pertinentes sobre la investigación y derechos de las pacientes de

investigación, y a quién contactar en caso que la paciente presente alguna lesión o evento relacionado con el estudio. (NOTA: Es preferible identificar como contacto de una persona aquella distinta del investigador. Puede ser necesaria la orientación del Comité de Ética).

j. Una declaración en la que se asegure que la participación es voluntaria, que la negativa a participar no supone ninguna sanción o pérdida de beneficios a los que la paciente tiene derecho, y que la paciente puede interrumpir su participación en cualquier momento sin sanciones ni pérdida de beneficios a los que la paciente está habituada.
k. Una declaración de que un original firmado y fechado del consentimiento informado se dará a la paciente.
l. Una declaración en la que la paciente está de acuerdo en participar (por ejemplo, “Yo estoy de acuerdo en participar...”).
m. Un lugar para la firma y la fecha de la participante de la investigación (o representante legalmente autorizado) y para la persona que explica la naturaleza del estudio a la paciente (representante investigador o investigador).
n. Una declaración que un tratamiento en particular o procedimientos que pueden entrañar riesgos para la paciente son actualmente imprevisibles.
o. Una declaración de las circunstancias previstas o razones en las que la participación de la paciente podrá ser denunciada por el investigador sin tener en cuenta el consentimiento de la paciente.
p. Una declaración de cualquier gasto adicional para la paciente que puede derivarse de la participación en la investigación.
q. Una declaración sobre las consecuencias de la decisión de una paciente de retirarse del estudio y los procedimientos para la finalización ordenada de la participación de la paciente.
r. Una declaración que los nuevos hallazgos significativos desarrollados durante el estudio que podrían referirse a la voluntad de la paciente de continuar su participación, se hará efectiva para la paciente (o su representante legal) de una manera oportuna.

s. Una declaración del número aproximado de pacientes que participan en el estudio.

13.9 Anexo I. Cuestionarios del Estudio

1) CUESTIONARIO SOBRE CONSUMO DE SUSTANCIAS (USO DE DROGAS Y ALCOHOL)

Participante # ____________ **Iniciales** ________ **Visita** ________ **Fecha de Visita** __ __ / __ __ __ / __ __ __ __

Ahora voy a hacerte algunas preguntas respecto al consumo de bebidas alcohólicas desde la última visita médica (fecha___/___/___). Como parte de nuestra evaluación, es importante conocer los estilos de vida que pueden afectar tu salud. Por lo tanto, te pedimos que completes con total honestidad este cuestionario de preguntas sobre el consumo de alcohol. Al momento de responder considera que "una bebida que contiene alcohol" equivale a: Una lata o botella pequeña de cerveza (330 ml) o una copa de vino tinto o blanco o un vaso con 40 ml de pisco, vodka, tequila, whiskey, ron (u otros tipos de tragos fuertes con alcohol).

1. ¿Cada cuánto tomás una bebida que contiene alcohol? (**espontánea una sola respuesta)**

4 veces o más a la semana	4
2-3 veces por semana	3
2-4 veces al mes	2
Una vez al mes	1
Nunca	0
Ns/ Nc	99

2. ¿Cuántos tragos tomás en un día normal cuando estás tomando? (**espontánea una sola respuesta)**

10 o más	4
7 o 9	3
5 o 6	2
3 o 4	1
1 o 2	0
Ns/ Nc	99

3. ¿Con qué frecuencia tomás seis a más tragos en una sola salida? (**espontánea una sola respuesta)**

Casi a diario o todos los días	4
Cada semana	3
Cada mes	2
Menos de una vez al mes	1
Nunca	0
Ns/ Nc	99

Las siguientes preguntas indagan sobre tu posible uso de drogas (excluyendo alcohol y tabaco) desde la última visita. Por favor contestá con honestidad ¨SÍ¨ o ¨NO¨. Cuando se usa la expresión ¨abuso de drogas¨, nos referimos al uso de drogas prescritas por el médico, o las que se compran sin receta, o drogas recreativas. Algunos ejemplos de drogas incluyen pero no se limitan a: cannabis (marihuana.), drogas tranquilizantes (como Valium), barbitúricos, cocaína, estimulantes, alucinógenos (como LSD) o narcóticos (como heroína). Por favor no te olvides que estas preguntas no incluyen el uso de alcohol o tabaco.

4. Desde la última visita (__/__/__), ¿usaste otras drogas diferentes a las requeridas por razones médicas? (**espontánea una sola respuesta)**

SI	1	
NO	0	**Pasa a la 7**
Ns/Nc	9	

5. Desde la última visita, ¿consumiste alguna de estas sustancias? (**espontánea una sola respuesta)**

Alcohol	1
Marihuana	2
Cocaína	3
Crack	4
Anfetaminas/cristal	5
Heroína	6
Pasta base/PACO	7
Clonazepam(no recetado)	8
Ketamina	9
Otros ¿Cuál?	
Nunca	0
Ns/ Nc	99

6. Desde la última visita, ¿Con qué frecuencia consumiste? (**espontánea una sola respuesta)**

Casi a diario o todos los días	4
Cada semana	3
Cada mes	2
Menos de una vez al mes	1
Nunca	0
Ns/ Nc	99

2) CUESTIONARIO SOBRE COMPORTAMIENTO SEXUAL

Participante # _____________ **Iniciales** ________ **Visita** ________ **Fecha de Visita** __ __ / __ __ __ / __ __ __ __

Para esta parte de la encuesta, en la que vamos a hablar de tu vida sexual, la palabra "sexo" significa haber tenido contacto genital con otra persona que puede o no haberte llevado al orgasmo o a tu compañero/a (esto incluye relaciones sexuales anales, orales o vaginales). Sexo NO incluye actividades tales como "sexo por teléfono" o besos, actividades que no involucran contacto genital. Utilizaremos la palabra "parejas sexuales" para referirnos a todas las personas con las que hayas tenido relaciones sexuales (parejas estables, ocasionales o clientes)

Todas las preguntas a continuación hacen referencia a las parejas sexuales en el **último mes**, esto significa desde **___/___/___ (fecha).**

**7.** ¿Cuántas veces has tenido una pareja diferente en el último mes?	Una al mes	Dos al mes o cada dos semanas	Cada semana	Dos a la semana	Tres a la semana	Cada dos días	Diaria	Dos al día	Tres al día	Otro (calculo)
	1	2	4	8	12	15	30	60	90	

Si no recordás el número exacto de parejas, por favor proporcióname el número que más se acerque según tus cálculos. Por ejemplo, si has tenido una pareja nueva cada dos semanas, tu respuesta podría ser dos parejas..." (Continuar usando la escala que se muestra arriba para ayudarte a llegar a un número estimado).

8. ¿Cuántas de estas parejas eran clientes sexuales? **(espontánea una sola respuesta)**

Ns/Nc	99

9. ¿Con cuántas parejas has consumido alcohol/drogas antes o durante una relación sexual? **(espontánea una sola respuesta)**

Ns/Nc	99

10. ¿Con cuántos hombres has tenido sexo en el último mes?

Ns/Nc	99

Si no tuvo relaciones con hombres en el último mes pase a 20

11. ¿Cuántos hombres han eyaculado en tu boca durante el sexo oral en el último mes?

Ns/Nc	99

12. ¿Con cuántos hombres has tenido sexo anal en el último mes?

Ns/Nc	99

13. ¿Con cuántos hombres has tenido sexo anal penetrativo en el último mes (tu pene en su ano; activo; “se la metiste”)?

Ns/Nc	99

14. De estos hombres, ¿con cuántos has tenido sexo anal penetrativo sin preservativo?

Ns/Nc	99

15. ¿Con cuántos hombres has consumido alcohol/drogas antes o durante una relación sexual?

Ns/Nc	99

16. De estos hombres, ¿cuántos fueron :

**VIH+**		**VIH-**		**No se**	
Ns/Nc	99	Ns/Nc	99	Ns/Nc	99

17. ¿Con cuántos hombres has tenido sexo anal receptivo en el último mes (su pene en tu ano; pasivo; “te la metieron”)?

Ns/Nc	99

18. De estos hombres, ¿con cuántos has tenido sexo anal receptivo sin preservativo?

Ns/Nc	99

19. De estos hombres, ¿cuántos fueron :

**VIH+**		**VIH-**		**No se**	
Ns/Nc	99	Ns/Nc	99	Ns/Nc	99

20. ¿Con cuántas mujeres has tenido sexo en el último mes?

Ns/Nc	99

Si no tuvo relaciones con mujeres en el último mes pase a 24

21. ¿Con cuántas mujeres has tenido sexo vaginal o anal en el último mes?

Ns/Nc	99

22. De estas mujeres, ¿con cuantas has tenido sexo vaginal o anal sin preservativo?

Ns/Nc	99

23. De estas mujeres, cuántas fueron:

**VIH+**		**VIH-**		**No se**	
Ns/Nc	99	Ns/Nc	99	Ns/Nc	99

24. ¿Con cuántas **personas trans** has tenido sexo en el último mes?

Ns/Nc	99

Si no tuvo relaciones con personas trans en el último mes pase a 28

25. ¿Con cuántas personas trans has tenido sexo vaginal o anal en el último mes?

Ns/Nc	99

26. De estas personas trans, ¿con cuantas has tenido sexo vaginal o anal sin preservativo?

Ns/Nc	99

27. De estas personas trans, cuántas fueron:

**VIH+**		**VIH-**		**No se**	
Ns/Nc	99	Ns/Nc	99	Ns/Nc	99

28. ¿Utilizaste preservativo en tu **última relación sexual**? **(espontánea una sola respuesta)**

NO	0
SI	1
Ns/Nc	9

29. ¿Cuáles fueron las razones por las que no usaste preservativo en la última relación sexual? **(espontánea puede + de 1 respuesta)**

Porque mi pareja no quiso usarlo	1
Porque yo no quise usarlo	2
Porque le resta sensibilidad a la relación	3
Porque en ese momento no tenía	4
Porque corta el momento de la relación	5
Me encontraba bajo la influencia del alcohol	6
Me encontraba bajo la influencia de alguna droga	7
Porque tengo pareja estable	8
Porque confío en mi pareja	9
Porque mi pareja y yo nos testeamos y no tenemos VIH	10
Porque usamos otro tipo de protección (ej. Banda de látex)	11
Porque el cliente me paga más si no usamos preservativo	12
Porque me incomoda	13
Por descuido	14

Otros :	
Ns/ Nc	99

3) CUESTIONARIO PSICOSOCIAL Basal

Participante # ___________ **Iniciales** ________ **Visita** ________ **Fecha de Visita** __ __ / __ __ __ / __ __ __ __

DEMOGRÁFICAS

Vamos a iniciar la entrevista preguntándote

1. ¿Qué edad tenés? **(espontánea una sola respuesta) Edad en años cumplidos**

Ns/Nc	99

2. ¿Cuál es tu país de nacimiento? **(espontánea una sola respuesta)**

Argentina	1
País limítrofe (Uruguay, Brasil, Chile, Paraguay o Bolivia)	2
Otro país Sudamericano	3
Otro país centroamericano	4
Otros país de América del Norte (EEUU; Canadá o México)	5
Otro país Europeo	6
Otro país Africano	7
Otro país Asiático	8
Ns/ Nc	9

3. ¿Cuál es tu lugar de residencia actual? **(espontánea una sola respuesta)**

Ciudad de Bs As	1
Conurbano Bs As	2
Ns/ Nc	99

4. ¿Cuál es el último nivel al que asistís o asististe del ciclo educativo? **(lea las alternativas una sola respuesta)**

Primario/ EGB incompleto	1	Secundario/Polimodal completo	4
Primario/EGB completo	2	Terciario o Universitario incompleto	5
Secundario/Polimodal incompleto	3	Terciario o Universitario Completo	6
		Ns/ Nc	9

5. ¿Realizaste alguna vez o realizas trabajo sexual? **(lea las alternativas una sola respuesta)**

NO	0
Si, actualmente	1
Sí, pero no es este momento	2
Ns/ Nc	9

6. ¿Realizas otro trabajo que no sea sexual? **(lea las alternativas una sola respuesta)**

Si	1	→ **Sigue a 8**
No	0	
Ns/ Nc	9	

7. ¿Buscaste trabajo en los últimos 30 días? **(espontánea una sola respuesta)**

SI	1
NO	0
Ns/nc	9

Sigue a 12

8. ¿Cuál es **tu situación ocupacional principal**? **(lea las alternativas una sola respuesta)**

Soy patrón o empleador	1
Trabajo por cuenta propia/ autónomo	2
Trabajo en relación de dependencia	3
Tengo un trabajo familiar sin remuneración	4
Ns/ Nc	9

9. En tu **trabajo (no trabajo sexual)**; ¿cuántas horas trabajas por semana habitualmente? **(espontánea una sola respuesta)**

Menos de 35 hs	2
35 hs o más	1
Ns/ Nc	9

10. ¿Estás dispuesta a trabajar más horas de las que trabajas habitualmente? **(lea las alternativas una sola respuesta)**

NO	0
Sí; busca pero no encuentro	1
Sí; pero no busco	2
Ns/ Nc	9

11. ¿En tu trabajo te realizan aportes jubilatorios? (incluye monotributo social) **(espontánea una sola respuesta)**

SI	1
NO	0
Ns/Nc	9

12. ¿Tenés alguna discapacidad física de cualquier tipo? **(espontánea una sola respuesta)**

13. ¿Tenés certificado de discapacidad? **(espontánea una sola respuesta)**

14. ¿Tenés pensión por discapacidad? **(espontánea una sola respuesta)**

	12
SI	1
No	0
Ns/Nc	9

	13
SI	1
No	0
Ns/Nc	9

	14
SI	1
No	0
Ns/Nc	9

VIVIENDA

15. ¿Cuál es tu tipo de vivienda? **(lea las alternativas una sola respuesta)**

Casa	1	Pieza de hotel fam/ pensión	6
Rancho	2	Local no construido p/ habit	7
Casilla	3	Vivienda móvil	8
Departamento	4	Situación de calle	10
Pieza de inquilinato	5	Ns/ Nc	9

16. En tu baño; ¿tenés inodoro con descarga de agua? **(espontánea una sola respuesta)**

SI	1

NO	0
Ns/Nc	9

17. ¿Cuál es el régimen de tenencia de tu vivienda? **(lea las alternativas una sola respuesta)**

Propietario	1	Usurpante (okupas)	4
Inquilino	2	Otros **(No leer)**	5
Ocupante gratuito	3	Ns/ Nc	9

18. Incluyéndote a vos misma; ¿cuántas personas viven en tu hogar (o habitación de hotel/pensión)? **(También incluyendo el personal doméstico si lo tiene) (espontánea una sola respuesta)**

Ns/Nc	99

19. ¿Cuál es el ingreso mensual **personal**? **(guiada simple)**

**<2500**	1
**2500 a 5000**	2
**5000 a 10000**	3
**10000 a 20000**	4
**>20000**	5
Ns/ Nc	9999

20. ¿Cuál es el ingreso mensual del grupo familiar o el grupo con el que vivís? **(Una sola respuesta. Todos los ingresos sumados de las personas que viven en un mismo hogar)**

**<2500**	1
**2500 a 5000**	2
**5000 a 10000**	3
**10000 a 20000**	4
**>20000**	5
Ns/ Nc	9999

21. ¿Cuántos niños/as (de 0 a 18 años) conviven con vos? **(Si es ninguno poner 0) (espontánea una sola respuesta)**

Ns/Nc	99

22. ¿Cuántos niños/as (de 0 a 18 años) dependen de vos económicamente? **(Si es ninguno poner 0) (espontánea una sola respuesta)**

Ns/Nc	99

ESTIGMA Y DISCRIMINACION POR IDENTIDAD TRANS EN DIFERENTES AMBITOS

SALUD

Ahora voy a hacerte una serie de preguntas sobre tu experiencia en los servicios de salud en relación a tu identidad trans

23. Por tu identidad de trans ¿en el último año viviste experiencias de discriminación en un hospital o centro privado por parte las personas que voy a nombrarte? **(lea las alternativas puede + de 1 respuesta)**

Médicos	1
Personal administrativo	2
Enfermero	3
Otros pacientes	4
Otros profesionales (servicio social, psicólogos)	5
Otra ¿Cuál?	
Ninguna	0
Ns/ Nc	99

24. Por tu identidad trans ¿en el último año viviste experiencias de discriminación como las que voy a nombrarte? **(lea las alternativas puede + de 1 respuesta)**

Evitaste ir a un centro privado/ hospital	1
No te atendieron en un servicio de salud/ no respetaron tu turno	2
No te llamaron por tu nombre de elección	3
El personal de servicio se burló de vos o te agredió	4
El personal del servicio te trató prejuiciosamente o de forma despectiva	5
El personal de salud hizo insinuaciones sexuales	6
Te internaron junto con varones	7
Preferiste pagar la atención de salud en un centro privado	8
Otra ¿Cuál?	
Ninguna	0
Ns/ Nc	99

EDUCACION

Ahora voy a hacerte una serie de preguntas sobre educación

25. ¿Durante el último año estuviste estudiando, retomaste o intentaste retomar los estudios? **(espontánea una sola respuesta)**

Estoy estudiando desde hace más de 1 año	1	
Retome los estudios en el último año	2	
Intenté retomar los estudios en el último año	3	
NO	0	Sigue a 28
Ns/nc	9	Sigue a 28

26. Por tu identidad de trans ¿en el último año viviste experiencias de discriminación en un centro educativo por parte de las personas que voy a nombrarte? **(lea las alternativas puede + de 1 respuesta)**

Directores/as	1
Maestros o profesores	2
Personal no docente (preceptor, portero, etc.)	3
Compañeros de estudio	4
Otros profesionales (psicopedagogos, psicólogos)	5
Otra ¿Cuál?	
Ninguna	0

Ns/ Nc	99

27. Por tu identidad trans ¿en el último año te ocurrió alguna de las siguientes situaciones que voy a mencionarte? **(lea las alternativas puede + de 1 respuesta)**

Te negaron matricularte o no te dejaron acceder a clases	1
Te sentiste forzada a abandonar tu educación y/o capacitación	2
No aprovechaste una oportunidad educativa y/o capacitación	3
El personal docente se burló/ te agredió	4
No te llamaron por tu nombre de elección	5
Te negaron el acceso a los baños	6
Maltrato de compañeros (bullying)	7
Otras:	
Ninguna de estas	0
Ns/ Nc	99

TRABAJO

Ahora voy a hacerte una serie de preguntas sobre tu trabajo

A LAS QUE REALIZAN O REALIZARON TRABAJOS DIFERENTES AL TRABAJO SEXUAL

28. Por tu identidad trans ¿en el último año sentiste rechazada o viviste experiencias de discriminación en el trabajo por parte de las personas que voy a nombrarte? **(lea las alternativas puede + de 1 respuesta)**

Jefes/directivos	1
Compañeros de trabajo	2
Personal de otras áreas	3
Clientes/proveedores	4
Otra ¿Cuál?	
Ninguna	0
Ns/ Nc	99

A LAS QUE REALIZAN O REALIZARON TRABAJO SEXUAL

29. Por tu identidad trans ¿en el último año sentiste rechazo o viviste experiencias de discriminación en el trabajo por parte de las personas que voy a nombrarte? **(lea las alternativas puede + de 1 respuesta)**

Cliente sexual	1
De la persona que te regentea	2
Otras trabajadoras sexuales	3
Vecinos/as de la zona	4
Dueño/operador del lugar donde trabajo	5
Otra ¿Cuál?	
Ninguna	0
Ns/ Nc	99

A TODAS

30. Por tu identidad trans ¿en el último año te ocurrió alguna de las siguientes situaciones que voy a mencionarte? **(lea las alternativas puede + de 1 respuesta)**

Te negaron un trabajo	1
Tuviste que dejar un trabajo	2
Decidiste no solicitar un empleo/ trabajo o ascenso laboral	3

No te llamaron por tu nombre de elección	4
Te negaron el acceso a los baños	5
Tus compañeros de trabajo se burlaron o te amenazaron	6
Un cliente te amenazó, insultó, humilló o gritó de una manera que te hiciera sentir miedo	7
Un cliente te golpeó, pateó, empujó o lastimó físicamente	8
Un cliente te obligó a tener relaciones sexuales en contra de tu voluntad	9
Otros:	
Ninguna	0
Ns/ Nc	99

VIVIENDA

Ahora voy a hacerte una serie de preguntas sobre el lugar donde vivís

31. Por tu identidad trans ¿en el último año te ocurrió alguna de las siguientes situaciones que voy a mencionarte vinculadas a la vivienda? **(lea las alternativas puede + de 1 respuesta)**

Tus vecinos te insultaron **verbalmente**, te acosaron, y/o amenazaron	1
Tus vecinos te acosaron, amenazaron y/o agredieron **físicamente**	2
Los vecinos hicieron una denuncia en tu contra	3
Te negaron un alquiler o la compra de una vivienda	4
Te cobraron más de alquiler	5
Fuiste forzada a cambiar tu lugar de residencia	6
Otros:	
Ninguna	0
Ns/ Nc	99

FUERZAS DE SEGURIDAD

Ahora voy a hacerte una serie de preguntas en relación a las Fuerzas de Seguridad (policía, gendarmería, etc.)

32. Por tu identidad trans ¿en el último año vez fuiste detenida por la policía? (espontánea una sola respuesta)

SI	1	
NO	0	**Sigue a 35**
Ns/nc	9	**Sigue a 35**

33. Si fuiste detenida ¿cuál fue la razón? **(lea las alternativas puede + de 1 respuesta)**

Averiguación de antecedentes (“portación de cara”)	1
Prostitución (Contravencionales, edictos, “vestirse de mujer”, etc.)	2
Delitos relacionados con drogas	3
Merodeo/ “movimientos sospechosos”	4
Pelea callejera/Agresión en la vía pública	5
Resistencia a la autoridad	6
Homicidio	7
Hurto	8
Por no tener en regla los papeles del auto/moto	9
Tenencia de dinero falso	10
Otro:	
Ninguna	0
Ns/ Nc	99

34. Por tu identidad trans ¿en el último año te ocurrió alguna de las siguientes situaciones que voy a mencionarte**? (lea las alternativas puede + de 1 respuesta)**

Te mantuvieron detenida más tiempo que a otras personas no trans	1
Te privaron de algún derecho (como llamar a un abogado)	2
Te ubicaron en una celda junto con varones	3
Te realizó una requisa un varón	4
Un policía te amenazó, insultó, humilló o gritó de una manera que te hiciera sentir miedo	5
Un policía te golpeó, pateó, empujó o lastimó físicamente	6
Un policía te obligó a tener relaciones sexuales en contra de tu voluntad	7
No te permitieron que te revisara un médico en caso de golpes o heridas	8
Otros:	
Ninguna	0
Ns/ Nc	99

SOCIAL (VECINOS/FAMILIA)

Ahora voy a hacerte una serie de preguntas sobre tu vida cotidiana y situaciones sociales en relación a tu identidad trans

35. ¿En tu vida cotidiana, con qué frecuencia te suceden las siguientes situaciones por ser una mujer trans?

	Nunca	A veces	Normalmente	A menudo	Siempre
35.1. Te tratan con menos cortesía	1	2	3	4	5
35.2. Te tratan con menos respeto	1	2	3	4	5
35.3. Recibís un servicio menor al adecuado	1	2	3	4	5
35.4. La gente actúa como si te tuvieran miedo	1	2	3	4	5
35.5. La gente actúa como si no fueras inteligente	1	2	3	4	5
35.6. La gente actúa como si fueras deshonesta	1	2	3	4	5
35.7. La gente actúa como si fueran mejores que vos	1	2	3	4	5
35.8. Te insultan	1	2	3	4	5
35.9. Te amenazan o acosan verbalmente	1	2	3	4	5
35.10. Te hacen insinuaciones sexuales (en el trasporte público o en la calle)	1	2	3	4	5

36. Por tu identidad trans ¿en el último año con qué frecuencia te ocurrió alguna de las siguientes situaciones que voy a mencionarte? **(lea las alternativas puede + de 1 respuesta)**

	Nunca	A veces	Normalmente	A menudo	Siempre
36.1. Fuiste excluida de reuniones o actividades sociales (ej. bodas, funerales, fiestas, clubes)	1	2	3	4	5
36.2. Fuiste excluida de actividades religiosas o de lugares de culto	1	2	3	4	5
36.3. Fuiste excluida de actividades familiares (ej: cocinar, comer juntos, dormir en la misma habitación)?	1	2	3	4	5

36.4. Tus familiares te insultaron **verbalmente**, te acosaron, y/o amenazaron	1	2	3	4	5
36.5. Tus familiares te acosaron, amenazaron y/o agredieron **físicamente**	1	2	3	4	5
36.6. Sufriste violencia psicológica por parte de una pareja sexual (NO CLIENTE)	1	2	3	4	5
36.7. Sufriste violencia física por parte de una pareja sexual (NO CLIENTE)	1	2	3	4	5
36.8. Sufriste violencia sexual por parte de una pareja sexual (NO CLIENTE)	1	2	3	4	5
36.9. Experimentaste rechazo sexual por parte de una potencial pareja como resultado de tu identidad trans	1	2	3	4	5
36.10. Tu pareja o alguno de los miembros de tu grupo familiar experimentó discriminación como resultado de tu identidad trans	1	2	3	4	5

37. Por tu identidad trans ¿con que frecuencia sentiste alguna de las emociones que voy a mencionarte**? (lea las alternativas puede + de 1 respuesta)**

	Nunca	A veces	Normalmente	A menudo	Siempre
37.1. Te sentiste avergonzada	1	2	3	4	5
37.2. Te sentiste culpable	1	2	3	4	5
37.3. Con baja autoestima	1	2	3	4	5
37.4. Sentís que deberías ser castigada	1	2	3	4	5
37.5. Tuviste ideas suicidas	1	2	3	4	5

38. ¿Alguna vez intestaste suicidarte? (espontánea una sola respuesta)

SI	1
NO	0
Ns/Nc	9

SI → 39; NO, Ns/Nc ↓

39. ¿A qué edad fue el primer intento de suicidio? (espontánea una sola respuesta)

Ns/Nc	999

40. Por tu identidad trans ¿en el último año te ocurrió alguna de las siguientes situaciones que voy a mencionarte?
(lea las alternativas puede + de 1 respuesta)

	Nunca	A veces	Normalmente	A menudo	Siempre
40.1. Preferiste no asistir a reuniones sociales	1	2	3	4	5
40.2. Te aislaste de tu familia	1	2	3	4	5
40.3. Te aislaste de tus amigos	1	2	3	4	5
40.4 Dejaste de tomar transporte público	1	2	3	4	5
40.5 No volviste a tu ciudad/pueblo de origen	1	2	3	4	5

41. Por tu identidad trans ¿en el último año tuviste miedo a que te ocurrieran alguna siguientes situaciones que voy a mencionarte (ocurrieran o no)? **(lea las alternativas puede + de 1 respuesta)**

	Nunca	A veces	Normalmente	A menudo	Siempre
41.1. Temor a ser blanco de murmuraciones	1	2	3	4	5
41.2. Temor a ser insultada, acosada y/o	1	2	3	4	5

amenazada verbalmente					
41.3. Temor a ser agredida físicamente	1	2	3	4	5
41.4. Temor a que alguien no quiera entablar una relación sexual íntima con vos debido a su identidad trans	1	2	3	4	5

TRAMITES SISTEMA PÚBLICO

Ahora voy a hacerte una serie de preguntas sobre tramites en el Sistema Público (AFIP, ANSES, registro civil,etc.)

42. Por tu identidad trans ¿en el último año te ocurrió alguna siguientes situaciones que voy a mencionarte? **(lea las alternativas puede + de 1 respuesta)**

Te negaron realizar un trámite en una institución pública (no de salud ni educación, ej. ANSES, AFIP, registro civil, etc.)	1
Decidiste no realizar un trámite en una institución pública	2
No te llamaron por tu nombre de elección en una institución pública	3
El personal de la institución pública se burló de vos o insultó	4
Otras personas que estaban en la institución pública se burlaron o te insultaron	5
Ninguna	0
Ns/ Nc	99

DISCRIMINACION POR VIH

Ahora voy a hacerte una serie de preguntas sobre tus experiencias en los servicios de salud en relación a tu diagnóstico de VIH

43. Por tener VIH, ¿alguna vez viviste experiencias de discriminación en un hospital o centro privado por parte de las personas que voy a nombrarte? **(lea las alternativas puede + de 1 respuesta)**

Médico	1
Personal administrativo	2
Enfermeros	3
Otros pacientes	4
Otros profesionales (servicio social, psicólogos)	5
Otra persona trans	6
Otra ¿Cuál?	
Ninguna	0
Ns/ Nc	99

CONSUMO DE SUSTANCIAS

Ahora voy a hacerte algunas preguntas respecto al consumo de bebidas alcohólicas en el último año (12 meses). Como parte de nuestra evaluación, es importante conocer los estilos de vida que pueden afectar tu salud. Por lo tanto, te pedimos que completes con total honestidad este cuestionario de preguntas sobre el consumo de alcohol en el último año. Al momento de responder considera que “una bebida que contiene alcohol” equivale a: Una lata o botella pequeña de cerveza (330 ml) o una copa de vino tinto o blanco o un vaso con 40 ml de pisco, vodka, tequila, whiskey, ron (u otros tipos de tragos fuertes con alcohol).

44. ¿Cada cuánto tomás una bebida que contiene alcohol? (**espontánea una sola respuesta)**

4 veces o más a la semana	4
2-3 veces por semana	3

2-4 veces al mes	2
Una vez al mes	1
Nunca	0
Ns/ Nc	99

45. ¿Cuántos tragos tomás en un día normal cuando estás tomando? (**espontánea una sola respuesta)**

10 o más	4
7 o 9	3
5 o 6	2
3 o 4	1
1 o 2	0
Ns/ Nc	99

46. ¿Con qué frecuencia tomás seis a más tragos en una sola salida? (**espontánea una sola respuesta)**

Casi a diario o todos los días	4
Cada semana	3
Cada mes	2
Menos de una vez al mes	1
Nunca	0
Ns/ Nc	99

47. ¿Con qué frecuencia durante **el último año** no pudiste dejar de tomar una vez que empezaste a hacerlo? (**espontánea una sola respuesta)**

Casi a diario o todos los días	4
Cada semana	3
Cada mes	2
Menos de una vez al mes	1
Nunca	0
Ns/ Nc	99

48. ¿Con qué frecuencia durante **el último año**, dejaste de hacer algo que normalmente esperarías hacer a causa de la bebida? (**espontánea una sola respuesta)**

Casi a diario o todos los días	4
Cada semana	3
Cada mes	2
Menos de una vez al mes	1
Nunca	0
Ns/ Nc	99

49. ¿Con qué frecuencia durante **el último año**, necesitaste de un trago en la mañana para reponerte después de una buena borrachera? (**espontánea una sola respuesta)**

Casi a diario o todos los días	4
Cada semana	3
Cada mes	2
Menos de una vez al mes	1
Nunca	0
Ns/ Nc	99

50. ¿Con qué frecuencia durante **el último año** te sentiste culpable o con remordimientos después de tomar? (**espontánea una sola respuesta)**

Casi a diario o todos los días	4
Cada semana	3
Cada mes	2

Menos de una vez al mes	1
Nunca	0
Ns/ Nc	99

51. ¿Con qué frecuencia durante **el último año** fuiste incapaz de recordar lo que pasó la noche anterior a causa de la bebida? (**espontánea una sola respuesta)**

Casi a diario o todos los días	4
Cada semana	3
Cada mes	2
Menos de una vez al mes	1
Nunca	0
Ns/ Nc	99

52. ¿Vos o alguien resultó herido o maltratado como resultado de que estabas tomando alcohol? ¿en el último año? (**espontánea una sola respuesta)**

Sí, pero no en el último año	4
Sí, durante el último año	2
No	0
Ns/ Nc	99

53. ¿Algún pariente, amigo, doctor o trabajador de salud ha estado preocupado por lo que tomás o te ha sugerido dejar de tomar? ¿en el último año? (**espontánea una sola respuesta)**

Sí, pero no en el último año	4
Sí, durante el último año	2
No	0
Ns/ Nc	99

54. ¿Algún doctor o trabajador de salud no te atendió porque habías tomando alcohol? ¿en el último año? (**espontánea una sola respuesta)**

Sí, pero no en el último año	4
Sí, durante el último año	2
No	0
Ns/ Nc	99

55. ¿Alguna vez dejaste de ir a consultar a un hospital/centro de salud o retirar medicación porque habías tomando alcohol? ¿en el último año? (**espontánea una sola respuesta)**

Sí, pero no en el último año	4
Sí, durante el último año	2
No	0
Ns/ Nc	99

56. En general, con quien tomas alcohol: **(espontánea más de una respuesta)**

Sola	0
Clientes sexuales	1
Pareja	2
Compañeras trans	3
Discos/Boliches	4
Otros:	
Ns/ Nc	99

Las siguientes preguntas indagan sobre tu posible uso de drogas (excluyendo alcohol y tabaco) durante el último año (12 meses). Por favor contestá con honestidad ¨SÍ¨ o ¨NO¨. Cuando se usa la expresión ¨abuso de drogas¨, nos referimos al uso de drogas prescritas por el médico, o las que se compran sin receta, o drogas recreativas. Algunos ejemplos de drogas incluyen pero no se limitan a: cannabis (marihuana.), drogas tranquilizantes (como Valium), barbitúricos, cocaína, estimulantes, alucinógenos (como LSD) o narcóticos (como heroína). Por favor no te olvides que estas preguntas no incluyen el uso de alcohol o tabaco.

57. Alguna vez en tu vida, ¿consumiste alguna de estas sustancias? (**espontánea una sola respuesta)**

Marihuana	1
Cocaína	2
Crack	3
Anfetaminas/cristal	4
Heroína	5
Pasta base/PACO	6
Clonazepam, alprazolam, diazepam, lorazepam, etc. (no recetado)	7
Ketamina	8
Otros ¿Cuál?	
Nunca	0
Ns/ Nc	99

58. En el último año ¿usaste otras drogas diferentes a las requeridas por razones médicas? (**espontánea una sola respuesta)**

SI	1
NO	0
Ns/Nc	9

NO / Ns/Nc → **Pasa a Comportamiento sexual**

59. En el último año, ¿consumiste alguna de estas sustancias? (**espontánea una sola respuesta)**

Marihuana	1
Cocaína	2
Crack	3
Anfetaminas/cristal	4
Heroína	5
Pasta base/PACO	6
Clonazepam(no recetado)	7
Ketamina	8
Otros ¿Cuál?	
Nunca	0
Ns/ Nc	99

60. En el último año ¿Cuantos días consumiste drogas? (**espontánea una sola respuesta)**

7 días o más	2
Menos de 7 días	1
Nunca	0
Ns/ Nc	99

→ **Si es menor a 7 pasa a Comportamiento sexual**

61. En el último año ¿Con qué frecuencia consumiste? (**espontánea una sola respuesta)**

Casi a diario o todos los días	4
Cada semana	3
Cada mes	2
Menos de una vez al mes	1
Nunca	0
Ns/ Nc	99

62. En el último año ¿Cuantos días consumiste más de lo que tenías intención de consumir? (**espontánea una sola respuesta)**

2 o más días	2
Menos de 2 días	1
Nunca	0
Ns/ Nc	99

Si es menor a 2 pasa a Comportamiento sexual

63. En el último año, ¿abusaste de más de una droga a la vez? (**espontánea una sola respuesta)**

SI	1
NO	0
Ns/Nc	9

64. En el último año, ¿pudiste dejar de usar las drogas cuando querías? (**espontánea una sola respuesta)**

SI	1
NO	0
Ns/Nc	9

65. En el último año, ¿tuviste una pérdida de conocimiento o una escena retrospectiva (o sea a retrocedido a vivencias del pasado) por causa de las drogas? (**espontánea una sola respuesta)**

SI	1
NO	0
Ns/Nc	9

66. En el último año, ¿te sentiste a veces mal o culpable sobre tu uso de drogas? (**espontánea una sola respuesta)**

SI	1
NO	0
Ns/Nc	9

67. En el último año, ¿tu pareja (o padres) se quejaron de tu uso de drogas? (**espontánea una sola respuesta)**

SI	1
NO	0
No tengo familia ni pareja	8
Ns/Nc	9

68. En el último año, ¿descuidaste a tu familia/pareja por usar drogas? (**espontánea una sola respuesta)**

SI	1
NO	0
No tengo familia ni pareja	8
Ns/Nc	9

69. En el último año, ¿te involucraste en actividades ilegales para conseguir las drogas? (**espontánea una sola respuesta)**

SI	1
NO	0
Ns/Nc	9

70. En el último año, ¿tuviste síndrome de abstinencia (te sentiste enferma) cuando dejaste de tomar drogas? (**espontánea una sola respuesta)**

SI	1
NO	0
Ns/Nc	9

71. En el último año, ¿tuviste problemas médicos por el uso de drogas (por ejemplo, una pérdida de memoria, hepatitis, convulsiones, pérdida de sangre, etc.)? (**Espontánea una sola respuesta)**

SI	1
NO	0
Ns/Nc	9

72. En general, con quien consumís drogas:

Sola	0
Clientes sexuales	1
Pareja	2
Compañeras trans	3
Discos/Boliches	4
Otros:	
Ns/ Nc	99

COMPORTAMIENTO SEXUAL

Para esta parte de la encuesta, en la que vamos a hablar de tu vida sexual, la palabra "sexo" significa haber tenido contacto genital con otra persona que puede o no haberte dado placer o a tu compañero/a (esto incluye relaciones sexuales anales, orales o vaginales). Sexo NO incluye actividades tales como "sexo por teléfono" o besos, actividades que no involucran contacto genital. Utilizaremos la palabra "parejas sexuales" para referirnos a todas las personas con las que hayas tenido relaciones sexuales (parejas estables, ocasionales o clientes)

73. ¿A qué edad tuviste tu primera relación sexual? **(espontánea una sola respuesta) Edad en años cumplidos**

Ns/Nc	99

74. ¿Consentiste esta primera relación sexual (no en contra de tu voluntad)? **(espontánea una sola respuesta)**

SI	1
NO	0
Ns/Nc	9

75. En general, en dónde conocés a tus parejas sexuales:

Espacios públicos durante el trabajo sexual (calle, parque, etc.)	1
Departamento privado (durante el trabajo sexual)	2
Bares	3
Discos/Boliches Gays	4
Saunas/ Cines Pornos	5
Dark Rooms	6
Baños Públicos	7
Chat en Internet	8
Otros:	
Ns/ Nc	99

Todas las preguntas a continuación hacen referencia a las parejas sexuales en el **último mes**, esto significa desde ___/___/___ **(fecha). Si no te acordás el número exacto de parejas, por favor proporcióname el número que más se acerque según tus cálculos.**

76. ¿Cuántas veces has tenido una pareja diferente en el último mes? (hombres, mujeres, trans e incluye sexo oral)

Ninguna	1 al mes	2 al mes	1 cada semana	2 a la semana	3 a la semana	1 cada 2 días	1 al día	2 al día	3 al día	4 al día
0	1	2	4	8	12	15	30	60	90	120

5 al día	6 al día	7 al día	8 al día	9 al día	10 al día	11 al día	12 Al día	13 Al día	15 Al día	Otro calculo
150	180	210	240	270	300	330	360	390	450	

77. ¿Cuántas de estas parejas eran clientes sexuales? (hombres, mujeres, trans e incluye sexo oral) **(espontánea una sola respuesta)**

Ns/Nc	99

78. ¿Con cuántas parejas has consumido alcohol/drogas antes o durante una relación sexual? (hombres, mujeres, trans e incluye sexo oral) **espontánea una sola respuesta)**

Ns/Nc	99

79. ¿Con cuántos hombres (clientes o no) has tenido sexo en el último mes? **espontánea una sola respuesta)**

Ns/Nc	99

Si no tuvo relaciones con hombres en el último mes pase a 89

80. ¿Con cuántos hombres has consumido alcohol/drogas antes o durante una relación sexual? **(espontánea una sola respuesta)**

Ns/Nc	99

81. ¿Cuántos hombres han eyaculado en tu boca durante el sexo oral en el último mes? **(espontánea una sola respuesta)**

Ns/Nc	99

82. ¿Con cuántos hombres has tenido sexo anal en el último mes? (relaciones donde tu rol fue activo y/o pasivo) **(espontánea una sola respuesta)**

Ns/Nc	99

83. ¿Con cuántos hombres has tenido sexo anal penetrativo (tu pene en su ano, activo; “se la metiste”) en el último mes? **(espontánea una sola respuesta)**

Ns/Nc	99

84. De estos hombres, ¿con cuántos has tenido sexo anal penetrativo (activo) sin preservativo? **(espontánea una sola respuesta)**

Ns/Nc	99

85. De estos hombres con los que tuviste sexo penetrativo (activo), ¿cuántos fueron : **(espontánea una sola respuesta)**

**VIH+**		**VIH-**		**No se**	
Ns/Nc	99	Ns/Nc	99	Ns/Nc	99

86. ¿Con cuántos hombres has tenido sexo anal receptivo (su pene en tu ano; pasivo; "te la metieron") en el último mes? **(espontánea una sola respuesta)**

Ns/Nc	99

87. De estos hombres, ¿con cuántos has tenido sexo anal receptivo (pasivo) sin preservativo? **(espontánea una sola respuesta)**

Ns/Nc	99

88. De estos hombres con los que tuviste sexo receptivo (pasivo), ¿cuántos fueron : **(espontánea una sola respuesta)**

**VIH+**		**VIH-**		**No se**	
Ns/Nc	99	Ns/Nc	99	Ns/Nc	99

89. ¿Con cuántas mujeres (clientes o no) has tenido sexo en el último mes? **(espontánea una sola respuesta)**

Ns/Nc	99

Si no tuvo relaciones con mujeres en el último mes pase a 93

90. ¿Con cuántas mujeres has tenido sexo vaginal o anal en el último mes? **(espontánea una sola respuesta)**

Ns/Nc	99

91. De estas mujeres, ¿con cuantas has tenido sexo vaginal o anal sin preservativo? **(espontánea una sola respuesta)**

Ns/Nc	99

92. De estas mujeres, cuántas fueron: **(espontánea una sola respuesta)**

**VIH+**		**VIH-**		**No se**	
Ns/Nc	99	Ns/Nc	99	Ns/Nc	99

93. ¿Con cuántas **personas trans** (clientes o no) has tenido sexo en el último mes? **(espontánea una sola respuesta)**

Si no tuvo relaciones con personas trans en el último mes pase a 97

Ns/Nc	99

94. ¿Con cuántas personas trans has tenido sexo vaginal o anal en el último mes? **(espontánea una sola respuesta)**

Ns/Nc	99

95. De estas personas trans, ¿con cuantas has tenido sexo vaginal o anal sin preservativo? **(espontánea una sola respuesta)**

Ns/Nc	99

96. De estas personas trans, cuántas fueron: **(espontánea una sola respuesta)**

**VIH+**		**VIH-**		**No se**	
Ns/Nc	99	Ns/Nc	99	Ns/Nc	99

97. ¿Actualmente tenés una pareja sexual estable (persona con quien tenés relaciones sexuales frecuentemente y una relación de compromiso mutuo)? **(espontánea una sola respuesta)**

	**97**
SI	1
NO	0
Ns/ Nc	9

NO / Ns/ Nc: **Pasar a 101**

SI → **98.** ¿Cuánto tiempo (en meses) hace que tenés esta pareja? **(espontánea una sola respuesta)**

	**ESCRIBIR un Nº**
Ns/ Nc	99

99. ¿Cuál es el género de tu actual pareja sexual estable? **(lea las alternativas una sola respuesta)**

Mujer	1
Varón	2
Trans	3
Ns/ Nc	9

100. Al momento de tener relaciones sexuales con tu pareja, ¿con qué frecuencia utilizan preservativos? **(lea las alternativas una sola respuesta)**

Siempre	3
La mayoría de veces	2
La minoría de veces	1
Nunca	0
Ns/ Nc	9

101. ¿Utilizaste preservativo en tu **última relación sexual**? **(espontánea una sola respuesta)**

NO	0
SI	1
Ns/Nc	9

102. ¿Cuáles fueron las razones por las que no usaste preservativo en la última relación sexual? **(espontánea puede + de 1 respuesta)**

Porque mi pareja/cliente no quiso usarlo	1

Porque yo no quise usarlo	2
Porque le resta sensibilidad a la relación	3
Porque en ese momento no tenía	4
Porque corta el momento de la relación	5
Me encontraba bajo la influencia del alcohol	6
Me encontraba bajo la influencia de alguna droga	7
Porque tengo pareja estable	8
Porque confío en mi pareja	9
Porque mi pareja y yo tenemos VIH	10
Porque tomo medicación para el VIH y estoy indetectable	11
Porque usamos otro tipo de protección (ej. Banda de látex)	12
Porque el cliente me paga más si no usamos preservativo	13
Porque me incomoda	14
Por descuido	15
Otros :	
Ns/ Nc	99

4) CUESTIONARIO PSICOSOCIAL 6 y 12 meses

Participante # ____________ **Iniciales** _______ **Visita** ______ **Fecha de Visita** __ __ **/** __ __ __ **/** __ __ __ __

DEMOGRÁFICAS

Vamos a iniciar la entrevista preguntándote

1. ¿Cambió tu situación laboral en los últimos 6 meses? **(espontánea una sola respuesta)**

SI	1
NO	0
Ns/nc	9

Sigue a 12

2. ¿Realizas trabajo sexual actualmente? **(lea las alternativas una sola respuesta)**

NO	0
Si, actualmente	1
Sí, pero no es este momento	2
Ns/ Nc	9

3. ¿Realizas otro trabajo que no sea sexual? **(lea las alternativas una sola respuesta)**

Si	1
No	0
Ns/ Nc	9

Sigue a 5

4. ¿Buscaste trabajo en los últimos 30 días? **(espontánea una sola respuesta)**

SI	1
NO	0
Ns/nc	9

Sigue a 9

5. ¿Cuál es **tu situación ocupacional principal**? **(lea las alternativas una sola respuesta)**

Soy patrón o empleador	1
Trabajo por cuenta propia/ autónomo	2
Trabajo en relación de dependencia	3
Tengo un trabajo familiar sin remuneración	4
Ns/ Nc	9

6. En tu **trabajo (no trabajo sexual)**; ¿cuántas horas trabajas por semana habitualmente? **(espontánea una sola respuesta)**

Menos de 35 hs	2
35 hs o más	1

7. ¿Estás dispuesta a trabajar más horas de las que trabajas habitualmente? **(lea las alternativas una sola respuesta)**

NO	0
Sí; busca pero no encuentro	1

Ns/ Nc	9

Sí; pero no busco	2
Ns/ Nc	9

8. ¿En tu trabajo te realizan aportes jubilatorios? **(espontánea una sola respuesta)**

SI	1
NO	0
Ns/Nc	9

9. ¿Tenés alguna discapacidad física de cualquier tipo? **(espontánea una sola respuesta)**

10. ¿Tenés certificado de discapacidad? **(espontánea una sola respuesta)**

11. ¿Tenés pensión por discapacidad? **(espontánea una sola respuesta)**

	**9**
SI	1
No	0
Ns/Nc	9

→

	**10**
SI	1
No	0
Ns/Nc	9

→

	**11**
SI	1
No	0
Ns/Nc	9

VIVIENDA

12. ¿Cambió tu situación de vivienda (cambiaste de vivienda, de personas con las que vivís o mantenés) o ingresos económicos en los últimos 6 meses? **(espontánea una sola respuesta)**

SI	1
NO	0
Ns/nc	9

Sigue a 21

13. ¿Cuál es tu tipo de vivienda? **(lea las alternativas una sola respuesta)**

Casa	1	Pieza de hotel fam/ pensión	6
Rancho	2	Local no construido p/ habit	7
Casilla	3	Vivienda móvil	8
Departamento	4	Situación de calle	10
Pieza de inquilinato	5	Ns/ Nc	9

14. En tu baño; ¿tenés inodoro con descarga de agua? **(espontánea una sola respuesta)**

SI	1
NO	0
Ns/Nc	9

15. ¿Cuál es el régimen de tenencia de tu vivienda? **(lea las alternativas una sola respuesta)**

Propietario	1	Usurpante (okupas)	4
Inquilino	2	Otros **(No leer)**	5
Ocupante gratuito	3	Ns/ Nc	9

16. Incluyéndote a vos misma; ¿cuántas personas viven en tu hogar actualmente? **(También incluyendo el personal doméstico si lo tiene) (espontánea una sola respuesta)**

Ns/Nc	99

17. ¿Cuál es el ingreso mensual **personal**? **(guiada simple)**

**<2500**	1
**2500 a 5000**	2
**5000 a 10000**	3
**10000 a 20000**	4
**>20000**	5
Ns/ Nc	9999

18. ¿Cuál es el ingreso mensual del grupo familiar o el grupo con el que vivís? **(Una sola respuesta. Todos los ingresos sumados de las personas que viven en un mismo hogar)**

**<2500**	1
**2500 a 5000**	2
**5000 a 10000**	3
**10000 a 20000**	4
**>20000**	5
Ns/ Nc	9999

19. ¿Cuántos niños/as (de 0 a 18 años) conviven con vos? **(Si es ninguno poner 0) (espontánea una sola respuesta)**

Ns/Nc	99

20. ¿Cuántos niños/as (de 0 a 18 años) dependen de vos económicamente? **(Si es ninguno poner 0) (espontánea una sola respuesta)**

Ns/Nc	99

ESTIGMA Y DISCRIMINACION POR IDENTIDAD TRANS EN DIFERENTES AMBITOS

SALUD

Ahora voy a hacerte una serie de preguntas sobre tu experiencia en los servicios de salud en relación a tu identidad trans

21. Por tu identidad de trans ¿en los **últimos 6 meses** viviste experiencias de discriminación en un hospital o centro privado por parte las personas que voy a nombrarte? **(lea las alternativas puede + de 1 respuesta)**

Médicos	1
Personal administrativo	2
Enfermero	3
Otros pacientes	4
Otros profesionales (servicio social, psicólogos)	5
Otra ¿Cuál?	
Ninguna	0
Ns/ Nc	99

22. Por tu identidad trans ¿en **últimos 6 meses** viviste experiencias de discriminación como las que voy a nombrarte? **(lea las alternativas puede + de 1 respuesta)**

Evitaste ir a un centro privado/ hospital	1
No te atendieron en un servicio de salud/ no respetaron tu turno	2
No te llamaron por tu nombre de elección	3
El personal de servicio se burló de vos o te agredió	4
El personal del servicio te trató prejuiciosamente o de forma despectiva	5
El personal de salud hizo insinuaciones sexuales	6
Te internaron junto con varones	7
Preferiste pagar la atención de salud en un centro privado	8
Otra ¿Cuál?	
Ninguna	0
Ns/ Nc	99

EDUCACION

Ahora voy a hacerte una serie de preguntas sobre educación

23. ¿Estás estudiando, retomaste o intentaste retomar los estudios en los últimos 6 meses? **(espontánea una sola respuesta)**

Estoy estudiando desde hace más de 6 meses	1
Retome los estudios en los últimos 6 meses	2
Intenté retomar los estudios en los últimos 6 meses	3
NO	0
Ns/nc	9

Sigue a 26

24. Por tu identidad de trans ¿en **los últimos 6 meses** experiencias de discriminación en un centro educativo por parte de las personas que voy a nombrarte? **(lea las alternativas puede + de 1 respuesta)**

Directores/as	1
Maestros o profesores	2
Personal no docente (preceptor, portero, etc.)	3
Compañeros de estudio	4
Otros profesionales (psicopedagogos, psicólogos)	5
Otra ¿Cuál?	
Ninguna	0
Ns/ Nc	99

25. Por tu identidad trans ¿los últimos 6 meses te ocurrió alguna de las siguientes situaciones que voy a mencionarte? **(lea las alternativas puede + de 1 respuesta)**

Te negaron matricularte o no te dejaron acceder a clases	1
Te sentiste forzada a abandonar tu educación y/o capacitación	2
No aprovechaste una oportunidad educativa y/o capacitación	3
El personal docente se burló/ te agredió	4
No te llamaron por tu nombre de elección	5
Te negaron el acceso a los baños	6
Maltrato de compañeros (bullying)	7
Otras:	

Ninguna de estas	0
Ns/ Nc	99

TRABAJO

Ahora voy a hacerte una serie de preguntas sobre tu trabajo

A LAS QUE REALIZAN TRABAJOS DIFERENTES AL TRABAJO SEXUAL EN LOS ÚLTIMOS 6 MESES

26. Por tu identidad trans ¿en **los últimos 6 meses** sentiste rechazo o viviste experiencias de discriminación en el trabajo por parte de las personas que voy a nombrarte? **(lea las alternativas puede + de 1 respuesta)**

jefes/directivos	1
compañeros de trabajo	2
personal de otras áreas	3
clientes/proveedores	4
Otra ¿Cuál?	
Ninguna	0
Ns/ Nc	99

A LAS QUE REALIZAN TRABAJO SEXUAL EN LOS ÚLTIMOS 6 MESES

27. Por tu identidad trans ¿en **los últimos 6 meses** sentiste rechazo o viviste experiencias de discriminación en el trabajo por parte de las personas que voy a nombrarte? **(lea las alternativas puede + de 1 respuesta)**

Cliente sexual	1
De la persona que te regentea	2
Otras trabajadoras sexuales	3
Vecinos de la zona	4
Dueño/operador del lugar donde trabajás	5
Otra ¿Cuál?	
Ninguna	0
Ns/ Nc	99

A TODAS

28. Por tu identidad trans ¿en los últimos 6 meses te ocurrió alguna de las siguientes situaciones que voy a mencionarte? **(lea las alternativas puede + de 1 respuesta)**

Te negaron un trabajo	1
Tuviste que dejar un trabajo	2
Decidiste no solicitar un empleo/ trabajo o ascenso laboral	3
No te llamaron por tu nombre de elección	4
Te negaron el acceso a los baños	5
Tus compañeros de trabajo se burlaron o te amenazaron	6
Un cliente te amenazó, insultó, humilló o gritó de una manera que te hiciera sentir miedo	7
Un cliente te golpeó, pateó, empujó o lastimó físicamente	8
Un cliente te obligó a tener relaciones sexuales en contra de tu voluntad	9
Otros:	
Ninguna	0
Ns/ Nc	99

VIVIENDA

Ahora voy a hacerte una serie de preguntas sobre el lugar donde vivís

29. **Por tu identidad trans ¿en los últimos 6 meses** te ocurrió alguna de las siguientes situaciones que voy a mencionarte vinculadas a la vivienda? **(lea las alternativas puede + de 1 respuesta)**

Tus vecinos te insultaron **verbalmente**, te acosaron, y/o amenazaron	1
Tus vecinos te acosaron, amenazaron y/o agredieron **físicamente**	2
Los vecinos hicieron una denuncia en tu contra	3
Te negaron un alquiler o la compra de una vivienda	4
Te cobraron más de alquiler	5
Fuiste forzada a cambiar tu lugar de residencia	6
Otros:	
Ninguna	0
Ns/ Nc	99

FUERZAS DE SEGURIDAD

Ahora voy a hacerte una serie de preguntas en relación a las Fuerzas de Seguridad (policía, gendarmería, etc.)

30. **Por tu identidad trans ¿en los últimos 6 meses** vez fuiste detenida por la policía? (espontánea una sola respuesta)

SI	1
NO	0
Ns/nc	9

NO / Ns/nc → **Sigue a 33**

31. Si fuiste detenida ¿cuál fue la razón? **(lea las alternativas puede + de 1 respuesta)**

Averiguación de antecedentes (“portación de cara”)	1
Prostitución (Contravencionales, edictos, “vestirse de mujer”, etc.)	2
Delitos relacionados con drogas	3
Merodeo/ “movimientos sospechosos”	4
Pelea callejera/Agresión en la vía pública	5
Resistencia a la autoridad	6
Homicidio	7
Hurto	8
Por no tener en regla los papeles del auto/moto	9
Tenencia de dinero falso	10
Otro:	
Ninguna	0
Ns/ Nc	99

32. Por tu identidad trans ¿en los últimos 6 meses te ocurrió alguna de las siguientes situaciones que voy a mencionarte**? (lea las alternativas puede + de 1 respuesta)**

Te mantuvieron detenida más tiempo que a otras personas no trans	1
Te privaron de algún derecho (como llamar a un abogado)	2
Te ubicaron en una celda junto con varones	3
Te realizó una requisa un varón	4
Un policía te amenazó, insultó, humilló o gritó de una manera que te hiciera sentir miedo	5
Un policía te golpeó, pateó, empujó o lastimó físicamente	6
Un policía te obligó a tener relaciones sexuales en contra de tu voluntad	7

No te permitieron que te revisara un médico en caso de golpes o heridas	8
Otros:	
Ns/ Nc	99

SOCIAL (VECINOS/FAMILIA)

Ahora voy a hacerte una serie de preguntas sobre tu vida cotidiana y situaciones sociales en relación a tu identidad trans

33. ¿En tu vida cotidiana, **en los últimos 6 meses** con qué frecuencia te sucedieron las siguientes situaciones por ser una mujer trans?

	Nunca	A veces	Normalmente	A menudo	Siempre
33.1 Te tratan con menos cortesía	1	2	3	4	5
33.2 Te tratan con menos respeto	1	2	3	4	5
33.3 Recibís un servicio menor al adecuado	1	2	3	4	5
33.4 La gente actúa como si te tuvieran miedo	1	2	3	4	5
33.5 La gente actúa como si no fueras inteligente	1	2	3	4	5
33.6 La gente actúa como si fueras deshonesta	1	2	3	4	5
33.7 La gente actúa como si fueran mejores que vos	1	2	3	4	5
33.8 Te insultan	1	2	3	4	5
33.9 Te amenazan o acosan verbalmente	1	2	3	4	5
33.10 Te hacen insinuaciones sexuales (en el trasporte público o en la calle)	1	2	3	4	5

34. Por tu identidad trans ¿**en los últimos 6 meses** te ocurrió alguna de las siguientes situaciones que voy a mencionarte? **(lea las alternativas puede + de 1 respuesta)**

	Nunca	~~A~~ veces	Normalmente	A menudo	Siempre
36.11. Fuiste excluida de reuniones o actividades sociales (ej. bodas, funerales, fiestas, clubes)	1	2	3	4	5
36.12. Fuiste excluida de actividades religiosas o de lugares de culto	1	2	3	4	5
36.13. Fuiste excluida de actividades familiares (ej: cocinar, comer juntos, dormir en la misma habitación)?	1	2	3	4	5
36.14. Tus familiares te insultaron **verbalmente**, te acosaron, y/o amenazaron	1	2	3	4	5
36.15. Tus familiares te acosaron, amenazaron y/o agredieron **físicamente**	1	2	3	4	5
36.16. Sufriste violencia psicológica por parte de una pareja sexual (NO CLIENTE)	1	2	3	4	5
36.17. Sufriste violencia física por parte de una pareja sexual (NO CLIENTE)	1	2	3	4	5
36.18. Sufriste violencia sexual por parte de una pareja sexual (NO CLIENTE)	1	2	3	4	5

36.19. Experimentaste rechazo sexual por parte de una potencial pareja como resultado de tu identidad trans	1	2	3	4	5
36.20. Tu pareja o alguno de los miembros de tu grupo familiar experimentó discriminación como resultado de tu identidad trans	1	2	3	4	5

35. Por tu identidad trans ¿en los últimos 6 meses te sentiste alguna de las emociones que voy a mencionarte**? (lea las alternativas puede + de 1 respuesta)**

	Nunca	A veces	Normalmente	A menudo	Siempre
37.6. Te sentiste avergonzada	1	2	3	4	5
37.7. Te sentiste culpable	1	2	3	4	5
37.8. Con baja autoestima	1	2	3	4	5
37.9. Sentís que deberías ser castigada	1	2	3	4	5

36. ¿En los últimos 6 meses tuviste ideas suicidas?

SI	1
NO	0
Ns/Nc	9

37. ¿En los últimos 6 meses intestaste suicidarte? (espontánea una sola respuesta)

SI	1
NO	0
Ns/Nc	9

38. Por tu identidad trans ¿en los últimos 6 meses te ocurrió alguna de las siguientes situaciones que voy a mencionarte? **(lea las alternativas puede + de 1 respuesta)**

	Nunca	A veces	Normalmente	A menudo	Siempre
40.4. Preferiste no asistir a reuniones sociales	1	2	3	4	5
40.5. Te aislaste de tu familia	1	2	3	4	5
40.6. Te aislaste de tus amigos	1	2	3	4	5
38.4. Dejaste de tomar transporte público	1	2	3	4	5
38.5. No volviste a tu ciudad/pueblo de origen	1	2	3	4	5

39. Por tu identidad trans ¿en los últimos 6 meses tuviste miedo a que te ocurrieran alguna siguientes situaciones que voy a mencionarte (ocurrieran o no)? **(lea las alternativas puede + de 1 respuesta)**

	Nunca	A veces	Normalmente	A menudo	Siempre
41.5. Temor a ser blanco de murmuraciones	1	2	3	4	5
41.6. Temor a ser insultada, acosada y/o amenazada verbalmente	1	2	3	4	5
41.7. Temor a ser agredida físicamente	1	2	3	4	5
41.8. Temor a que alguien no quiera entablar una relación sexual íntima con	1	2	3	4	5

vos debido a su identidad trans					

TRAMITES SISTEMA PÚBLICO

40. Por tu identidad trans ¿en los últimos 6 meses te ocurrió alguna siguientes situaciones que voy a mencionarte? **(lea las alternativas puede + de 1 respuesta)**

Te negaron realizar un trámite en una institución pública (no de salud ni educación, ej. ANSES, AFIP, registro civil, etc.)	1
Decidiste no realizar un trámite en una institución pública	2
No te llamaron por tu nombre de elección en una institución pública	3
El personal de la institución pública se burló de vos o insultó	4
Otras personas que estaban en la institución pública se burlaron o te insultaron	5
Ninguna	0
Ns/ Nc	99

DISCRIMINACION POR VIH

Ahora voy a hacerte una serie de preguntas sobre tus experiencias en los servicios de salud en relación a tu diagnóstico de VIH

41. Por tener VIH, ¿alguna vez viviste experiencias de discriminación en un hospital o centro privado por parte de las personas que voy a nombrarte? **(lea las alternativas puede + de 1 respuesta)**

Médico	1
Personal administrativo	2
Enfermeros	3
Otros pacientes	4
Otros profesionales (servicio social, psicólogos)	5
Otra persona trans	6
Otra ¿Cuál?	7
Ninguna	0
Ns/ Nc	99

CONSUMO DE SUSTANCIAS

Ahora voy a hacerte algunas preguntas respecto al consumo de bebidas alcohólicas. Como parte de nuestra evaluación, es importante conocer los estilos de vida que pueden afectar tu salud. Por lo tanto, te pedimos que completes con total honestidad este cuestionario de preguntas sobre el consumo de alcohol. Al momento de responder considera que "una bebida que contiene alcohol" equivale a: Una lata o botella pequeña de cerveza (330 ml) o una copa de vino tinto o blanco, o un vaso con 40 ml de pisco, vodka, tequila, whiskey, ron (u otros tipos de tragos fuertes con alcohol).

42. Desde la última visita (__/__/__), ¿cada cuánto tomaste una bebida que contiene alcohol? (**espontánea una sola respuesta)**

4 veces o más a la semana	4
2-3 veces por semana	3
2-4 veces al mes	2
Una vez al mes	1
Nunca	0
Ns/ Nc	99

43. Desde la última visita (__/__/__), ¿cuántos tragos tomaste en un día normal cuando estás tomando? (**espontánea una sola respuesta)**

10 o más	4
7 o 9	3
5 o 6	2
3 o 4	1
1 o 2	0
Ns/ Nc	99

44. Desde la última visita (__/__/__), ¿con qué frecuencia tomaste seis a más tragos en una sola salida? (**espontánea una sola respuesta)**

Casi a diario o todos los días	4
Cada semana	3
Cada mes	2
Menos de una vez al mes	1
Nunca	0
Ns/ Nc	99

Ahora vamos a preguntarte, sobre tu consumo de alcohol en los últimos 6 meses

45. ¿Con qué frecuencia durante **los últimos 6 meses** no pudiste dejar de tomar una vez que empezaste a hacerlo? (**espontánea una sola respuesta)**

Casi a diario o todos los días	4
Cada semana	3
Cada mes	2
Menos de una vez al mes	1
Nunca	0
Ns/ Nc	99

46. ¿Con qué frecuencia durante **los últimos 6 meses**, dejaste de hacer algo que normalmente esperarías hacer a causa de la bebida? (**espontánea una sola respuesta)**

Casi a diario o todos los días	4
Cada semana	3
Cada mes	2
Menos de una vez al mes	1
Nunca	0
Ns/ Nc	99

47. ¿Con qué frecuencia durante **los últimos 6 meses**, necesitaste de un trago en la mañana para reponerte después de una buena borrachera? (**espontánea una sola respuesta)**

Casi a diario o todos los días	4
Cada semana	3
Cada mes	2
Menos de una vez al mes	1
Nunca	0
Ns/ Nc	99

48. ¿Con qué frecuencia durante **los últimos 6 meses** te sentiste culpable o con remordimientos después de tomar? (**espontánea una sola respuesta)**

Casi a diario o todos los días	4
Cada semana	3
Cada mes	2
Menos de una vez al mes	1
Nunca	0
Ns/ Nc	99

49. ¿Con qué frecuencia durante **los últimos 6 meses** fuiste incapaz de recordar lo que pasó la noche anterior a causa de la bebida? (**espontánea una sola respuesta)**

Casi a diario o todos los días	4
Cada semana	3
Cada mes	2
Menos de una vez al mes	1
Nunca	0
Ns/ Nc	99

50. ¿Vos o alguien resultó herido o maltratado como resultado de que estabas tomando alcohol? ¿en **los últimos 6 meses**? (**espontánea una sola respuesta)**

Sí, pero no en los últimos 6 meses	4
Sí, durante los últimos 6 meses	2
No	0
Ns/ Nc	99

51. ¿Algún pariente, amigo, doctor o trabajador de salud ha estado preocupado por lo que tomás o te ha sugerido dejar de tomar? ¿en **los últimos 6 meses**? (**espontánea una sola respuesta)**

Sí, pero no en los últimos 6 meses	4
Sí, durante los últimos 6 meses	2
No	0
Ns/ Nc	99

52. ¿Algún doctor o trabajador de salud no te atendió porque habías tomando alcohol? ¿en **los últimos 6 meses**? (**espontánea una sola respuesta)**

Sí, pero no en el último año	4
Sí, durante el último año	2
No	0
Ns/ Nc	99

53. ¿Alguna vez dejaste de ir a consultar a un hospital/centro de salud o retirar medicación porque habías tomando alcohol? ¿en **los últimos 6 meses**? (**espontánea una sola respuesta)**

Sí, pero no en el último año	4
Sí, durante el último año	2
No	0
Ns/ Nc	99

54. En **los últimos 6 meses**, con quien tomaste alcohol: **(espontánea más de una respuesta)**

Sola	0
Clientes sexuales	1
Pareja	2

Compañeras trans	3
Discos/Boliches	4
Otros:	
Ns/ Nc	99

Las siguientes preguntas indagan sobre tu posible uso de drogas (excluyendo alcohol y tabaco) durante los últimos 6 meses. Por favor contestá con honestidad ¨SÍ¨ o ¨NO¨. Cuando se usa la expresión ¨abuso de drogas¨, nos referimos al uso de drogas prescritas por el médico, o las que se compran sin receta, o drogas recreativas. Algunos ejemplos de drogas incluyen pero no se limitan a: cannabis (marihuana.), drogas tranquilizantes (como Valium), barbitúricos, cocaína, estimulantes, alucinógenos (como LSD) o narcóticos (como heroína). Por favor no te olvides que estas preguntas no incluyen el uso de alcohol o tabaco.

55. Desde la última visita (__/__/__), ¿usaste otras drogas diferentes a las requeridas por razones médicas? (**espontánea una sola respuesta)**

SI	1
NO	0
Ns/Nc	9

} **Pasa a Comportamiento sexual**

56. Desde la última visita (__/__/__), ¿consumiste alguna de estas sustancias? (**espontánea una sola respuesta)**

Marihuana	1
Cocaína	2
Crack	3
Anfetaminas/cristal	4
Heroína	5
Pasta base/PACO	6
Clonazepam(no recetado)	7
Ketamina	8
Otros ¿Cuál?	
Nunca	0
Ns/ Nc	99

57. Desde la última visita (__/__/__), ¿Cuantos días consumiste drogas? (**espontánea una sola respuesta)**

7 días o más	2
Menos de 7 días	1
Nunca	0
Ns/ Nc	99

→ **Si es menor a 7 pasa a Comportamiento sexual**

58. En los últimos 6 meses ¿Con qué frecuencia consumiste? (**espontánea una sola respuesta)**

Casi a diario o todos los días	4
Cada semana	3
Cada mes	2
Menos de una vez al mes	1
Nunca	0
Ns/ Nc	99

59. En los últimos 6 meses ¿Cuantos días consumiste más de lo que tenías intención de consumir? (**espontánea una sola respuesta)**

2 o más días	2
Menos de 2 días	1
Nunca	0
Ns/ Nc	99

Si es menor a 2 pasa a Comportamiento sexual

60. En los últimos 6 meses, ¿abusaste de más de una droga a la vez? (**espontánea una sola respuesta)**

SI	1
NO	0
Ns/Nc	9

61. En los últimos 6 meses, ¿pudiste dejar de usar las drogas cuando querías? (**espontánea una sola respuesta)**

SI	1
NO	0
Ns/Nc	9

62. En los últimos 6 meses, ¿tuviste una pérdida de conocimiento o una escena retrospectiva (o sea a retrocedido a vivencias del pasado) por causa de las drogas? (**espontánea una sola respuesta)**

SI	1
NO	0
Ns/Nc	9

63. En los últimos 6 meses, ¿te sentiste a veces mal o culpable sobre tu uso de drogas? (**espontánea una sola respuesta)**

SI	1
NO	0
Ns/Nc	9

64. En los últimos 6 meses, ¿tu pareja (o padres) se quejaron de tu uso de drogas? (**espontánea una sola respuesta)**

SI	1
NO	0
No tengo familia ni pareja	8
Ns/Nc	9

65. En los últimos 6 meses, ¿descuidaste a tu familia/pareja por usar drogas? (**espontánea una sola respuesta)**

SI	1
NO	0
No tengo familia ni pareja	8
Ns/Nc	9

66. En los últimos 6 meses, ¿te involucraste en actividades ilegales para conseguir las drogas? (**espontánea una sola respuesta)**

SI	1
NO	0

Ns/Nc	9

67. En los últimos 6 meses, ¿tuviste síndrome de abstinencia (te sentiste enferma) cuando dejaste de tomar drogas? (**espontánea una sola respuesta)**

SI	1
NO	0
Ns/Nc	9

68. En los últimos 6 meses, ¿tuviste problemas médicos por el uso de drogas (por ejemplo, una pérdida de memoria, hepatitis, convulsiones, pérdida de sangre, etc.)? (**Espontánea una sola respuesta)**

SI	1
NO	0
Ns/Nc	9

69. **En los últimos 6 meses**, con quien consumiste drogas:

Sola	0
Clientes sexuales	1
Pareja	2
Compañeras trans	3
Discos/Boliches	4
Otros:	
Ns/ Nc	99

COMPORTAMIENTO SEXUAL

Para esta parte de la encuesta, en la que vamos a hablar de tu vida sexual, la palabra "sexo" significa haber tenido contacto genital con otra persona que puede o no haberte dado placer o a tu compañero/a (esto incluye relaciones sexuales anales, orales o vaginales). Sexo NO incluye actividades tales como "sexo por teléfono" o besos, actividades que no involucran contacto genital. Utilizaremos la palabra “parejas sexuales” para referirnos a todas las personas con las que hayas tenido relaciones sexuales (parejas estables, ocasionales o clientes)

Todas las preguntas a continuación hacen referencia a las parejas sexuales en el **último mes**, esto significa desde **__/__/__ (fecha). Si no te acordás el número exacto de parejas, por favor proporcióname el número que más se acerque según tus cálculos**

70. ¿Cuántas veces has tenido una pareja diferente en el último mes? (hombres, mujeres, trans e incluye sexo oral)

Ninguna	1 al mes	2 al mes	1 cada semana	2 a la semana	3 a la semana	1 cada 2 días	1 al día	2 al día	3 al día	4 al día
0	1	2	4	8	12	15	30	60	90	120

5 al día	6 al día	7 al día	8 al día	9 al día	10 al día	11 al día	12 Al día	13 Al día	15 Al día	Otro calculo
150	180	210	240	270	300	330	360	390	450	

71. ¿Cuántas de estas parejas eran clientes sexuales? (hombres, mujeres, trans e incluye sexo oral) **(espontánea una sola respuesta)**

Ns/Nc	99

72. ¿Con cuántas parejas has consumido alcohol/drogas antes o durante una relación sexual? (hombres, mujeres, trans e incluye sexo oral) **(espontánea una sola respuesta)**

Ns/Nc	99

73. ¿Con cuántos hombres (clientes o no) has tenido sexo en el último mes? **(espontánea una sola respuesta)**

Ns/Nc	99

Si no tuvo relaciones con hombres en el último mes pase a 83

74. ¿Con cuántos hombres has consumido alcohol/drogas antes o durante una relación sexual? **(espontánea una sola respuesta)**

Ns/Nc	99

75. ¿Cuántos hombres han eyaculado en tu boca durante el sexo oral en el último mes? **(espontánea una sola respuesta)**

Ns/Nc	99

76. ¿Con cuántos hombres has tenido sexo anal en el último mes? (relaciones donde tu rol fue activo y/o pasivo) **(espontánea una sola respuesta)**

Ns/Nc	99

77. ¿Con cuántos hombres has tenido sexo anal penetrativo (tu pene en su ano, activo; “se la metiste”) en el último mes? **(espontánea una sola respuesta)**

Ns/Nc	99

78. De estos hombres, ¿con cuántos has tenido sexo anal penetrativo (activo) sin preservativo? **(espontánea una sola respuesta)**

Ns/Nc	99

79. De estos hombres con los que tuviste sexo penetrativo (activo), ¿cuántos fueron : **(espontánea una sola respuesta)**

VIH+		VIH-		No se	
Ns/Nc	99	Ns/Nc	99	Ns/Nc	99

80. ¿Con cuántos hombres has tenido sexo anal receptivo (su pene en tu ano; pasivo; “te la metieron”) en el último mes? **(espontánea una sola respuesta)**

Ns/Nc	99

81. De estos hombres, ¿con cuántos has tenido sexo anal receptivo (pasivo) sin preservativo? **(espontánea una sola respuesta)**

Ns/Nc	99

82. De estos hombres con los que tuviste sexo receptivo (pasivo), ¿cuántos fueron : **(espontánea una sola respuesta)**

VIH+		VIH-		No se	
Ns/Nc	99	Ns/Nc	99	Ns/Nc	99

83. ¿Con cuántas mujeres (clientes o no) has tenido sexo en el último mes? **(espontánea una sola respuesta)**

Ns/Nc	99

Si no tuvo relaciones con mujeres en el último mes pase a 87

84. ¿Con cuántas mujeres has tenido sexo vaginal o anal en el último mes? **(espontánea una sola respuesta)**

Ns/Nc	99

85. De estas mujeres, ¿con cuantas has tenido sexo vaginal o anal sin preservativo? **(espontánea una sola respuesta)**

Ns/Nc	99

86. De estas mujeres, cuántas fueron: **(espontánea una sola respuesta)**

VIH+		VIH-		No se	
Ns/Nc	99	Ns/Nc	99	Ns/Nc	99

87. ¿Con cuántas **personas trans** (clientes o no) has tenido sexo en el último mes? **(espontánea una sola respuesta)**

Ns/Nc	99

Si no tuvo relaciones con personas trans en el último mes pase a 91

88. ¿Con cuántas personas trans has tenido sexo vaginal o anal en el último mes? **(espontánea una sola respuesta)**

Ns/Nc	99

89. De estas personas trans, ¿con cuantas has tenido sexo vaginal o anal sin preservativo? **(espontánea una sola respuesta)**

Ns/Nc	99

90. De estas personas trans, cuántas fueron: **(espontánea una sola respuesta)**

**VIH+**		**VIH-**		**No se**	
Ns/Nc	99	Ns/Nc	99	Ns/Nc	99

91. ¿Actualmente tenés una pareja sexual estable (persona con quien tenés relaciones sexuales frecuentemente y una relación de compromiso mutuo)? **(espontánea una sola respuesta)**

	**91**
SI	1
NO	0
Ns/ Nc	9

Pasar a 95

92. ¿Cuánto tiempo (en meses) hace que tenés esta pareja? **(espontánea una sola respuesta)**

	**ESCRIBIR un Nº**
Ns/ Nc	99

93. ¿Cuál es el género de tu actual pareja sexual estable? **(lea las alternativas una sola respuesta)**

Mujer	1
Varón	2
Trans	3
Ns/ Nc	9

94. Al momento de tener relaciones sexuales con tu pareja, ¿con qué frecuencia utilizan preservativos? **(lea las alternativas una sola respuesta)**

Siempre	3
La mayoría de veces	2
La minoría de veces	1

Nunca	0
Ns/ Nc	9

95. ¿Utilizaste preservativo en tu **última relación sexual**? **(espontánea una sola respuesta)**

NO	0
SI	1
Ns/Nc	9

96. ¿Cuáles fueron las razones por las que no usaste preservativo en la última relación sexual? **(espontánea puede + de 1 respuesta)**

Porque mi pareja/cliente no quiso usarlo	1
Porque yo no quise usarlo	2
Porque le resta sensibilidad a la relación	3
Porque en ese momento no tenía	4
Porque corta el momento de la relación	5
Me encontraba bajo la influencia del alcohol	6
Me encontraba bajo la influencia de alguna droga	7
Porque tengo pareja estable	8
Porque confío en mi pareja	9
Porque mi pareja y yo tenemos VIH	10
Porque tomo medicación para el VIH y estoy indectable	11
Porque usamos otro tipo de protección (ej. Banda de látex)	12
Porque el cliente me paga más si no usamos preservativo	13
Porque me incomoda	14
Por descuido	15
Otros :	
Ns/ Nc	99

5) CUESTIONARIO DE ADHERENCIA (ACTG MODIFICADO)

Participante # _____________ **Iniciales** ________ **Visita** ________ **Fecha de Visita** __ __ / __ __ __ / __ __ __ __

Este cuestionario contiene preguntas acerca de los medicamentos que usted está tomando. A muchas personas les resulta difícil recordar tomar siempre sus pastillas. Por ejemplo:

- Algunas personas están muy ocupadas y olvidan llevar sus pastillas consigo
- Algunas personas les resultan difícil tomar sus pastillas o seguir las instrucciones del médico, como por ejemplo tomar el dolutegravir y tenofovir+emtricitabina juntas y con algún alimento
- Algunas personas deciden dejar de tomar los medicamentos por diferentes razones

Necesitamos entender realmente como toma el tratamiento. Por favor cuéntenos con sinceridad lo que está haciendo. Necesitamos que nos cuente lo que realmente está sucediendo y no lo que usted crea que "queremos oír". No tenga miedo de decir la verdad.

1) ¿Dejo de tomar alguna medicación?

	**MARQUE CON UNA CRUZ CUANDO DEJÓ DE TOMAR ALGUNA DOSIS. PONGA CERO (O) SI NO OMITIÓ NINGUNA**			
**MEDICAMENTO**	**AYER**	**ANTES DE AYER**	**HACE 3 DÍAS**	**HACE 4 DÍAS**
DOLUTEGRAVIR				
TENOFOVIR+EMTRICITABINA				

2) Estas pastillas deben tomarse 1 vez al día aproximadamente al mismo horario ¿Qué tan bien cumplió este horario?.

□ 0- Nunca
□ 1- Parte del tiempo
□ 2- La mitad del tiempo
□ 3- La mayor parte del tiempo
□ 4-Todo el tiempo

3) Estas pastillas deben tomarse juntas y con alguna comida ¿Qué tan bien cumplió estas instrucciones?.

□ 0- Nunca
□ 1- Parte del tiempo
□ 2- La mitad del tiempo
□ 3- La mayor parte del tiempo
□ 4-Todo el tiempo

4) Algunas personas no toman las pastillas durante el fin de semana. ¿Dejó de tomar algún medicamento el fin de semana pasado (el último sábado o domingo)?

□ 1: SI □ 2: NO

5) ¿Cuándo fue la última vez que usted no tomó algún medicamento de los indicados?:

□ 5. En la última semana
□ 4. Hace 1- 2 semanas
□ 3. Hace 2-4 semanas
□ 2. Hace 1-3 meses

□ 1. Hace más de 3 meses
□ 0. Nunca tomé

SI USTED NO DEJO DE TOMAR NINGUNA DOSIS DESDE LA ULTIMA VISITA PARE AQUÍ

6) Las personas pueden dejar de tomar los medicamentos del estudio por varias razones. Esta es una lista de las posibles razones por las cuales usted puede haber dejado de tomar los medicamentos. ¿Con qué frecuencia dejo de tomar los medicamentos del estudio debido a:

**Por favor marque una casilla en cada pregunta.**	**Nunca**	**Raramente**	**Algunas veces**	**Con frecuencia**
1) Estaba lejos de la casa?	□ 0	□ 1	□ 2	□ 3
2) Estaba ocupada con otras cosas?	□ 0	□ 1	□ 2	□ 3
3) Simplemente se le olvidó?	□ 0	□ 1	□ 2	□ 3
4) Tenía que tomar muchas pastillas?	□ 0	□ 1	□ 2	□ 3
5) Quería evitar los efectos secundarios?	□ 0	□ 1	□ 2	□ 3
6) No quería que otros la vieran tomar medicación?	□ 0	□ 1	□ 2	□ 3
7) Tuvo un cambio en su rutina diaria?	□ 0	□ 1	□ 2	□ 3
8) Pensó que el medicamento era tóxico?	□ 0	□ 1	□ 2	□ 3
9) Se durmió antes de tomarla?	□ 0	□ 1	□ 2	□ 3
10) Se sintió enferma?	□ 0	□ 1	□ 2	□ 3
11) Se sintió deprimida o agotada?	□ 0	□ 1	□ 2	□ 3
12) Tuvo problemas para tomar las pastillas a determinadas horas (con comidas)?	□ 0	□ 1	□ 2	□ 3

13) Se le acabaron las pastillas? ◻ 0 ◻ 1 ◻ 2 ◻ 3

14) Se sintió bien? ◻ 0 ◻ 1 ◻ 2 ◻ 3

Escala Visual Analógica

Instrucciones: Coloque una **"X"** en el punto que mejor describa la proporción de los antirretrovirales que ha tomado en las últimas cuatro semanas. Cero significaría que no ha tomado nada, 50 sería la mitad de lo que tomó, 100 indicaría que ha tomado todas las dosis de los antirretrovirales en las últimas 4 semanas.

A. Dolutegravir

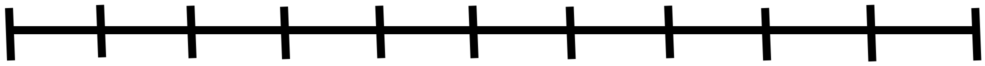

0 10 20 30 40 50 60 70 80 90 100

B. Tenofovir+emtricitabina

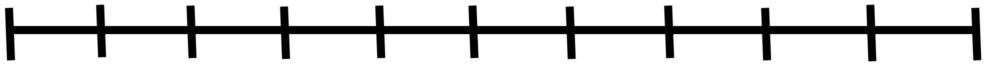

0 10 20 30 40 50 60 70 80 90 100

6) CUESTIONARIO DE CALIDAD DE VIDA (Well-being Index)

Participante # ___________________ **Iniciales** ________ **Visita** ________ **Fecha de Visita** __ __ / __ __ __ / __ __ __ __

Las preguntas son acerca de cuán satisfecha te sentís sobre una escala del cero al diez. Para responder, hacé un círculo en la opción elegida.

Vida en general

1. Pensando acerca de tu propia vida y tus circunstancias personales, ¿cuán satisfecha te sentís con tu vida en general?	0	1	2	3	4	5	6	7	8	9	10
2. ¿Cuán satisfecha te sentís con tu salud?	0	1	2	3	4	5	6	7	8	9	10
3. ¿Cuán satisfecha te sentís con tus logros?	0	1	2	3	4	5	6	7	8	9	10
4. ¿Cuán satisfecha te sentís con tus relaciones personales?	0	1	2	3	4	5	6	7	8	9	10
5. ¿Cuán segura te sentís?	0	1	2	3	4	5	6	7	8	9	10
6. ¿Cuán satisfecha te sentís de ser parte de esta comunidad?	0	1	2	3	4	5	6	7	8	9	10
7. ¿Cuán satisfecha te sentís acerca de tu seguridad futura?	0	1	2	3	4	5	6	7	8	9	10
8. ¿Cuán satisfecha te sentís con tus creencias espirituales y religiosas?	0	1	2	3	4	5	6	7	8	9	10

Eventos en su vida

¿Te sucedió algo últimamente que te hizo sentir más feliz o más triste?	Si, más feliz<br>1	Si, más triste<br>2	No<br>0	Ns/Nc<br>9

**Si respondió afirmativamente**,	Muy débil								Muy fuerte
¿Cuán fuerte definirías esta influencia en tu vida?	1	2	3	4	5	6	7	8	9	10

7) CUESTIONARIO DE APOYO SOCIAL

Participante # __________________ **Iniciales** ________ **Visita** ________ **Fecha de Visita** __ __ / __ __ __ / __ __ __ __

Bockting et al. (2013)

Apoyo familiar

	Nada de apoyo						Mucho apoyo
¿Cómo valorarías el apoyo de tu familia de origen (padres y/o los hermanos) en relación a tu identidad de género?	1	2	3	4	5	6	7

**Ayuda entre pares**	Nada						Todo el tiempo
¿Cuánto tiempo pasas con otras personas trans?	1	2	3	4	5	6	7

	Nunca						Siempre
¿Cuántas veces te sentiste como la única persona trans en el área donde vivís?	1	2	3	4	5	6	7

¿En la actualidad sos miembro de algún grupo de apoyo y/o red de personas trans?	No<br>0	Si<br>1	¿Cuál?

Cuestionario de apoyo social (Duke UNC)

En la siguiente lista se muestran algunas cosas que otras personas hacen por nosotros o nos proporcionan en general. Elija para cada una la respuesta que mejor refleje su situación.

	Mucho menos de lo que deseo	Menos de lo que deseo	Ni mucho ni poco	Casi como deseo	Tanto como deseo
1. Recibo visitas de mis amigos y familiares	1	2	3	4	5
2. Recibo ayuda en asuntos relacionados con mi casa	1	2	3	4	5
3. Recibo elogios y reconocimiento cuando hago bien mi trabajo	1	2	3	4	5
4. Cuento con personas que se preocupan de lo que me sucede	1	2	3	4	5
5. Recibo amor y afecto	1	2	3	4	5
6. Tengo la posibilidad de hablar con alguien de mis problemas en el trabajo o en mi casa	1	2	3	4	5
7. Tengo la posibilidad de hablar con alguien de mis problemas personales y familiares	1	2	3	4	5

8. Tengo la posibilidad de hablar con alguien de mis problemas económicos	1	2	3	4	5
9. Recibo invitaciones para distraerme y salir con otras personas	1	2	3	4	5
10. Recibo consejos útiles cuando me ocurre algún acontecimiento importante en mi vida	1	2	3	4	5
11. Recibo ayuda cuando estoy enferma en la cama	1	2	3	4	5

8) CUESTIONARIO SOBRE DEPRESIÓN E IDEACIÓN SUICIDA

Participante # _____________ **Iniciales** ________ **Visita** ________ **Fecha de Visita** __ __ / __ __ __ / __ __ __ __

Center for Epidemiological Studies – Depression scale (CES-D)+ 4-item suicidal-ideation screener

A continuación hay una lista de como podés haberte sentido o comportado. Por favor, decime con qué frecuencia te sentiste **así durante la semana pasada**

	Rara vez o ninguna de las veces (menos de 1 día)	Algunas veces o por poco tiempo (1-2 días)	Ocasionalmente o una moderada cantidad de tiempo (3-4 días)	La mayoría o todo el tiempo (5-7 días)
1. Me molestaron cosas que usualmente no me molestan	1	2	3	4
2. No me sentía con ganas de comer; tenía poco apetito.	1	2	3	4
3. Sentía que no podía quitarme de encima la tristeza aún con la ayuda de mi familia o amigos.	1	2	3	4
4. Sentía que yo era tan buena como cualquier persona.	1	2	3	4
5. Tenía dificultad en concentrarme en lo que estaba haciendo.	1	2	3	4
6. Me sentía deprimida.	1	2	3	4
7. Sentía que todo lo que hacía me costaba esfuerzo.	1	2	3	4
8. Me sentía optimista sobre el futuro	1	2	3	4
9. Pensé que mi vida había sido un fracaso	1	2	3	4
10. Me sentía con miedo	1	2	3	4
11. Tenía problemas para dormir.	1	2	3	4
12. Estaba contenta.	1	2	3	4
13. Hablé menos de lo usual.	1	2	3	4
14. Me sentí sola.	1	2	3	4
15. La gente no era amigable.	1	2	3	4
16. Disfruté de la vida.	1	2	3	4
17. Pasé ratos llorando.	1	2	3	4
18. Me sentí triste.	1	2	3	4
19. Sentía que no le caía bien a la gente.	1	2	3	4
20. No tenía ganas de hacer nada	1	2	3	4
21. He tenido ideas relacionadas a morir.	1	2	3	4
22. He sentido que mi familia y amigos estarían mejor si yo muriera.	1	2	3	4
23. He pensado acerca de matarme.	1	2	3	4
24. Pienso que podría matarme si supiera como hacerlo.	1	2	3	4

9) CUESTIONARIO SOBRE ANSIEDAD: Inventario de ansiedad-rasgo (STAI)

Participante # ____________ **Iniciales** ________ **Visita** ________ **Fecha de Visita** __ __ / __ __ __ / __ __ __ __

Abajo aparecen algunas expresiones que la gente usa para describirse a sí misma. Leé cada frase y marcá con una cruz el casillero que indique como te sentís GENERALMENTE. No hay respuestas buenas o malas. No utilices demasiado tiempo pare responder cada frase, pero tratá de elegir la respuesta que mejor describa tus sentimientos GENERALMENTE.

	Nada	Un poco	Bastante	Mucho
1) Me siento bien	1	2	3	4
2) Me siento nerviosa	1	2	3	4
3) Me siento “a gusto” conmigo misma	1	2	3	4
4) Quisiera ser tan feliz como otros parecen serlo	1	2	3	4
5) Siento que fallo	1	2	3	4
6) Me siento descansada	1	2	3	4
7) Soy una persona tranquila, serena y calmada	1	2	3	4
8) Siento que las dificultades se me amontonan y no las puedo superar	1	2	3	4
9) Me preocupo demasiado por cosas sin importancia	1	2	3	4
10) Soy feliz	1	2	3	4
11) Tengo malos pensamientos	1	2	3	4
12) Me falta confianza en mí misma	1	2	3	4
13) Me siento segura	1	2	3	4
14) Puedo decidirme rápidamente	1	2	3	4
15) Me siento “fuera de lugar”	1	2	3	4
16) Me siento satisfecha	1	2	3	4
17) Algunas ideas poco importantes ocupan mi cabeza y me molestan	1	2	3	4
18) Los desengaños me afectan tanto que no me los puedo sacar de la cabeza	1	2	3	4

19) Soy una persona estable	1	2	3	4
20) Cuando pienso las cosas que tengo entre manos me pongo nerviosa y tensa	1	2	3	4

10) ESCALA DE ESTIGMA EN VIH: Berger HIV Stigma Scale

Participante # ___________________ **Iniciales** ________ **Visita** ________ **Fecha de Visita** __ __ **/** __ __ __ **/** __ __ __ __

Este estudio pregunta sobre algunos aspectos sociales y emocionales sobre tener VIH. No hay respuestas correctas o incorrectas. Este grupo de preguntas habla sobre tus experiencias, sentimientos y opiniones acerca de lo que los pacientes con el VIH sienten y de cómo son tratados. Por favor, contestá lo mejor que puedas cada pregunta.

	Muy en Desacuerdo	Desacuerdo	De Acuerdo	Muy de Acuerdo
1.En muchos ámbitos de mi vida, nadie sabe que tengo VIH	1	2	3	4
2.Me siento culpable por tener VIH	1	2	3	4
3.La actitud de la gente acerca del VIH me hace sentir peor sobre mí misma	1	2	3	4
4.Contarle a alguien que tengo VIH es arriesgado	1	2	3	4
5.Personas con VIH pierden sus empleos cuando sus empleadores se enteran	1	2	3	4
6.Yo hago muchos esfuerzos para mantener en secreto que tengo VIH	1	2	3	4
7.Siento que no soy tan buena persona por tener VIH	1	2	3	4
8.Nunca me siento avergonzada de tener VIH	1	2	3	4
9.Las personas con VIH son tratadas como marginadas	1	2	3	4
10.Mucha gente piensa que una persona con VIH es sucia	1	2	3	4
11.Es más fácil evitar nuevas amistades que preocuparse por decirle a alguien que tengo VIH	1	2	3	4
12.El VIH me hace sentir sucia	1	2	3	4
13.Desde que supe que tengo VIH me siento apartada del resto del mundo	1	2	3	4
14.La mayoría de la gente piensa que una persona con VIH es repugnante	1	2	3	4
15.Tener VIH me hace sentir que soy una mala persona	1	2	3	4
16.Muchas personas con VIH son rechazadas cuando otras personas se enteran	1	2	3	4
17.Soy muy cuidadosa sobre a quién le digo que tengo VIH	1	2	3	4
18.Algunas personas que saben que tengo VIH se han distanciado	1	2	3	4
19.Desde que supe que tengo VIH me preocupa que me discriminen	1	2	3	4
20.La mayoría de las personas se sienten incómodas alrededor de alguien con VIH	1	2	3	4
21.Nunca siento la necesidad de esconder el hecho de que tengo VIH	1	2	3	4
22.Me preocupa que me juzguen al saber que	1	2	3	4

tengo VIH				
23.Me repugna saber que tengo VIH en mi cuerpo	1	2	3	4

Muchas de las preguntas en esta sección asumen que les dijiste a otras personas que tiene VIH, o que otras personas lo saben. Estas preguntas no son necesariamente verdaderas. Si las preguntas se refieren a algo que no te ha sucedido, imagínate en esa situación. Luego marcá con un círculo tu respuesta.

	**Muy en Desacuerdo**	**Desacuerdo**	**De Acuerdo**	**Muy de Acuerdo**
24. Me duele la manera en que la gente ha reaccionado al enterarse que tengo VIH	1	2	3	4
25.Me preocupa que las persona que saben que tengo VIH se lo cuenten a otros	1	2	3	4
26.Me arrepiento de haberle contado a algunas personas que tengo VIH	1	2	3	4
27.Como norma, decirle a otras personas que tengo VIH ha sido un error	1	2	3	4
28.Algunas personas evitan tocarme desde que saben que tengo VIH	1	2	3	4
29.Gente a la que quiero ha dejado de llamarme desde que se enteraron que tengo VIH	1	2	3	4
30.La gente me ha dicho que merezco tener VIH por la clase de vida que he llevado	1	2	3	4
31.Algunas persona cercanas a mí tienen miedo que otros los rechacen si se sabe que tengo VIH	1	2	3	4
32.La gente no me quiere alrededor de sus niños cuando se enteran que tengo VIH	1	2	3	4
33.La gente se ha alejado físicamente de mí cuando se enteran que tengo VIH	1	2	3	4
34.Algunas personas actúan como si fuese mi culpa tener VIH	1	2	3	4
35.He dejado de socializar con algunas personas debido a sus reacciones al saber que tengo VIH	1	2	3	4
36.He perdido amigos por haberles dicho que tengo VIH	1	2	3	4
37.Le he pedido a gente que conozco que no diga que tengo VIH	1	2	3	4
38.La gente que sabe que tengo VIH tienden a ignorar las cosas positivas en mí	1	2	3	4
39.La gente parece asustarse en el momento en que conocen que tengo VIH	1	2	3	4
40.Cuando la gente sabe que tenés VIH, buscan defectos en tu persona	1	2	3	4

11) CUESTIONARIO SOBRE PERSONALIDAD:
Inventario de Personalidad para el DSM-5 (PID-5 Abreviado) - Adulto

Participante # ___________ **Iniciales** ________ **Visita** ________ **Fecha de Visita** __ __ / __ __ __ / __ __ __ __

Instrucciones. Esta es una lista de cosas que diferentes personas podrían decir sobre sí mismas. Estamos interesados en cómo se describiría usted mismo. No hay respuestas "correctas" o "incorrectas", de manera que usted puede describirse a sí mismo lo más honestamente posible. Sus respuestas serán tratadas en forma confidencial. Quisiéramos que usted se tome su tiempo y lea cada frase cuidadosamente, seleccionando la respuesta que mejor lo describa.

	Muy Falso o A menudo Falso	A veces o Algo Falso	A veces o Algo Verdadero	Muy Cierto o A menudo	Verdadero
1 Creo que la gente me describiría como imprudente	0	1	2	3	4
2 Siento como si actuara totalmente por impulso.	0	1	2	3	4
3 A pesar de saber que tomo decisiones precipitadas, no puedo dejar de hacerlo	0	1	2	3	4
4 Nada parece interesarme mucho	0	1	2	3	4
5 Los demás me ven como irresponsable.	0	1	2	3	4
6 No soy bueno/a para planificar	0	1	2	3	4
7 A menudo mis pensamientos no tienen sentido para los demás.	0	1	2	3	4
8 Casi todo me preocupa	0	1	2	3	4
9 Soy una persona muy emocional.	0	1	2	3	4
10 Temo estar solo en la vida más que cualquier otra cosa	0	1	2	3	4
11 Me quedo fijado en un único modo de hacer las cosas, incluso cuando está claro que no va a funcionar.	0	1	2	3	4
12 He visto cosas que no estaban realmente allí.	0	1	2	3	4
13 Me mantengo alejado de las relaciones románticas.	0	1	2	3	4
14 No estoy interesado en hacer amigos.	0	1	2	3	4
15 Me irrito con facilidad por todo tipo de cosas.	0	1	2	3	4
16 No me gusta llegar a establecer lazos muy cercanos con la gente	0	1	2	3	4
17 No es tan grave herir los sentimientos de alguien.	0	1	2	3	4
18 Rara vez me siento entusiasmado por algo.	0	1	2	3	4
19 Deseo atención	0	1	2	3	4
20 A menudo tengo que lidiar con personas que son menos importantes que yo.	0	1	2	3	4
21 A menudo tengo pensamientos que tienen sentido para mí, pero que según otras personas resultan extraños.	0	1	2	3	4
22 Yo uso a la gente para conseguir lo que quiero.	0	1	2	3	4
23 A veces me ausento mentalmente y de repente “vuelvo” y me doy cuenta de que pasó mucho tiempo.	0	1	2	3	4
24 A menudo siento que las cosas que me rodean parecen "irreales", o más "reales" que lo habitual.	0	1	2	3	4
25 Me resulta fácil sacar ventaja de los demás.	0	1	2	3	4

Referencias de los Cuestionarios del Estudio:

1) **Cuestionarios sobre consumo de sustancias (uso de drogas y alcohol) Brief tool+AUDIT+DAST-10 modificados**

Tiet QQ, Leyva YE, Frayne SM, Smith B. Screen of Drug Use (SoDU): Diagnostic accuracy of a new brief tool for primary care. Presented at: VA HSR&D National Meeting; 2015 Jul 10; Philadelphia, PA

Rubio G, Bermejo J, Caballero MC, Santo-Domingo J. Validación de la prueba para la identificación de trastornos por uso de alcohol (AUDIT) en atención primaria. Rev Clin Esp. 1998; 198:11-4.

Pérez Gálvez, B., García Fernández, L., Oliveras Valenzuela, M. A., Lahoz Lafuente, M., & Vicente Manzanero, M. P. d. (2010). Validación española del Drug Abuse Screening Test (DAST-20 y DAST-10). INID, Instituto de Investigación de Drogodependencias

2) **Cuestionario sobre comportamiento sexual**

Marcus JL, Glidden DV, Mayer KH, Liu AY, Buchbinder SP, et al. (2013) No Evidence of Sexual Risk Compensation in the iPrEx Trial of Daily Oral HIV Preexposure Prophylaxis. PLoS ONE 8(12): e81997. doi: 10.1371/journal.pone.0081997

3) **Cuestionario psicosocial basal**

Socías, M. E., Marshall, B. D. L., Arístegui, I., Zalazar, V., Romero, M., Sued, O., & Kerr, T. (2014). Towards Full Citizenship: Correlates of Engagement with the Gender Identity Law among Transwomen in Argentina. PLoS ONE, 9(8), e105402. http://doi.org/10.1371/journal.pone.0105402

4) **Cuestionario psicosocial 6 y 12 meses**

Socías, M. E., Marshall, B. D. L., Arístegui, I., Zalazar, V., Romero, M., Sued, O., & Kerr, T. (2014). Towards Full Citizenship: Correlates of Engagement with the Gender Identity Law among Transwomen in Argentina. PLoS ONE, 9(8), e105402. http://doi.org/10.1371/journal.pone.0105402

6) **Cuestionario de Calidad de Vida: Well-being Index**

Tonon, G. & Aguirre, V. (2002–2009). *Argentine version well-being index*. Australia: International Well-being Group, Australian Center of Quality of Life, Deakin University. Retrieved February 9, 2011 from http://www.deakin.edu.au/research/acqol/auwbi/index-translations/wbi-spanish-argentina.pdf

7) **Cuestionario de Apoyo social: Social Support Scale Duke**

Bellón, J. A., Delgado, A., De Dios, J. & Lardelli, P. (1996). Validez y fiabilidad del cuestionario de apoyo social funcional Duke-UNC-11. Atención Primaria, 18, 153-163.

8) **Cuestionario sobre Depresión e ideación suicida: Center for Epidemiological Studies – Depression scale (CES-D)+ 4-item suicidal-ideation screener**

PA Defechereux, M Mehrotra, AY Liu, RM Grant, et al. Depression and Oral FTC/TDF Pre-exposure Prophylaxis (PrEP) Among Men and Transgender Women Who Have Sex With Men (MSM/TGW). AIDS and Behavior. June 16, 2015 (Epub ahead of print).

Clements-Nolle, K., Marx, R., Guzman, R., Katz, M. HIV prevalence, risk behaviors, health care use, and mental health status of transgender persons: Implications for public health intervention. American Journal of Public Health, 2001. 91: p. 915-921.

Budge, S. & Adelson, J. (2013). Anxiety and depression in transgender individuals: The roles of transition status, loss, social support, and coping. Journal of Consulting and Clinical Psychology, 81, 545-557

Radloff LS. The CES-D scale: a self-report depression scale for research in the general population. Applied Psychological Measurement. 1977;1:385-401

9) Cuestionario sobre Ansiedad: Inventario de ansiedad-rasgo STAI

Spielberger, C. D., Gorsuch, R. L. y Lushene, R. E. (2008). STAI. Cuestionario de ansiedad estado-rasgo (7ª ed. rev.). Madrid: TEA

10) Escala de Estigma en VIH: Berger HIV Stigma Scale

Franke, M. F., Munoz, M., Finnegan, K., Zeladita, J., Sebastian, J. L., Bayona, J. N. et al. (2010). Validation and Abbreviation of an HIV Stigma Scale in an Adult Spanish-Speaking Population in Urban Peru. Aids and Behavior, 14, 189-199.

Berger, B. E., Ferrans, C. E., & Lashley, F. R. (2001). Measuring stigma in people with HIV: Psychometric assessment of the HIV stigma scale. Research in Nursing & Health, 24, 518-529.

11) Cuestionario sobre Personalidad: Inventario de personalidad para el DSM-5 (PID-5 Abreviado) – Adulto

Krueger RF, Derringer J, Markon KE, Watson D, Skodol AE. (2013). The Personality Inventory for DSM-5 Brief Form (PID-5-BF). Manuscript in preparation.

14 Referencias

1. Baral SD, Poteat T, Stromdahl S, Wirtz AL, Guadamuz TE, Beyrer C. Worldwide burden of HIV in transgender women: a systematic review and meta-analysis. The Lancet Infectious diseases. 2013 Mar;13(3):214-22. PubMed PMID: 23260128.

2. Poteat T, Reisner SL, Radix A. HIV epidemics among transgender women. Current opinion in HIV and AIDS. 2014 Mar;9(2):168-73. PubMed PMID: 24322537.

3. Operario D, Nemoto T. HIV in transgender communities: syndemic dynamics and a need for multicomponent interventions. Journal of acquired immune deficiency syndromes. 2010 Dec;55 Suppl 2:S91-3. PubMed PMID: 21406995. Pubmed Central PMCID: 3075534.

4. Kerr T SE, Sued O. HIV Infection among Transgender Women: Challenges and Opportunities. J AIDS Clin Res. 2014.

5. Nemoto T, Operario D, Keatley J, Villegas D. Social context of HIV risk behaviours among male-to-female transgenders of colour. AIDS care. 2004 Aug;16(6):724-35. PubMed PMID: 15370060.

6. Reback CJ FJ. HIV prevalence, substance use, and sexual risk behaviors among transgender women recruited through outreach. AIDS and Behavior. 2014.

7. Nemoto T, Bodeker B, Iwamoto M. Social support, exposure to violence and transphobia, and correlates of depression among male-to-female transgender women with a history of sex work. American journal of public health. 2011 Oct;101(10):1980-8. PubMed PMID: 21493940. Pubmed Central PMCID: 3222349.

8. Dirección Nacional de Sida, Boletin epidemiológico 2010. Available at http://www.msal.gov.ar/sida/pdf/investigaciones/resumen_ejecutivo.pdf.

9. Carobene M, Bolcic F, Farias MS, Quarleri J, Avila MM. HIV, HBV, and HCV molecular epidemiology among trans (transvestites, transsexuals, and transgender) sex workers in Argentina. Journal of medical virology. 2014 Jan;86(1):64-70. PubMed PMID: 24123155.

10. Dos Ramos Farias MS, Garcia MN, Reynaga E, Romero M, Vaulet ML, Fermepin MR, et al. First report on sexually transmitted infections among trans (male to female transvestites, transsexuals, or transgender) and male sex workers in

Argentina: high HIV, HPV, HBV, and syphilis prevalence. International journal of infectious diseases : IJID : official publication of the International Society for Infectious Diseases. 2011 Sep;15(9):e635-40. PubMed PMID: 21742530.

11. Socias M, Marshall B, Aristegui I, Romero M, Cahn P, Kerr T, et al. Factors associated with healthcare avoidance among transgender women in Argentina. International journal for equity in health. 2014 Sep 27;13(1):81. PubMed PMID: 25261275. Pubmed Central PMCID: 4220051.

12. Socias ME, Marshall BD, Aristegui I, Zalazar V, Romero M, Sued O, et al. Towards full citizenship: correlates of engagement with the gender identity law among transwomen in Argentina. PloS one. 2014;9(8):e105402. PubMed PMID: 25133547. Pubmed Central PMCID: 4136870.

13. Cohen MS, McCauley M, Gamble TR. HIV treatment as prevention and HPTN 052. Current opinion in HIV and AIDS. 2012 Mar;7(2):99-105. PubMed PMID: 22227585. Pubmed Central PMCID: 3486734.

14. Young I, Flowers P, McDaid LM. Key factors in the acceptability of treatment as prevention (TasP) in Scotland: a qualitative study with communities affected by HIV. Sexually transmitted infections. 2014 Dec 7. PubMed PMID: 25482649.

15. Wilson EC, Garofalo R, Harris RD, Herrick A, Martinez M, Martinez J, et al. Transgender female youth and sex work: HIV risk and a comparison of life factors related to engagement in sex work. AIDS and behavior. 2009 Oct;13(5):902-13. PubMed PMID: 19199022. Pubmed Central PMCID: 2756328.

16. Keller K. Transgender health and HIV. BETA : bulletin of experimental treatments for AIDS : a publication of the San Francisco AIDS Foundation. 2009 Summer-Fall;21(4):40-50. PubMed PMID: 20034245.

17. Socías ME SO, Frola C, Iacchetti A, Kerr T, Arístegui I, Zalazar V, Pérez H, Cahn P. Engagement in the HIV care cascade among transgender women enrolled in a public HIV clinic in Buenos Aires, Argentina, 2000-2012. 20th International AIDS Conference (AIDS 2014); July, 20-25; Melbourne, Australia2014. 2014.

18. Clotet B, Feinberg J, van Lunzen J, Khuong-Josses MA, Antinori A, Dumitru I, et al. Once-daily dolutegravir versus darunavir plus ritonavir in antiretroviral-naive adults with HIV-1 infection (FLAMINGO): 48 week results from the randomised open-

label phase 3b study. Lancet. 2014 Jun 28;383(9936):2222-31. PubMed PMID: 24698485.

19. Masho SW, Wang CL, Nixon DE. Review of tenofovir-emtricitabine. Therapeutics and clinical risk management. 2007 Dec;3(6):1097-104. PubMed PMID: 18516268. Pubmed Central PMCID: 2387297.
