# Supplementary material for: Retention among transgender women treated with dolutegravir associated with tenofovir/lamivudine or emtricitabine in Argentina: TransViiV study
Source: PLoS One. 2023 Jan 20;18(1):e0279996. doi: 10.1371/journal.pone.0279996 (PMC9858466; doi:10.1371/journal.pone.0279996)
Supplement: S2 File — (PDF) [file pone.0279996.s002.pdf]

Treatment with Dolutegravir associated with Tenofovir/Lamivudine or Emtricitabine in trans women infected with HIV: a pilot study

Principal Investigator: Dr. Omar Sued

Institution: Fundación Huésped

Address: Ángel Peluffo 3932 (C1202ABB)

Ciudad Autónoma de Buenos Aires., Argentina

Phone: +54 11 4981-1855/7777

FAX: +54 11 4982 4024

E-mail: omar.sued@huesped.org.ar

1 Clinical Study Protocol

Treatment with Dolutegravir associated with Tenofovir/Lamivudine or Emtricitabine in trans women infected with HIV: a pilot study

Protocol Number	FH-17
Product Research	Dolutegravir-Tenofovir-Emtricitabine
Date	22/JUL/2015
Study Design	Prospective, open-label, single arm exploratory trial
Researchers	Omar Sued, MD, PhD
Sponsor	Fundación Huésped[1*]

This study will be conducted in accordance with the protocol, Good Clinical Practices and other applicable regulatory requirements, including archiving of essential documents.

[1*] This study will be performed with an unrestricted grant ViiV Laboratory, Middlesex, United Kingdom.

2 Index

1 22 33
54 **¡Error! Marcador no definido.**4.1
84.1.1 84.1.2
84.2 84.3
95 95.1
95.2 95.3
105.4 105.5
105.6 115.7
125.8 125.9
126 136.1
136.2 146.3
156.4 156.5
156.5.1 156.5.2
166.6 176.7
187 187.1
187.2 197.3
207.4 **¡Error! Marcador no definido.**7.4.1
227.5 227.6
227.6.1 237.6.2
237.7 237.8
257.9 267.10
¡Error! Marcador no definido.8 268.1
278.1.1 278.1.2
288.1.3 288.1.4
289 **¡Error! Marcador no definido.**10
2810.1 **¡Error! Marcador no definido.**10.2
¡Error! Marcador no definido.11 2912
2913 **¡Error! Marcador no definido.**13.1
¡Error! Marcador no definido.13.2 3113.3
3513.4 3613.5
3813.6 3913.7
4013.8 4113.9
4314 45

3 Introduction

Transgender women (transwomen) remain a key population within the global HIV pandemic. Transwomen contend with exceptionally high rates of HIV infection, driven concomitantly by drug use and sexual pathways (1-6). These risks are exacerbated by various social and structural conditions, including violence, stigma and discrimination (7). At a global level, recent estimates suggest that transwomen have 49 times the risk of HIV infection compared to the general adult population (1). Thus, there is an urgent need for novel research that identifies and informs interventions—operating at the individual, social, and structural levels—with the potential to reduce HIV morbidity and transmission among transwomen.

Argentina has a concentrated HIV epidemic, with 0.4% prevalence in the general population and a higher prevalence among specific groups with the transgender community being the most affected. HIV prevalence in transwomen is 34%, markedly higher than in men who have sex with men (11.4%), drug users (7%) and female sex workers (6%) (8). Vulnerability is remarkablyly high among transwomen with a life expectancy of approximately 35 years in Buenos Aires which contrasts with a life expectancy of 79 in other women (9, 10). Use of drugs, sex work, experiences of sexual violence, unsafe plastic implants, legal problems, tuberculosis and other infectious diseases such as syphilis and hepatitis, and mental health problems, are also more frequent among transwomen, which is related to stigma and contributes to higher morbidity and mortality in this population. Internal and gender-related stigma, discrimination, particularly within the health care sector, are barriers to access to health care services (11).

The high risk of this population is exemplified by the result of a recent nation-wide survey among 452 transwomen. Self-reported HIV prevalence was 27% but it was twice as high among those who reported inconsistent condom use with clients, unstable housing and experiences of police violence highlighting the complexities to address traditional HIV prevention methods among this population (12).

Increasing evidence demonstrates that provision of HIV treatment to HIV positive individuals, in addition to preventing disease progression and death, greatly reduces transmission to sex partners (13). Universal testing and treatment to prevent transmission of HIV has gained considerable attention due to their public health benefits. However it requires quick access to simple and safe antiretroviral combinations, routine HIV testing made available to most of

the population, and the feasibility of immediate treatment for those who are identified as being HIV infected (14). The benefits of this approach—known as treatment as prevention (TasP)—would provide even greater benefits when targeted to high-risk and mobile populations, such as transwomen, representing one of the largest groups of the epidemic in Latin America. However, despite these potential benefits, unanswered questions about its operational feasibility and acceptability continue to generate debate.

The contextual risk environment where transwomen live, work and socialize include alcohol and drug use, sex work, and high exposure to violence, discrimination and stigma, all of which may jeopardize ART expansion and may function as barriers to adherence, limiting the efficacy of this approach (15). In addition, self-prescription of hormones and other treatments, a common practice within the transgender community, may result in drug-drug interactions, further undermining ART effectiveness (16). Among 69 HIV positive transwomen enrolled in our HIV clinic, only 46% started ART within the first year of HIV diagnosis. Among those who were prescribed ART, only 38% could maintain virological suppression after 48 weeks of treatment (16% of the total) highlighting the need to better understand social, structural and environmental factors affecting HIV outcomes among high risk populations (17).

In light of the aforementioned considerations, there is an urgent need to explore the potential risks and benefits of the TasP approach among transwomen. Some of the critical research questions include: a) which is the most suitable regimen in terms of potency, dosing schedule, tolerability, side effects and drug-drug interactions?, b) what is the acceptability of ART among transwomen?, and c) what are the other contextual and individual factors that might facilitate or impede acceptability, adherence and retention in ART regimens among transwomen.

Although developing countries are not considering integrase inhibitors (INIs) as first line therapy, Dolutegravir (DTG) based regimens should be considered as preferred first-line regimens for this population for a number of reasons. DTG is an extremely potent antiviral compound with proven clinical efficacy in both treatment-naïve (patients who never received ART previously) and treatment-experienced HIV-infected patients, with a demonstrated higher genetic barrier than other INIs (18). The excellent safety and tolerance profile might facilitate better adherence than other regimens in this particular population and the lack of significant drug-drug interactions makes DTG-based combinations eligible for individuals with

significant co-morbidities or who are potentially using other drugs. In addition, the possibility of once-daily dosing makes DTG-based regimens extremely attractive for this population.
Tenofovir disoproxil fumarate-emtricitabine (TDF-FTC) or lamivudine (TDF-3TC) are a one-pill daily dose of NtRTI/NRTI combination that have demonstrated efficacy in well-designed clinical trials with a good follow up. TDF-FTC or TDF-3TC combine the benefits of less toxicity, dosing simplicity, and favorable pharmacokinetic properties (19).
Although a triple drug combination (DTG, abacavir, lamivudine) will be available in the near future, the difficulties in having a timely access to HLA B5701 testing to rule out abacavir hypersensitivity could limit its indication.
We therefore hypothesized that a DTG-TDF-FTC or 3TC regimen would be an effective regimen in antiretroviral naïve, HIV-1-infected transwomen that might have a good acceptability, tolerability and safety profile, and that it will result in a significant percentage of individuals being able to maintain this regimen for at least 48 weeks.

4 Trial Objectives

4.1 Objectives

4.1.1 Primary Objective

The **primary objective** of this pilot study is to determine the retention in care among transwomen treated with DTG-TDF-FTC or 3TC.

4.1.2 Secondary Objectives

a. To evaluate the efficacy of the antiretroviral regimen at 48 weeks;
b. To describe the safety and tolerability of this regimen;
c. To evaluate adherence across 48 weeks;
d. To determine the patient's satisfaction with this regimen;
e. To identify individual, social and contextual factors associated with adherence and retention.

4.2 Primary Endpoint

The primary objective will be assessed by the proportion of individuals that provide information on ART use at the end of the study:

a. Retention under care: Proportion of enrolled and dosed individuals that provide clinical information up to 48 weeks of follow up.

b. Retention in treatment: Proportion of enrolled and dosed individuals that receive study drugs up to 48 weeks of follow up.

4.3 Secondary Endpoints

The secondary objectives will be evaluated using the following endpoints:

a. Proportion of patients with HIV-q RNA levels less than 50 copies/mL in an intention-to-treat analysis at week 48 using the FDA Snapshot algorithm (missing data, change or discontinuation of treatment are considered failure) in patients who have taken at least one dose of the medicine

b. Frequency, type and severity of adverse events and laboratory abnormalities;

c. Pill count, analogue visual scale for adherence in each visit;

d. Changes in the scores of quality of life, social support, anxiety and depression scales (WBI, DUKE, HAD) at baseline, 4, 24, and 48 weeks of follow up;

e. Associations between baseline individual, social and contextual characteristics and percentage of adherence and retention at 48 weeks.

5 Investigation Plan

5.1 Overall Study Design and Plan: Description

Prospective, open-label, single-arm trial of DTG-TDF-FTC or 3TC among ART-naïve HIV.

Procedures (see Table 2): Patients will be actively identified through community testing campaigns and access to HIV services at Fundación Huésped will be facilitated. After consenting to participation, participants will be screened and will receive a new appointment for ART initiation (if eligible).

5.2 Trial Population

Subjects will be ≥18 years HIV-1 patients, HIV treatment-naïve, self-identified as transwomen who met all inclusion criteria and none of the exclusion criteria.

5.3 Sample Size

As a pilot, proof-of-concept study that might eventually be followed by a larger multicentric study; no formal sample size will be calculated. A convenience sample size of 60 HIV-naïve transwomen was selected based on the feasibility to enroll such a population in one site. This pilot study will allow assessing the protocol design and supporting the development and validation of individual assessment forms to be used in further, larger clinical trials. Additional details are commented upon in the Statistical Analysis Plan.

5.4 Inclusion Criteria

A subject will be eligible for study participation if she meets ALL of the following criteria:

a. HIV-1 positive serology by at least two different serological tests (rapid test, ELISA, Western Blot) or a viral load higher than 3,000 copies/mL.

b. 18 years and older.

c. Self-identified as transwomen.

d. Antiretroviral naïve.

e. Written informed consent provided.

5.5 Exclusion Criteria

Patients will NOT be eligible to participate in this study if they meet ANY of the following criteria:

a. Genotypic resistance to emtricitabine and/or tenofovir as per IAS-USA resistance panel 2013.

b. Alcohol or drug use that might affect adherence.

c. Concomitant use of lipid-lowering drugs, interferon, interleukin-2, cytotoxic chemotherapy, dofetilide (or pilsicainide) or immunosuppressors, antacids drugs containing Ca++ and or Mg++ at study entry.

d. Opportunistic infection (CDC “C” category) or other disease and/or clinical conditions that, in the investigator's opinion, would compromise the patient's safety or outcome of the study; including malignancy other than cutaneous Kaposi's sarcoma, basal cell carcinoma, or non-invasive cutaneous squamous cell carcinoma, or cervical intraepithelial neoplasia.

e. Treatment with any of the following agents within 28 days of screening: radiation therapy; cytotoxic chemotherapeutic agents; any immunomodulators that alter immune responses or treatment with an HIV-1 immunotherapeutic vaccine within 90 days of screening or exposure to an experimental drug or experimental vaccine within either 28 days, 5 half-lives of the test agent, or twice the duration of the biological effect of the test agent, whichever is longer, prior to the first dose of the investigational product.

f. Contraindication to any of the study drugs (history of renal diseases, lab abnormalities grade 4 or any other clinical condition prior therapy that, in the opinion of the investigator, would make the subject unsuitable for the study or unable to comply with the dosing requirements).

g. Anticipated need for Hepatitis C virus (HCV) therapy during the study.

h. Creatinine clearance of <50 mL/min via Cockroft-Gault method.

i. Subjects with moderate to severe hepatic impairment (Class B or greater) as determined by Child-Pugh classification.

5.6 Removal of Subjects from Therapy or Assessment

Patients <u>may be</u> withdrawn from the study if:

a. A serious adverse event (SAE) occurs;

b. The subject no longer derives clinical benefit from the treatment according to the investigator's opinion.

Patients <u>must be</u> withdrawn from the study if:

a. Patient withdraws consent;

b. The patient has a confirmed viral load ≥1,000 copies/mL at week 24 or at week 36;

c. The patient has confirmed rebound in viral load ≥200 copies/mL after reaching undetectable VL (<50 copies/mL);

d. The patient has any of the rules of detention due to liver damage; (see Section 7.6.2);

e. The researcher considers that, for safety reasons, it is best for the patient to be withdrawn from the study;

f. Subjects with allergic reaction grade ≥3 considered possibly or probably related to research drugs;

g. Rash grade 3 or 4;

h. Any rash associated with increased ALT ≥2.

The date and reason for discontinuation must be registered in the case report form (CRF). Unless the patient withdraws consent, the patient must make a final study visit/discontinuation.

5.7 Treatment

Dolutegravir 50 mg QD plus co-formulated lamivudine 300 mg or emtricitabine 200 mg/tenofovir 300 mg QD, once a day with food.

5.8 Identity of the Investigational Product

<u>Dolutegravir:</u>
Dosage form: 50 mg film coated tablet
Dose and regimen: 50 mg once daily

<u>Emtricitabine (FTC) or Lamivudine (3TC):</u>
Dosage form: 200 mg or 300mg respectively, co-formulated with TDF tablet

<u>Tenofovir (TDF):</u>
Dosage form: 300 mg co-formulated with FTC or 3TC tablet

Dose and regimen: TDF-FTC or 3TC a co-formulated pill once a day.
Dolutegravir will be provided by ViiV. TDF-FTC or 3TC will be provided by the Ministry of Health or the regular provider.

5.9 Definition of Virological Failure

Virological failure is defined as:

a. Confirmed viral rebound ≥200 copies after having reached <50 copies/mL;

b. Confirmed viral load ≥1,000 copies/mL at week 24;

c. Confirmed viral load ≥50 copies/mL at week 48.

If a patient reaches one of these situations of virological failure, viral load should be repeated with a new sample obtained not less than 7 and no more than 30 days later. If virological failure is confirmed, a genotypic resistance test that evaluates integrase inhibitors susceptibility should be conducted to assess the number and type of resistance mutations.

6 Study Procedures

After the subject signs the study-specific informed consent, she will undergo the following procedures:

6.1 Screening Procedures

a. Medical History. A complete medical history, including history of tobacco, drug and alcohol use, opportunistic infections and drug adverse events will be obtained.

b. Complete Physical Examination, including height and weight measurements, and vital signs in sitting position (blood pressure, pulse and body temperature).

c. Laboratory**:**

- Plasma HIV-1 RNA level, measured by Abbott real time PCR.
- CD4+ and CD8+ cell count.
- Clinical laboratory tests: hematology, complete chemical, urine, and serology as listed in Table 1 and 2.
- HIV-1 genotype (conventional resistance test).

d. EKG: **A 12-lead resting EKG will be obtained at the Screening Visit and as clinically indicated during the study, a qualified physician from the site will interpret,**

sign, and date all EKG tracings. The global interpretation will be stated as a written comment on the tracing using the following categories:

- Normal EKG
- Abnormal EKG - not clinically significant
- Abnormal EKG - clinically significant

e. Concomitant medications, including supplements and self-prescribed drugs.

The results of all clinical evaluations during screening must be within clinically acceptable limits as defined by the laboratory tests and reviewed by the investigator.

6.2 Visit Procedures at Baseline, Week 24 and 48

a. An updated medical history will be obtained prior to the study drug administration.

b. Complete Physical Examination. The physical examination at the Baseline Visit will serve as the baseline physical examination for the entire study.

c. Weight

d. Vital signs: determinations of sitting blood pressure, pulse and body temperature

e. Laboratory:

- Plasma HIV-1 RNA level, measured by Abbott real Time PCR.
- CD4+ and CD8+ cell count.
- Plasma and cell storage.
- Clinical laboratory tests: hematology, complete chemical and urine as listed in Table 1 and 2.

f. Concomitant medications: Medication (prescribed and over-the-counter) will be updated and recorded.

g. Adherence (Pill count, and self-reported ACTG Questionnaire Baseline version for Baseline Visit and Follow-Up Version for following visits will be provided).

h. Adverse events assessment.

i. Questionnaires: sexual behavior, drug use; stigma and discrimination, quality of life (Well-Being Index, WBI), social support (Social Support Scale Duke-UNC) and anxiety and depression (The Hospital Anxiety and Depression Scale, HAD).

6.3 Visit Procedures at Week 4, 8, 12 and 36

a. Symptom-directed physical examination:

- Weight
- Vital sign determinations of sitting blood pressure, pulse and body temperature

b. Laboratory:

- Plasma HIV-1 RNA level measured by Abbott real Time PCR
- CD4+ and CD8+ cell count
- Clinical laboratory tests: hematology, complete chemical, and urine as listed in Table
- Plasma storage

c. Concomitant medications

d. Adherence (Pill count, and self report ACTG Follow-Up questionnaire)

e. Adverse events assessment

6.4 Procedures at Final/Discontinuation Visit

a. Complete physical examination

b. Weight

c. Vital sign determinations of sitting blood pressure, pulse and body temperature

d. Adverse events assessment

6.5 Laboratory Tests

Ten-hour fasting blood samples (water is permitted) will be required for Baseline Visit and Week 24 and 48 Visits. For remaining visits, 8 hours fasting will be enough (see laboratory tests listed in Table 1 and specified visits in Table 2). All laboratory test results that meet the criteria for Grade 3 or 4 toxicities or are considered clinically significant by the investigator will be followed until their resolution. The investigator must review, sign and date all lab reports.

6.5.1 Table 1. Clinical Laboratory Tests

Hematology	Clinical Chemistry	Urinalysis	Additional Procedures
Hematocrit<br>Hemoglobin<br>Red Blood Cell (RBC) count<br>White Blood Cell (WBC) count<br>Neutrophils<br>Lymphocytes<br>Monocytes<br>Basophils<br>Eosinophils<br>Platelet count<br><br>,	Urea<br>Creatinine<br>Creatine phosphokinase<br>Total bilirubin<br>Direct bilirubin<br>Indirect bilirubin<br>(SGPT/ALT)<br>(SGOT/AST)<br>Alkaline phosphatase<br>Total cholesterol<br>Triglycerides<br>HDL<br>LDL<br>Total protein<br>Glucose (fasting)<br>Albumin<br>Serum amylase<br>Gamma-GT<br>Sodium<br>Potassium<br>Calcium<br>Chloride<br>Bicarbonate<br>LDH	Specific gravity<br>Ketones<br>pH<br>Protein<br>Blood<br>Glucose<br>Urobilinogen<br>Microscopic-evaluation	HBsAg<br>Anticore HBV<br><br>HCV Ab<br><br>Plasma HIV-1 RNA<br><br>HIV-1 drug resistance genotype<br><br>CD4+ T-cell count<br>CD8+ T-cell count<br><br>Plasma storage<br><br>Cell Storage

6.5.2 Table 2. Schedule of Procedures

Procedures	SCR	BSL	Week 4	Week 8	Week 12	Week 24	Week 36	Week 48
Informed Consent	X							
Medical History	X							
Physical Exam	X	X	X	X	X	X	X	X
Vital Signs	X	X	X	X	X	X	X	X
EKG	X							
Concomitant Medications	X	X	X	X	X	X	X	X
WB questionnaires[1]		X	X			X		X
Psychosocial questionnaires		X				X		X
Adverse Events and Adherence		X	X	X	X	X	X	X
HIV Genotype*	X							
Serologies[2]	X							
HIV-1 RNA	X	X		X	X	X	X	X
Flow cytometry (CD4 and CD8 cell count)	X	X			X	X	X	X
Complete blood count (CBC)[3]	X	X	X		X	X	X	X
Basic Chemistry[4]	X		X		X		X	
Complete chemistry[5]		X				X		X
Other safety markers[6]		X				X		X
Plasma storage		X	X	X	X	X	X	X

1) WBI, DUKE, CSE, HAD
2) HBV anti core, HBsAg, HCV IgG, VDRL
3) hemoglobin, hematocrit, erythrocytes, WBC (leukocytes), thrombocytes, neutrophils, basophils, lymphocytes, monocytes, reticulocytes, MCV, MCH, MCHC, platelets, prothrombin time (PT expressed in INR), activated thromboplastin time (aPTT)
4) ALT, AST, total bilirubin, creatinine,
5) ALT, AST, alkaline phosphatase, amylase, gamma-GT, sodium, potassium, calcium, chloride, bicarbonate, total bilirubin, direct bilirubin, indirect bilirubin, lactate, creatinine, urea, total cholesterol, HDL chol, LDL chol, triglycerides, lactate dehydrogenase (LDH), total protein, albumin, urine dipstick
6) 17 B estradiol, testosterone free and total, D-dimer, hsPCR, IL6, sCD14, sCD163, ADMA and hormonal levels

6.6 Prior and Concomitant Therapy

All medications (prescribed or over the counter) taken by the patient will be recorded and the subject will be instructed to discontinue all prohibited medication before baseline visit. Subjects will be provided with a list of medications that should not be taken during the study (Appendix C) and advised to avoid contraindicated medications.

Any medication (including over-the-counter medicines such as aspirin, antacids, vitamins, mineral supplements and herbal preparations) that the subject is receiving at the time of enrollment, and during the study, must be recorded together with route and dates of administration and dosages.

6.7 Treatment Adherence

All subjects will receive ongoing counseling regarding the importance of adherence to their medication. They will be instructed to return all bottles of study drugs and remaining pills if any, to the study coordinator at all study visits following the Baseline Visit. A pill count at every visit will be used to assess adherence to treatment. Adherence will be further assessed with the ACTG Adherence Questionnaire at each study (see table 2) visit. Baseline ACTG Adherence Questionnaire will be used. ACTG Adherence Questionnaire at the rest of the visits (Appendix I).

7 Safety Variables

Safety will be assessed by adverse events, physical examination, vital signs and clinical laboratory data.

7.1 Adverse Events/Experiences

Throughout the course of the study (which begins when the Informed Consent is signed), the investigator will monitor each subject for the development of any clinical and/or laboratory evidence of an Adverse Event/Experience (AE). An adverse event/experience is defined as any undesirable medical occurrence in a subject who participates in a study and includes those events/experiences which do not necessarily have a causal relationship to the study drug regimen. Prior to the administration of study combination, only adverse events/experiences that meet the definition of serious (Section 8.3) and adverse events that the investigator considers to be related to study design and/or procedures will be reported.
An adverse event may be a symptom, sign, or abnormal laboratory finding. Any worsening of a pre-existing condition or intercurrent illness should be reported as an adverse event/experience. A laboratory abnormality should be reported as an adverse event/experience if action is required (e.g. study drug interruption, discontinuation, or treatment is required). The nature of the adverse sign or symptom, its date and time of onset, duration and severity, therapy employed (if any) and the investigator’s opinion of causality to

study drug with an alternate etiology, if appropriate, must be documented. For adverse events/experiences to be considered as intermittent or continuous, the events should be of similar nature and severity.

The investigator will follow all adverse events to satisfactory clinical resolution or the establishment of a stable chronic stage upon study completion.

The investigator will rate the severity of the adverse event according to the following definition:

Mild: The adverse event is transient and easily tolerated by the subject.

Moderate: The adverse event causes the subject discomfort and interrupts the subject's normal activities.

Severe: The adverse event causes considerable interference with the subject's normal activities, and may be incapacitating or life-threatening.

The investigator will use the following definitions to assess the relationship of the adverse event to study drug:

Probable: An adverse event has a strong temporal relationship to study drug or recurs on rechallenge, and another etiology is unlikely or significantly less likely.

Possible: An adverse event has a strong temporal relationship to the study drug, and an alternative etiology is equally or less likely compared to the potential relationship to study drug. The alternate etiology should be provided by the investigator.

Probably Not: An adverse event has little or no temporal relationship to the study drug and/or a more likely alternative etiology exists. The alternate etiology should be provided by the investigator.

Not Related: An adverse event is due to underlying or concurrent illness or the effect of another drug and is not related to the study drug. The alternate etiology should be provided by the investigator.

7.2 Lipodystrophy

Fat redistribution and Cushingoid appearance without Cushing's disease have been reported as effects that have been seen in HIV-infected/AIDS patients receiving potent antiretroviral

therapy including the commercially available protease inhibitors. To date, the mechanisms leading to and the long-term consequences of these effects are unknown. A causal relationship to the protease inhibitors has not been established. Such conditions have been grouped under the heading of “lipodystrophy”. However, at present no consensus definition exists. Therefore, for the purposes of adverse event reporting within this study, the following conditions should be reported as discrete events rather than being clustered under the term “lipodystrophy”:

a. Peripheral fat-wasting (including face, buttocks, and limbs)
b. Central adiposity
c. Breast hypertrophy
d. Dorsal fat pad (“buffalo hump”)
e. Multiple lipomas
f. Cushingoid appearance without Cushing’s disease

Metabolic abnormalities such as hyperlipidemia or hyperglycemia have not been consistently observed with abnormalities of fat distribution and should not be labeled as lipodystrophy for adverse event reporting purposes.

7.3 Allergic Reaction

Subjects may continue to use investigational product (IP) if Grade 1 or 2 allergic reactions at the discretion of the Investigator. The subject should be advised to contact the Investigator immediately if there is any worsening of symptoms or if further systemic signs or symptoms develop. Antihistamines, topical corticosteroids, or antipruritic agents may be prescribed. Subjects with Grade ≥3 allergic reactions that are considered to be possibly or probably related to the IP should permanently discontinue the IP regimen and the subject should be withdrawn from the study. Subjects should be treated as clinically appropriate and followed until resolution of the AE.

7.4 Serious Adverse Events

The investigator will inform the Sponsor, the local IRBs and the appropriate Regulatory Agency within 24 hours of any serious adverse event reported in this study. A SAE is an adverse drug experience that results in any of the following outcomes:

a. **Death.**

b. **Life-threatening situation** — the subject was at risk of death at the time of the adverse event/experience. It does not refer to the hypothetical risk of death if the AE were more severe or were to progress.

c. **Inpatient hospitalization or prolongation of existing hospitalization**.

d. **Persistent or significant disability/incapacity** — Any AE having an outcome that is associated with a substantial disruption of the ability to carry out normal life functions, including the ability to work. This is not intended to include transient interruption of daily activities.

e. **Important medical events/experiences** that may not result in death, be life-threatening, or require hospitalization may be considered a serious adverse event/experience when, based on appropriate medical judgment, **they may jeopardize the subject and may require medical or surgical intervention to prevent one of the outcomes listed above,** i.e., death, a life-threatening adverse event/experience, inpatient hospitalization or prolongation of existing hospitalization, a persistent or significant disability/incapacity. Examples of such medical events/experiences include allergic bronchospasm requiring intensive treatment in an emergency room or at home, blood dyscrasias or convulsions that do not result in inpatient hospitalization, or the development of drug dependence or drug abuse.

Please note that a severe adverse event/experience is not necessarily serious, as the term severe is a measure of intensity while a serious adverse event is determined based on the aforementioned regulatory criteria.

The observation period for AE and SAE reporting for an individual patient will start at the time of patient screening (after Informed Consent is signed) through the Final Visit (4-week post-treatment follow-up visit) or up to 4-weeks after the last dose of treatment, whichever is later, to document late adverse events.

If the Investigator becomes aware of a drug-related SAE after the Final Visit, a SAE report form should be completed. If the Study site has been closed, the Sponsor´s Study Medical Monitor or his designee should be contacted.

7.4.1 Table 3. Events Reporting Time Periods for SAE

	Initial reports		Follow up information	
**Type of event**	**Time frame**	**Documents**	**Time frame**	**Documents**
All SAEs	24 hours	SAE data collection form	24 hours	Follow up SAE data collection form
ALT ≥3x ULN plus bilirubin ≥2x ULN (35 % direct)	24 hours	SAE data collection form	24 hours	Follow up SAE data collection form
ALT ≥5x ULN that persists >2 weeks	24 hours	SAE data collection form	24 hours	Follow up SAE data collection form
ALT ≥8 x ULN	24 hours	SAE data collection form	24 hours	Follow up SAE data collection form
ALT ≥3x ULN or ≥3 fold increase from baseline value with appearance or worsening of symptoms of hepatitis or hyper sensibility	24 hours	SAE data collection form	24 hours	Follow up SAE data collection form

7.5 Suicidal Ideation or Behaviors

If any subject experiences a possible suicidality-related adverse event (PSRAE) while participating in this study, which is considered by the Investigator to meet International Conference on Harmonization (ICH)-E2A definitions for seriousness, the Investigator will report the event on a SAE CRF form. A PSRAE may include, but is not limited to, an event that involves suicidal ideation, a preparatory act toward imminent suicidal behavior, a suicide attempt, or a completed suicide. The investigator will exercise his or her medical and scientific judgment in deciding whether an event is possibly suicide-related.

7.6 Toxicity Management

For the purpose of medical management, all adverse events and laboratory abnormalities that occur during the study must be evaluated by the investigator. Appendix B contains

"Clinical Toxicity Grades". This table is to be used in the grading of adverse events. All adverse events and laboratory abnormalities will be followed to satisfactory clinical resolution. The following guidelines should be used for study drug-related toxicity management for all subjects.

7.6.1 Grades 1-2

Subjects who develop a study drug-related Grade 1 or 2 adverse event or laboratory abnormalities may continue study medications (for bilirubin, ALT, AST elevation see liver chemistry stopping and follow-up criteria).

7.6.2 Grades 3-4

For bilirubin, ALT, AST elevations see liver chemistry stopping and follow-up criteria.
Subjects who develop a study drug-related Grade 3 or 4 adverse event or laboratory abnormality should consider interrupting all study medications, upon resolution of the adverse event or laboratory abnormality to within one grade level (not to exceed Grade 2) of the subject's baseline level, the subject may resume study drug dosing under the guidance of the investigator. If the subject experiences a recurrence of a study drug-related Grade 3 or 4 adverse event or laboratory abnormality after restarting study medications, the subject should interrupt all study medications again and resume study medications when the adverse event or laboratory abnormality has resolved to within one grade level (not to exceed Grade 2) of the subject's baseline.
If the subject's adverse events or laboratory abnormalities have not resolved to within one grade (not to exceed Grade 2) of his or her baseline level within eight weeks of study drug interruption, consideration should be given to discontinuing the subject from the study.
For bilirubin, ALT, AST elevation see liver chemistry stopping and follow-up criteria (section 7.7), for hyperglycemia, see section 7.8, for pancreatic amylase see section 7.9.

7.7 Liver Chemistry Stopping Rules and Follow up Criteria

Liver chemistry threshold stopping criteria have been designed to assure subject safety and to evaluate liver event etiology during administration of study drugs and the follow-up period. Study drugs will be stopped if any of the following liver chemistry criteria are met:

a. ALT ≥3xULN and bilirubin ≥2xULN (>35% direct bilirubin; bilirubin fractionation required)

b. ALT ≥8xULN;

c. ALT ≥3xULN (if baseline ALT is <ULN) with symptoms or worsening of acute hepatitis or hypersensitivity such as fatigue, nausea, vomiting, right upper quadrant pain or tenderness, fever, rash, or eosinophilia, OR;

d. ALT ≥3x baseline ALT with symptoms or worsening of acute hepatitis or hypersensitivity such as fatigue, nausea, vomiting, right upper quadrant pain or tenderness, fever, rash, or eosinophilia;

e. ALT ≥5xULN and <8xULN that persists >2 weeks (with bilirubin <2xULN and no signs or symptoms of acute hepatitis or hypersensitivity);

f. ALT ≥5xULN but <8xULN and cannot be monitored weekly for >2 weeks.

Subjects who develop ALT ≥5xULN should be followed weekly until resolution or stabilization (ALT <5xULN on two consecutive evaluations).

When liver chemistry stopping criterion is met, study medications will be immediately discontinued and the subject withdrawn from the study.
Subjects should not restart DTG due to the risk of a recurrent reaction.

a. Event must be reported to the study Sponsor within 24 hours of learning its occurrence

b. CRF should be completed and, where applicable, liver imaging and/or liver biopsy must be captured in CRFs if these tests are performed;

c. Liver event must be followed up and monitored until liver chemistries resolve, stabilize, or return to baseline values;

d. Every reasonable attempt should be made to have subjects return to clinic within 24 hours for repeat liver chemistries, liver event follow up assessments, and close monitoring;

e. A specialist or hepatology consultation is recommended;

f. Subjects should be monitored twice weekly until liver chemistries (ALT, AST, alkaline phosphatase, bilirubin) resolve, stabilize or return to within baseline values.

The following additional tests should be considered to further evaluate the liver event:

a. Viral hepatitis serology including:

- Hepatitis A IgM antibody;
- HBsAg and Hepatitis B Core Antibody (IgM);
- Hepatitis C RNA;
- Hepatitis E IgM antibody;

b. Cytomegalovirus IgM antibody;

c. Epstein-Barr viral capsid antigen IgM antibody (or if unavailable, obtain heterophile antibody or monospot testing);

d. Syphilis screening;

e. Drugs of abuse screening including alcohol;

f. Serum acetaminophen test (APAP adduct test);

g. Serum creatine phosphokinase (CPK) and lactate dehydrogenase (LDH);

h. Fractionate bilirubin, if total bilirubin is greater than 1.5xULN,

i. Obtain complete blood count with differential to assess eosinophilia;

j. Anti-nuclear antibody, anti-smooth muscle antibody, and Type 1 anti-liver kidney microsomal antibodies;

k. Liver imaging (ultrasound, magnetic resonance, or computerized tomography) to evaluate liver disease.

Record the appearance or worsening of clinical symptoms of hepatitis, or hypersensitivity, fatigue, decreased appetite, nausea, vomiting, abdominal pain, jaundice, fever, or rash as relevant on the AE report form.

Record use of concomitant medications, acetaminophen, herbal remedies, other over the counter medications, or putative hepatotoxins, on the concomitant medications report form.

7.8 Hyperglycemia

Subjects who experience study drug-related glucose elevations of Grade 3 or 4 may continue study medications, provided that appropriate management of hyperglycemia is instituted in a

timely manner. A confirmatory fasting glucose level should be obtained within two weeks after the first Grade 3 or 4 glucose elevation. Hyperglycemia may be managed with oral hypoglycemic agents or insulin as deemed clinically appropriate by the investigator.

7.9 Pancreatitis/Grade 3–4 Pancreatic Amylase

A diagnosis of pancreatitis should be considered if clinical symptoms such as nausea, vomiting, or abdominal pain are present. Patients with these signs and symptoms should be appropriately evaluated for the presence of pancreatitis through the use of diagnostic testing which may include serum amylase and lipase measurement and/or pancreatic imaging via abdominal ultrasound and/or CT scanning as clinically warranted. If a diagnosis of pancreatitis is confirmed, all drugs in the study regimen should be interrupted immediately.

If a grade 3–4 elevation of pancreatic amylase occurs during the course of the study and pancreatitis is suspected (HIV-1-infected patients can have elevations in amylase that are not necessarily of pancreatic origin), all drugs in the study regimen should be interrupted immediately. The subject should be evaluated for signs and symptoms of pancreatitis and additional diagnostic evaluation should be performed as clinically indicated.

If a diagnosis of pancreatitis is confirmed, appropriate therapy should be initiated. The investigator should not reintroduce study drug therapy until clinical signs and symptoms of pancreatitis have resolved and amylase values return to within normal range.

7.10 HIV-Related Clinical Events

HIV-infected subjects participating in clinical trials may develop conditions typically associated with AIDS. These conditions include certain malignancies, opportunistic infections, neurological dysfunction and some generalized constitutional symptoms. Appendix D contains a list of known expected manifestations of HIV infection. The events listed in Appendix D will be summarized as HIV-Related Events, not as Adverse Events.

The same form will be used to record Adverse Events and HIV-Related Events. HIV-Related Events that are serious in nature, as defined in Section 7.4, must be reported in the same manner as Serious Adverse Events.

8 Statistical Considerations

8.1 Statistical Analysis Plan

Retention under care, retention on treatment and virologic suppression will be shown as proportions of those subject included in the study (intent to treat-exposed analysis), and, of those who received at least one dose of study medication (modified intent to treat analysis). Interim analysis will include the 24 week evaluation and final analysis the 48 week results.
Descriptive statistics will be used to evaluate the primary and secondary outcomes. The results will be given as median and interquartile ranges (IQR) or frequencies (%) and IC95%, as appropriate. At each timepoint, longitudinal differences from baseline will be assessed using Student's t-test or Wilcoxon rank-sum test for paired samples, as required. A two-tailed P value <0.05 will be considered to be statistically significant. Baseline and follow-up characteristics will be compared using the Wilcoxon rank-sum test or the chi square/Fisher's exact test. Changes in CD4 will be measured as the number of cells gained with treatment. For resistance, descriptive statistics will be calculated for the presence of 184V and other NRTI mutations at any point during follow up.
All AEs will be evaluated, summarized and described as a proportion of patients presenting events, and the total number of events. DAIDS AE and grading will be used also for laboratory tests.
Safety will be assessed by the incidence of adverse events. The relative risk will be used to assess the differences between groups with respect to the incidence of adverse events. Ninety-five percent confidence intervals will be used to assess the statistical significance and precision of the relative risk estimate. All adverse events will be described using the MedDRA or WHO classification system.
Mean changes in clinical laboratory measurements and vital signs over time will be evaluated using Student's t-test or Mann – Whitney for independent samples, as required.

8.1.1 Level of Significance to be Used

All statistical tests will be considered as significant if the alpha level is below 5%. However, alpha levels below 10% will be considered as indicating a trend.

8.1.2 Procedure for Handling Missing, Unused, and Spurious Data

No imputations or replacement of missing data will be performed. Observations will be censored at the time of last follow up.

8.1.3 Procedures for Reporting Deviations from the Original Statistical Plan

Any deviations will be reported as amendments to the statistical analysis plan.

8.1.4 Criteria for Selection of Subjects to be Included in the Analyses

The primary analyses will be according to the Intent to Treat-exposed (ITT-e) principle that will be based on all patients enrolled in the study and who have received at least one dose of the study medications.

9 Direct Access to Source Data/Documents

Upon the request of the regulatory authority, IRB/IEC members, or auditors, the investigators will make all requested trial-related documents available for direct access.

10 Ethical Conduct of the Study

10.1 Informed Consent

It is the responsibility of the investigator to ensure that each subject is given adequate explanation of the aims, methods, anticipated benefits and potential risks of this study, and that each subject voluntarily signs and dates the IRB/IEC-approved informed consent form prior to study participation. The investigator must also explain that subjects have the right to refuse to participate in the study or to withdraw at any time for any reason, without prejudice with respect to future treatment.

The investigator will document in the subject’s medical record that informed consent was obtained prior to the performance of any study related procedure and will retain the original consent form with the study records. A copy of the signed and dated consent form should be given to the subject. Appendix H contains the elements of informed consent.

10.2 Conduct of the Study

The study will be conducted in accordance with the protocol, GCP, applicable clinical research regulations and guidelines, and all applicable local regulations.

11 Data Handling and Record Keeping

Electronic Case Report Forms (CRFs) will be used to store information collected during this study. CRFs will be completed for each subject enrolled in this study. All CRFs will be legible and completed. Any necessary corrections will be made by drawing a single line through the incorrect entry and writing in the revision, and they will be initialized and dated by the investigator or his/her designee. Data will not be obliterated by blacking out, use of correction fluid, or by erasing the original entry. If the reason for the correction is not obvious, a brief explanation (e.g., transcription error) will accompany the change. All information written on the CRFs will also be reflected in the subject's source documents.

12 Data Quality Assurance

In order to maintain the integrity of study data, information collected on CRFs and laboratory results will be verified by the investigator. This will be documented by the investigator’s signature.

The investigator will keep a screening/enrollment log and complete identification information on each subject to be used for the purpose of long-term follow up if needed.

13 Appendices

13.1 Appendix A. Protocol Synopsis

**Protocol Title:**	Treatment with Dolutegravir associated with Tenofovir/Lamivudine or Emtricitabine in trans women infected with HIV: a pilot study
**Study Objectives:**	The primary objective of this pilot study is to determine the retention in care of transwomen treated with DTG-TDF-FTC or 3TC.<br>Secondary objectives:<br>a. To evaluate the efficacy of the antiretroviral regimen;<br>b. To describe the safety and tolerability of this regimen;<br>c. To evaluate adherence across 48 weeks;<br>d. To determine the patient satisfaction with this regimen;<br>e. To identify individual, social and contextual factors associated with adherence and retention.
**Primary endpoint:**	The primary objective will be assessed by the proportion of individuals that provide information on ART use at the end of the study:<br>a. Retention in care: Proportion of enrolled and dosed individuals that provide clinical information up to 48 weeks of follow up.<br>b. Retention in treatment: Proportion of enrolled and dosed individuals that receive study drugs up to 48 weeks of follow up.
**Secondary endpoints:**	The secondary objectives will be evaluated using the following endpoints:<br>a. Proportion of patients with HIV-1 RNA levels of less than 50 copies/mL at week 24 and 48 weeks of treatment by the IIT-exposed snapshot FDA algorithm;<br>b. Frequency, type and severity of adverse events and laboratory abnormalities;<br>c. Pill count, analogue visual scale for adherence in each visit;<br>d. Changes in the scores of quality of life, social support, anxiety and depression scales (WBI, DUKE, HAD) at baseline, 4, 24, and 48 weeks of follow up;

	e. Through association of baseline individual, social and contextual characteristics with percentage of adherence and retention at 48 weeks.
**Patient Population:**	Subjects will be ≥18 years HIV-1 HIV treatment-naïve, self-identified as transwomen who met all inclusion criteria and none of the exclusion criteria
**Study Design:**	Prospective, open-label, single-arm trial of DTG-TDF-FTC or 3TC among ART-naïve HIV
**Regimens:**	Dolutegravir 50 mg QD plus co-formulated lamivudine 300 mg or emtricitabine 200 mg/ tenofovir 300 mg QD
**Duration:**	48 weeks
**Sample size:**	60 subjects

13.2 Appendix B. Clinical Toxicity

	GRADE 1 TOXICITY	GRADE 2 TOXICITY	GRADE 3 TOXICITY	GRADE 4 TOXICITY
**HEMATOLOGY**				
HEMOGLOBIN	9.6–10.5 g/dl	8.0–9.5 g/dl	6.5–7.9 g/dl	<6.5 g/dl
HEMOGLOBIN (S.I. Units)	96–105 g/L	80–95 g/L	65–79 g/L	<65 g/L
ABSOLUTE NEUTROPHIL COUNT	1000–1500/mm^3	750–999/mm^3	500–749/mm^3	<500/mm^3
ABSOLUTE NEUTROPHIL COUNT (S.I. Units)	1000–1500 GI/L	750–999 GI/L	500–749 GI/L	<500 GI/L
PLATELETS	75,000–99,000/mm^3	50,000–74,999/mm^3	20,000–49,999/mm^3	<20,000/mm^3or diffuse petechiae
PLATELETS (S.I. Units)	75,000–99,000 GI/L	50,000–74,999 GI/L	20,000-49,999 GI/L	<20,000 GI/L or diffuse petechiae
PT	.01–1.25 x upper limit of normal	1.26–1.5 x upper limit of normal	1.51–3.0 x upper limit of normal	3 x upper limit of normal
APTT	1.20–1.66 x upper limit of normal	0.67–2.35 x upper limit of normal	.36–3 x upper limit of normal	>3 x upper limit of normal
FIBRINOGEN	0.99–0.75 x lower limit of normal	0.74–0.50 x lower limit of normal	0.49–0.25 x lower limit of normal	<0.25 x lower limit of normal
FIBRIN SPLIT PRODUCT	20–40 µg/ml	41–50 µg/ml	51–60 µg/ml	>60 µg/ml
METHEMOGLOBIN	5.0–9.9%	10.0–14.9%	15.0–20.0%	>20%
**CHEMISTRIES**				
HYPONATREMIA	130-134 mEq/L	123–129 mEq/L	116–122 mEq/L	115 mEq/L and less or mental status changes or seizures
HYPONATREMIA (S.I. Units)	130–134 mmol/L	123–129 mmol/L	116–122 mmol/L	15 mmol/L and less or mental status changes or seizures
HYPERNATREMIA	146–150 mEq/L	151–157 mEq/L	158–165 mEq/L	165 mEq/L or mental status changes/seizures
HYPERNATREMIA (S.I. Units)	146–150 mmol/L	151–157 mmol/L	158–165 mmol/L	165 mmol/L or mental status changes/seizures
HYPOCALCEMIA (ionized calcium)	3.0–3.4 mg/dL	2.5–2.9 mg/dL replacement Rx req.	2.0–2.4 mg/dL or intensive replacement Rx Req. or hospitalization	<2 mg/dL or paresis or ileus or life–threatening arrhythmia
HYPERCALCEMIA (ionized calcium)	5.6–6.0 mg/dL	6.1–6.5 mg/dL	6.6–7.0 mg/dL	>7.0 mg/dL or paresis or ileus or life–threatening arrhythmia
TRIGLYCERIDES	-	400–750 mg/dL	751–1200 mg/dL	>1200 mg/Dl
TRIGLYCERIDES (S.I. Units)	-	4.52–8.47 mmol/L	8.48–13.55 mmol/L	>13.55 mmol/L
CHOLESTEROL	200–239 mg/dL	240–300 mg/dL	301–400 mg/dL	>400 mg/dL
CHOLESTEROL (S.I. Units)	5.16–6.19 mmol/L	>6.19–7.77 mmol/L	>7.77–10.35 mmol/L	>10.35 mmol/L
HYPOGLYCEMIA	55–66 mg/dL	40–54 mg/dL	30–39 mg/dL	30 mg/dL or mental status changes or coma
HYPOGLYCEMIA (S.I. Units)	3.03–3.69 mmol/L	2.20–3.02 mmol/L	1.64–2.19 mmol/L	1.64 mmol/L or mental status changes or coma
HYPERGLYCEMIA	116–160 mg/dL	161–250 mg/dL	251–500 mg/dL	500 mg/dL or ketoacidosis or seizures
HYPERGLYCEMIA (S.I. Units)	6.42–8.91 mmol/L	8.92–13.90 mmol/L	13.91–27.79 mmol/L	>27.79 mmol/L or ketoacidosis or seizures

HYPERURICEMIA	7.5–9.9 mg/dL	10.0–12.0 mg/dL	12.1–15.0 mg/dL	>15.0 mg/dL
HYPERURICEMIA (S.I. Units)	441–591 µmol/L	592–716 µmol/L	717–895 µmol/L	>895 µmol/L
HYPOCALCEMIA corrected for albumin	8.4–7.8 mg/dL	7.7–7.0 mg/dL	6.9–6.1 mg/dL	<6.1 mg/dL or life–threatening arrhythmia or tetany
HYPERCALCEMIA corrected for albumin	10.6–11.5 mg/dL	11.6–12.5 mg/dL	12.6–13.5 mg/dL	>13.5 mg/dL or life–threatening arrhythmia or tetany
HYPOMAGNESEMIA	1.4–1.2 mEq/L	1.1–1.0 mEq/L or replacement of Rx req.	0.9–0.6 mEq/L or intensive Rx req. hospitalization	<0.6 mEq/L or life–threatening arrhythmia
HYPOMAGNESEMIA (S.I. Units)	0.70–0.56 mmol/L	0.55–0.44 mmol/L or replacement of Rx req.	0.43–0.30 mmol/L or intensive Rx req. hospitalization	<0.30 mmol/L or life-threatening arrhythmia
HYPOPHOSPHATEMIA	2.0-2.4 mg/dL	1.5–1.9 mg/dL or replacement Rx req.	1.0–1.4 mg/dL intensive Rx req. hospitalization	<1.0 mg/dL life–threatening arrhythmia or CHF
HYPOPHOSPHATEMIA (S.I. Units)	0.63–0.79 mmol/L	0.47–0.62 mmol/L or replacement Rx req.	0.31–0.46 mmol/L intensive Rx req. hospitalization	<0.31 mmol/L life–threatening arrhythmia or CHF
HYPER–BILIRUBINEMIA	1.1–1.5 x upper limit of normal	1.6–2.9 x upper limit or normal	3–5 x upper limit of normal	>5 x upper limit of normal
HYPER–BILIRUBINEMIA (S.I. Units)	>22–31 µmol/L (Grade 1)	>31–62 µmol/L (Grade 2)	>62–103 µmol/L (Grade 3)	>103 µmol/L (Grade 4)
HYPOKALEMIA	3.0–3.4 mEq/L	2.5–2.9 mEq/L	2.0–2.4 mEq/L	<2.0 mEq/L
HYPOKALEMIA (S.I. Units)	3.0–3.4 mmol/L	2.5–2.9 mmol/L	2.0–2.4 mmol/L	<2.0 mmol/L
HYPERKALEMIA	5.6–6.0 mEq/L	6.1–6.5 mEq/L	6.6–7.0 mEq/L	>7.0 mEq/L
HYPERKALEMIA (S.I. Units)	5.6–6.0 mmol/L (Grade 1)	6.1–6.5 mmol/L (Grade 2)	6.6–7.0 mmol/L (Grade 3)	>7.0 mmol/L (Grade 4)
BUN	1.25–2.5 x upper limit of normal	2.6–5.0 x upper limit of normal	5.1–10.0 x upper limit of normal	>10 x normal
CREATININE	1.1–1.5 x upper limit of normal	1.6–3.0 x upper limit of normal	3.1–6.0 x upper limit of normal	>6.0 x upper limit of normal or requires dialysis
CPK (CK) (not related to exercise)	1.1–2.0 x upper limit of normal	2.1–4.0 x upper limit of normal	4.1–6.0 x upper limit of normal	>6.0 x upper limit of normal
**ENZYMES**				
AST/SGOT	1.25–2.5 x upper limit of normal	2.6–5 x upper limit of normal	5.1–10 x upper limit of normal	>10 x upper limit of normal
ALT/SGPT	1.25–2.5 x upper limit of normal	2.6–5 x upper limit of normal	5.1–10 x upper limit of normal	>10 x upper limit of norm
GGT	1.25–2.5 x upper limit of normal	2.6–5 x upper limit of normal	5.1–10 x upper limit of normal	>10 x upper limit of normal
ALKALINE PHOSPHATASE	1.25–2.5 x upper limit of normal	2.6–5 x upper limit of normal	5.1–10 x upper limit of normal	>10 x upper limit of normal
AMYLASE (pancreatic)	1.1–1.3 x upper limit of normal	1.4–2.0 x upper limit of normal	2.1–5.0 x upper limit of normal or mild to moderate clinical pancreatitis	5.1 x upper limit of normal or severe clinical pancreatitis
**URINALYSIS**				
PROTEINURIA	1+, 30–100 mg/dL	2+, >100–300 mg/dL	3+, >300 mg/dL	nephrotic syndrome
HEMATURIA	microscopic only, <10 RBCs	gross, no clots, 10–100 RBCs	gross, clots, > 101 RBCs	obstructive or Rx req.
**CARDIAC**				
CARDIAC RHYTHM	-	asymptomatic, transient signs, no Rx required	recurrent/persistent, no Rx required	unstable dysrhythmia requires treatment or hospitalization

HYPERTENSION	transient inc. >20 mm, no Rx	recurrent, chronic >20 mm, Rx req.	requires outpt. acute Rx	Hospitalization
HYPOTENSION	transient orthostatic hypotension, no Rx	symptoms correctable with oral fluid Rx	requires IV fluids, no hospitalization	requires hospitalization
PERICARDITIS	minimal effusion	mild/mod asymptomatic effusion no Rx	symptomatic effusion pain EKG changes	tamponade percardiocentesis or surgery required
HEMORRHAGE, BLOOD LOSS	microscopic occult	mild/no transfusion	gross blood loss, 1–2 units transfused	massive blood loss, >3 units transfused
**RESPIRATORY**				
COUGH	transient-no Rx	treatment associated cough, local non-narcotic Rx	treatment associated cough, narcotic Rx required	Uncontrolled
SHORTNESS OF BREATH	mild, does not interfere with routine activities	moderate, interferes with routine activities req. intermittent Rx	moderately debilitating requiring nasal oxygen	severe, requiring ventilatory assistance
BRONCHOSPASM ACUTE	transient, no Rx, 70%–80% of peak flow	req. Rx normalize w/bronchodilator FEV at 50% of peak flow	no normalization w/bronchodilator FEV at 50% of peak flow, retraction	cyanosis, FEV <25% of peak flow intubated
**GASTROINTESTINAL**				
STOMATITIS	mild discomfort, no limits on activity	some limits on eating, talking	eating, talking very limited	unable to drink fluids; req. IV fluids
NAUSEA	transient, mild discomfort, maintain reasonable intake	mod. discomfort, sign dec of intake, some limit of activity or decreased intake <3 days	severe discomfort, no significant food intake activities limited or minimal intake □3days	minimal fluid intake or hospitalization required
VOMITING	transient emesis, 2–3 per day or lasting <1 week	moderate emesis 4–5 per day or lasting <1week	vomiting all food/fluids in 24 hours, orthostatic hypotension or IV fluid, Rx required.	hypotensive shock hospitalization IV fluid therapy
CONSTIPATION	mild	moderate, Rx required	severe, Rx required, vomiting	distention with vomiting
DIARRHEA	mild or transient, 3–4 loose stools/day or mild diarrhea lasting <1 week	moderate or persistent; 5–7 loose stools/day or diarrhea lasting □1 week	bloody diarrhea; or orthostatic hypotension or >7 loose stools/day or IV Rx required	hypotensive shock or hospitalization required
ABDOMINAL PAIN	mild, occasional transient	moderate, transient	severe or requiring analgesic	severe with guarding peritoneal signs
**NEURO/NEUROMUSCULAR**				
NEURO CEREBELLAR	slight incoordination Dyskiadokinesis	intention tremor, dysmetria, slurred speech, nystagmus	locomotor ataxia	Incapacitated
MOOD	mild anxiety or depression	mod. anxiety or depression, therapy required	severe anxiety or depression or manic; (needs assistance)	acute psychosis; incapacitated req. hospitalization
NEUROCONTROL	confusion/agitation	mod. confusion/ agitation; some severe, min. Rx	sev. confusion/ agitation	toxic psychosis; hospitalization
MUSCLE STRENGTH	subjective weakness, no objective symptoms/signs	mild objective no dec in function	objective weakness; function limited	Paralysis
PAINFUL NEUROPATHY	mild discomfort; no therapy required	moderate discomfort persisting for >72 hours; analgesic required	severe discomfort, marked antalgic gait, narcotic analgesic required, with symptomatic improvement	incapacitating, intolerable discomfort. Not improved or unable to walk despite narcotic analgesics
MYOSITIS	minimal findings	Patients must have some measures of myositis (positive	Patients must have some measures of myositis (positive EMG or muscle	Patients must have some measures of myositis (positive EMG or muscle

		EMG or muscle biopsy) and one of the following:	biopsy) and one of the following:	biopsy) and one of the following:
		1) mild myalgias, >6 weeks requiring nonsteroidal anti-inflammatory agents	1) moderate myalgias or muscle tenderness, >6 weeks requiring non-steroidal anti-inflammatory agents	1) severe muscle pain (myalgias) not related to exercise requiring narcotics
		2) difficulty climbing stairs or rising from a sitting position but able to ambulate without assistance	2) requires some assistance with ambulation or general activities	2) muscle weakness resulting in inability to ambulate, requiring special care and assistance with mobilization
				3) acute rhabdomyolysis with muscle necrosis and edema, moderate to severe muscle weakness with inability to ambulate or mobilize self without assistance
				4) acute rhabdomyolysis associated with electrolyte imbalance in renal failure
**OTHER PARAMETERS**				
FEVER<br>oral, w/o infection, >12 hrs.	37.7–38.5C or 100.0–101.5F	38.6–39.5C or 101.6–102.9F	39.6–40.5C or 103–105F	>40.5C<br>>105F
HEADACHE	mild, no Rx therapy	transient, mod; non-narcotic Rx req.	severe, responds to initial narcotic therapy	intractable, req. repeated narcotic therapy
FATIGUE	<25% decrease in daily activities	normal activity decrease 25–50%	normal activity decrease >50%, can't work	unable to care for self
ALLERGIC REACTION	pruritus w/o rash	localized urticaria angioedema	generalized urticaria angioedema	Anaphylaxis
LOCAL REACTION	tenderness or erythema	induration <10 cm or phlebitis or inflammation	induration >10 cm or ulceration	Necrosis
MUCOCUTANEOUS	erythema, pruritus	diffuse maculopapular rash dry desquamation	vesiculation, moist desquamation ulceration	exfoliative dermatitis, mucous membrane involvement suspected, Stevens Johnson or erythema multiforme, necrosis requiring surgery

13.3 Appendix C. Disallowed Concomitant Therapy

a. Barbiturates

b. Carbamazepine

c. Oxacarbazepime

d. Phenobarbital

e. Phenytoin

f. Dofetilide

g. Rifampin

h. Metformin (metformin concentrations may be increased by dolutegravir. Subjects should be monitored and a dose adjustment of metformin may be required)

i. Products containing St John's Wort (Hypericum perforatum)

j. All disallowed medication as mentioned in the package insert of drugs included in this study

k. Methadone, due to potential for decreased plasma concentration, subjects should be monitored for signs of withdrawal and methadone dose adjusted as appropriate

l. Immunomodulators: systemic corticosteroids, interleukins, or interferons

m. Chemotherapeutic agents

n. Therapeutic vaccines for HIV

o. All investigational medications.

Chronic use of systemic (oral or parenteral) glucocorticoids must be avoided; however short treatment courses (for example 10 days or less) and topical, inhaled or intranasal use of glucocorticoids will be allowed.

13.4 Appendix D. List of Known Expected Manifestations of HIV Infection

a. Candidiasis

- *Bronchi

- *Esophagus
- *Lungs
- Oropharyngeal (Thrush)
- *Trachea
- Vulvovaginal (Persistent, Frequent, or Poorly Responsive to Therapy)
- Other Candidiasis

b. Cytomegalovirus

- *Retinitis
- *Cytomegalovirus Disease (other than liver, spleen or nodes)

c. Herpes Simplex Virus

- *Bronchitis
- *Esophagitis
- *Pneumonitis
- *Chronic Ulcer(s) (>1 month in duration)
- Other Sites

d. Lymphoma (HIV-Related)

- *Burkitt's
- *Immunoblastic
- *Primary of brain

e. Mycobacterial Disease:

- *Mycobacterium avium - intracellulare
- *Mycobacterium kansasii
- *Mycobacterium tuberculosis
- *Other Mycobacteriosis

f. Other

- *Cervical cancer, invasive
- Cervical cancer, *in situ*
- Cervical dysplasia
- *Coccidioidomycosis
- *Cryptococcosis
- *Cryptosporidiosis
- *Encephalopathy HIV-related

- Hairy leukoplakia, oral
- Herpes zoster
- *Histoplasmosis
- *Isosporiasis
- *Kaposi's sarcoma
- Listeriosis
- **Pneumocystis jirovecii* pneumonia
- *Pneumonia, recurrent
- *Progressive multifocal leukoencephalopathy
- *Salmonellosis
- *Septicemia, recurrent
- *Toxoplasmosis of the brain
- *Wasting syndrome, HIV-related

*AIDS-defining event as described by CDC Surveillance Case Definition of 1993

13.5 Appendix E. Document Required Prior to Initiation of the Study

Prior to the beginning of the clinical study, the investigators will be asked to provide the following documentation:

- An original Investigator-signed Protocol Agreement page.

- A current curriculum vitae for the investigator. If sub investigators will participate in the study, signed and dated curriculum vitae is required for each additional individual.
- A copy of the signed and dated approval letter from the Institutional Review Board (IRB)/Independent Ethics Committee (IEC), with regard to protocol, informed consent, and any advertisement(s).
- A list of IRB/IEC committee members, including their occupations and institutional affiliations.
- An approved copy of the IRB/IEC informed consent document to be used in this study.
- A list of normal reference ranges and values for all laboratory tests specified by the protocol for all laboratories utilized.
- A current copy of the laboratory(ies) certification(s) or the certification number(s), the name of the certifying authority, the period of certification, and the Laboratory Director's curriculum vitae.

13.6 Appendix F. Responsibilities of the Study Site (Fundación Huésped)

The center is responsible to Health Authorities for taking all reasonable steps to ensure the proper conduct of the Clinical Trial Protocol as regards ethics, Clinical Trial Protocol compliance, and integrity and validity of the data recorded on the Case Report Forms. Thus, the main duty of the Monitoring Team is to help the Investigator and the coordinating center

maintain a high level of ethical, scientific, technical and regulatory quality in all aspects of the Clinical Trial.

At regular intervals during the Clinical Trial, the site will be contacted, through monitoring visits, letters or telephone calls, by a representative of the Monitoring Team to review study progress, Investigator and patient compliance with Clinical Trial Protocol requirements and any emergent problems. During these monitoring visits, the following but not exhaustive list of points will be scrutinized with the Investigator: patient informed consent, patient eligibility, patient recruitment and follow-up, Serious Adverse Event documentation and reporting, Investigational Product allocation, patient compliance with the Clinical Trial Protocol and the Investigational Product regimen, Investigational Product accountability, concomitant therapy use and quality of data.

13.7 Appendix G. Responsibilities of the Clinical Investigator

a. To secure prior approval of the study by an appropriate Institutional Review Board/Independent Ethics Committee which conforms to ICH guidelines.

b. To obtain valid written informed consent from each person who participates in the study.

c. To prepare and maintain adequate case histories of all persons entered into the study, including case report forms, hospital records, laboratory results, etc., and to maintain these data for the time required by their institution.

d. To identify all sub-investigators who will also supervise drug administration.

e. To report adverse effects to the principal investigator promptly. In the event of a serious or unexpected adverse event, to notify the principal investigator immediately by telephone.

13.8 Appendix H. Elements of Informed Consent

A signed consent must be obtained prior to any study-specific activities and must include the following items:

a. A statement that the study involves research, and an explanation of the purpose of the research and the expected duration of the patient's participation, a description of the

procedures to be followed, and identification of any procedures that are experimental and/or invasive.

b. A description of the study, including trial treatments and the probability for random assignment to treatment.

c. A description of any reasonably foreseeable risks, inconveniences or discomforts to the patients and, if applicable, to an embryo, fetus or nursing infant.

d. A description of the subject's responsibilities.

e. A description of any benefits to the patient or to others which may reasonably be expected from the research. If the patient is to be compensated for participating in the study, the consent form must describe what the compensation consists of (to assure neither coercion nor undue influence).

f. A disclosure of appropriate alternative procedures or courses of treatment, if any, that might be advantageous to the patient, and the potential risks and benefits.

g. A statement that the investigator and the Institutional Review Board (IRB)/Independent Ethics Committee (IEC), will be granted direct access to the subject's original medical records for verification of clinical trial procedures and/or data, without violating the confidentiality of the subject, to the extent permitted by the applicable laws and regulations and that, by signing a written informed consent form, the subject or the subject's legally acceptable representative is authorizing such access.

a. A statement that records identifying the subject will be kept confidential and, to the extent permitted by the applicable laws and/or regulations will not be made publicly available. If the results of the trial are published, the subject's identity will remain confidential.

h. An explanation of whom to contact for answers to pertinent questions about the research and research patients' rights, and whom to contact in the event of a research-related injury to the patient. (NOTE: It is preferable to identify some person other than the investigator as the contact. The guidance of the IRB/IEC may be required.)

i. A statement that participation is voluntary, that refusal to participate will involve no penalty or loss of benefits to which the patient is otherwise entitled, and that the patient may discontinue participation at any time without penalty or loss of benefits to which the patient is otherwise entitled.

j. A statement that a signed and dated copy of the consent form will be given to the patient.

k. A statement of the agreement to participate (e.g., “I agree to participate ...”).

l. A place for signature and date of signature for the research patient (or legally authorized representative) and for the person who explained the nature of the study to the patient (investigator or investigator’s representative).

m. *A statement that the particular treatment or procedures may involve risks to the patient that are currently unforeseeable.

n. A statement of anticipated circumstances or reasons under which the patient’s participation may be terminated by the investigator without regard to the patient's consent.

o. A statement of any additional costs to the patient that may result from participation in the research.

p. A statement regarding the consequences of a patient’s decision to withdraw from the research and procedures for orderly termination of participation by the patient.

q. A statement that significant new findings developed during the course of the research which may relate to the patient’s willingness to continue participation will be provided to the patient (or the patient’s legally acceptable representative) in a timely manner.

r. A statement of the approximate number of patients involved in the study.

13.9 Appendix I: Questionnaires of Adherence ACTG

ADHERENCE QUESTIONNAIRE

Subject # __ __ __ __ __ __ __ **Visit Date __ __ / __ __ __ /__ __**

Subject Initials __ __ __ **Week # __ __**

This questionnaire asks about study medications that you took recently. Many people find it hard to always remember their pills. For example,

- *Some people get busy and forget to carry their pills with them.*

- *Some people find it hard to take their pills according to special instructions, such as to increase the amount of medication you are taking if you have a herpes flare.*
- *Some people decide to skip pills to avoid side effects or to just not be takingpills that day.*

We need to understand how people with HIV are really doing with their pills. Please tell us what you are ***actually*** *doing. Don't worry about telling us if you don't take all your pills.*
We need to know what is really happening, not what you think we "want to hear."

1. How many doses of your study medication did you miss…

Yesterday? __________ dose(s)

The day before yesterday (2 days ago)? __________ dose(s)

3 days ago? __________ dose(s)

4 days ago? __________ dose(s)

If you did not miss any doses, write a zero (0) in the space provided.

2. This study medication needs to be taken 2 times a day. How closely did you follow this specific schedule over the last four days?

Never	Some of the time	About half of the time	Most of the time	All the time
☐	☐	☐	☐	☐
0	1	2	3	4

3. Did study staff give you special instructions to take extra medication because of a herpes flare in the last four days?

Yes	No
☐	☐
1	2

If Yes, how often did you follow those special instructions over the last four days?

Never	Some of the time	About half of the time	Most of the time	All the time
☐	☐	☐	☐	☐
0	1	2	3	4

4. Some people find that they forget to take their pills on the weekend days. Did you miss any of your study medications last weekend - last Saturday or Sunday?

Yes	No
☐	☐
1	2

5. Some people find that they forget to take their pills on the weekend days. How often did you miss any of your study medications on a weekend during the past 3 months?

Never	Some of the time	About half of the time	Most of the time	All the time
☐	☐	☐	☐	☐
0	1	2	3	4

When was the last time you missed any of your medications? *(Check one box)*

Within the past week	1-2 weeks ago	2-4 weeks ago	1-3 months ago	more than 3 months ago	Never
☐	☐	☐	☐	☐	☐
5	4	3	2	1	0

14 References

1. Baral SD, Poteat T, Stromdahl S, Wirtz AL, Guadamuz TE, Beyrer C. Worldwide burden of HIV in transgender women: a systematic review and meta-analysis. The Lancet Infectious diseases. 2013 Mar;13(3):214-22. PubMed PMID: 23260128.
2. Poteat T, Reisner SL, Radix A. HIV epidemics among transgender women. Current opinion in HIV and AIDS. 2014 Mar;9(2):168-73. PubMed PMID: 24322537.

3. Operario D, Nemoto T. HIV in transgender communities: syndemic dynamics and a need for multicomponent interventions. Journal of acquired immune deficiency syndromes. 2010 Dec;55 Suppl 2:S91-3. PubMed PMID: 21406995. Pubmed Central PMCID: 3075534.

4. Kerr T SE, Sued O. HIV Infection among Transgender Women: Challenges and Opportunities. J AIDS Clin Res. 2014.

5. Nemoto T, Operario D, Keatley J, Villegas D. Social context of HIV risk behaviours among male-to-female transgenders of colour. AIDS care. 2004 Aug;16(6):724-35. PubMed PMID: 15370060.

6. Reback CJ FJ. HIV prevalence, substance use, and sexual risk behaviors among transgender women recruited through outreach. AIDS and Behavior. 2014.

7. Nemoto T, Bodeker B, Iwamoto M. Social support, exposure to violence and transphobia, and correlates of depression among male-to-female transgender women with a history of sex work. American journal of public health. 2011 Oct;101(10):1980-8. PubMed PMID: 21493940. Pubmed Central PMCID: 3222349.

8. Dirección Nacional de Sida, Boletin epidemiológico 2010. Available at http://www.msal.gov.ar/sida/pdf/investigaciones/resumen_ejecutivo.pdf.

9. Carobene M, Bolcic F, Farias MS, Quarleri J, Avila MM. HIV, HBV, and HCV molecular epidemiology among trans (transvestites, transsexuals, and transgender) sex workers in Argentina. Journal of medical virology. 2014 Jan;86(1):64-70. PubMed PMID: 24123155.

10. Dos Ramos Farias MS, Garcia MN, Reynaga E, Romero M, Vaulet ML, Fermepin MR, et al. First report on sexually transmitted infections among trans (male to female transvestites, transsexuals, or transgender) and male sex workers in Argentina: high HIV, HPV, HBV, and syphilis prevalence. International journal of infectious diseases : IJID : official publication of the International Society for Infectious Diseases. 2011 Sep;15(9):e635-40. PubMed PMID: 21742530.

11. Socias M, Marshall B, Aristegui I, Romero M, Cahn P, Kerr T, et al. Factors associated with healthcare avoidance among transgender women in Argentina. International journal for equity in health. 2014 Sep 27;13(1):81. PubMed PMID: 25261275. Pubmed Central PMCID: 4220051.

12. Socias ME, Marshall BD, Aristegui I, Zalazar V, Romero M, Sued O, et al. Towards full citizenship: correlates of engagement with the gender identity law among transwomen in

Argentina. PloS one. 2014;9(8):e105402. PubMed PMID: 25133547. Pubmed Central PMCID: 4136870.

13. Cohen MS, McCauley M, Gamble TR. HIV treatment as prevention and HPTN 052. Current opinion in HIV and AIDS. 2012 Mar;7(2):99-105. PubMed PMID: 22227585. Pubmed Central PMCID: 3486734.

14. Young I, Flowers P, McDaid LM. Key factors in the acceptability of treatment as prevention (TasP) in Scotland: a qualitative study with communities affected by HIV. Sexually transmitted infections. 2014 Dec 7. PubMed PMID: 25482649.

15. Wilson EC, Garofalo R, Harris RD, Herrick A, Martinez M, Martinez J, et al. Transgender female youth and sex work: HIV risk and a comparison of life factors related to engagement in sex work. AIDS and behavior. 2009 Oct;13(5):902-13. PubMed PMID: 19199022. Pubmed Central PMCID: 2756328.

16. Keller K. Transgender health and HIV. BETA : bulletin of experimental treatments for AIDS : a publication of the San Francisco AIDS Foundation. 2009 Summer-Fall;21(4):40-50. PubMed PMID: 20034245.

17. Socías ME SO, Frola C, Iacchetti A, Kerr T, Arístegui I, Zalazar V, Pérez H, Cahn P. Engagement in the HIV care cascade among transgender women enrolled in a public HIV clinic in Buenos Aires, Argentina, 2000-2012. 20th International AIDS Conference (AIDS 2014); July, 20-25; Melbourne, Australia2014. 2014.

18. Clotet B, Feinberg J, van Lunzen J, Khuong-Josses MA, Antinori A, Dumitru I, et al. Once-daily dolutegravir versus darunavir plus ritonavir in antiretroviral-naive adults with HIV-1 infection (FLAMINGO): 48 week results from the randomised open-label phase 3b study. Lancet. 2014 Jun 28;383(9936):2222-31. PubMed PMID: 24698485.

19. Masho SW, Wang CL, Nixon DE. Review of tenofovir-emtricitabine. Therapeutics and clinical risk management. 2007 Dec;3(6):1097-104. PubMed PMID: 18516268. Pubmed Central PMCID: 2387297.
